# Identification and Characterization of Seminal Fluid Proteins in the Asian Tiger Mosquito, *Aedes albopictus*

**DOI:** 10.1371/journal.pntd.0002946

**Published:** 2014-06-19

**Authors:** Kathryn E. Boes, José M. C. Ribeiro, Alex Wong, Laura C. Harrington, Mariana F. Wolfner, Laura K. Sirot

**Affiliations:** 1 Department of Biology, College of Wooster, Wooster, Ohio, United States of America; 2 Vector Biology Section, Laboratory of Malaria and Vector Research, National Institute of Allergy and Infectious Diseases, Rockville, Maryland, United States of America; 3 Department of Biology, Carleton University, Ottawa, Ontario, Canada; 4 Department of Entomology, Cornell University, Ithaca, New York, United States of America; 5 Department of Molecular Biology and Genetics, Cornell University, Ithaca, New York, United States of America; Johns Hopkins Bloomberg School of Public Health, United States of America

## Abstract

The Asian tiger mosquito (*Aedes albopictus*) is an important vector for pathogens that affect human health, including the viruses that cause dengue and Chikungunya fevers. It is also one of the world's fastest-spreading invasive species. For these reasons, it is crucial to identify strategies for controlling the reproduction and spread of this mosquito. During mating, seminal fluid proteins (Sfps) are transferred from male mosquitoes to females, and these Sfps modulate female behavior and physiology in ways that influence reproduction. Despite the importance of Sfps on female reproductive behavior in mosquitoes and other insects, the identity of Sfps in *Ae. albopictus* has not previously been reported. We used transcriptomics and proteomics to identify 198 Sfps in *Ae. albopictus.* We discuss possible functions of these Sfps in relation to *Ae. albopictus* reproduction-related biology. We additionally compare the sequences of these Sfps with proteins (including reported Sfps) in several other species, including *Ae. aegypti*. While only 72 (36.4%) of *Ae. albopictus* Sfps have putative orthologs in *Ae. aegypti*, suggesting low conservation of the complement of Sfps in these species, we find no evidence for an elevated rate of evolution or positive selection in the Sfps that are shared between the two *Aedes* species, suggesting high sequence conservation of those shared Sfps. Our results provide a foundation for future studies to investigate the roles of individual Sfps on feeding and reproduction in this mosquito. Functional analysis of these Sfps could inform strategies for managing the rate of pathogen transmission by *Ae. albopictus*.

## Author Summary

The highly invasive Asian tiger mosquito (*Aedes albopictus*) transmits several pathogens that cause disease in humans and other animals. Therefore, *Ae. albopictus* poses a large and growing threat to public health across the world. One step toward managing the reproduction and threat of this species is to determine factors that influence its reproductive biology. Seminal fluid proteins (Sfps) are transferred from male mosquitoes to females during mating, and receipt of Sfps changes female reproductive behavior and physiology. Here we report the identity of 198 *Ae. albopictus* Sfps. We discuss the potential roles and impacts of these Sfps on reproduction. In addition, we compare *Ae. albopictus* Sfps with proteins (including reported Sfps) from other species, including two other important mosquito vectors of pathogens that cause human diseases. Our results provide a foundation for future studies to investigate the roles of individual Sfps on *Ae. albopictus* reproduction.

## Introduction

*Aedes albopictus*, the Asian tiger mosquito, is an important species from both an epidemiological and an ecological perspective. Epidemiologically, it has the potential ability to transmit over 20 viruses [1], [2], and it plays a significant and growing role across the world as an important vector of several pathogens including those that cause dengue and Chikungunya fevers [2]–[5]. Ecologically, *Ae. albopictus* is considered to be one of the world's fastest-spreading invasive animal species [6]. While native to East Asia, it has recently colonized every continent except Antarctica (most recently reviewed by [5]), and its range is expected to grow in the future [2], [7], [8]. The impact of this range expansion on disease spread is difficult to predict [9], [10], but it will likely pose additional threats to public health [5]. Consequently, there is an urgent need to develop effective strategies for controlling the reproduction and spread of *Ae. albopictus* [11].

One step toward managing the reproduction of *Ae. albopictus* is to investigate seminal fluid proteins (Sfps), which are proteins that males transfer to females during mating. Sfps in insects are crucially important for male reproductive success, and they modulate several aspects of female post-mating behavior and physiology [12], [13]. In *Ae. albopictus*, receipt of Sfps bolsters egg development under poor blood feeding conditions [14], increases egg laying [15], and inhibits female remating [16], [17]. Interestingly, some of these Sfp-induced effects can last throughout the life of the female, even when she receives a only a very small dose of Sfps [17]. Since Sfps modify female behavior so drastically, their identification and functional characterization may provide promising targets for the control of insects that transmit disease-causing organisms [18]–[20]. 

Sfps have been identified for a number of insects, including *Drosophila melanogaster* [21]–[23], medflies [24], [25], and species of sand flies [26], honey bees [27], butterflies [28], [29], flour beetles [30], and crickets [31]–[34]. Sfps have recently been identified in two mosquito species, the malaria vector *Anopheles gambiae* [19], [20], [35] and the yellow fever mosquito *Aedes aegypti* [36]. Identifying the Sfps found in particular mosquito species can pave the way for investigations of reproduction-related roles played by individual Sfps [19]. This knowledge, together with comparisons of Sfps across species, might lead to novel or improved control strategies for these mosquitoes [20], including *Ae. albopictus*. 

Here we use transcriptomic and proteomic approaches to identify and characterize *Ae. albopictus* Sfps. We used an isotope labeling technique from Findlay et al. [22] and adapted by Sirot et al. [36] for mosquitoes to identify male proteins in mated females after copulation. We identified the transferred proteins by comparing the mass spectra of proteins in our samples against the spectra from a predicted protein database. This predicted protein database was generated from sequenced transcriptomes of the male and female reproductive tract. Using this technique, we identified 198 *Ae. albopictus* putative Sfps.

## Methods

### Overview

Our methods are very similar to those of our recent study in *Ae. aegypti* [36]. We therefore note methodological similarities where appropriate and focus primarily on differences in our methodology. We describe the methods we used to sequence the transcriptomes of the male and female reproductive tract and generate the predicted protein database in the supporting information (Text S1).

### Mosquito rearing and mating

As in our study of *Ae. aegypti* [36], to distinguish male-derived proteins from the female proteins in the reproductive tract of mated females, we adapted a stable-isotope labeling method originally used for *D. melanogaster* Sfp identification [22]. Stable-isotope labeling of proteins shifts the mass to charge ratio of the peptides such that they are unidentifiable by mass spectrometry because the observed spectra do not match predicted spectra generated from a protein database. Therefore, to identify only the male-derived proteins in mated females, we mated males reared on a standard diet to females reared on stable-isotope labeled yeast diet (^15^N-labeled yeast). To verify the effectiveness of the labeling, we reared as controls two groups of females: virgin females reared on the stable-isotope labeled yeast diet, and virgin females reared on an unlabeled diet.

*Aedes albopictus* (New Jersey strain) were used for our study and were reared as described previously [36]. Pupae were placed into individual vials until they emerged as adults to ensure the virgin mating status of all individuals used in the experiment. Adult females were housed in 5 L bucket cages containing of up to 70 females from the same treatment (15N-labeled or unlabeled diet), and adult males were housed in 5 L bucket cages of up to 50 males. All adults were given free access to a 20% sucrose solution.

For mating, each ^15^N-labeled female (4–6 days post-eclosion) was transferred into a cage containing 40–50 unlabeled males (4–5 days post-eclosion). Matings lasted for no longer than three minutes, and when the pair began to separate at the end of mating, the female was collected and placed on ice (for no more than 10 minutes) until dissection.

### Dissections to obtain protein samples

To obtain ^15^N-labeled mated female tissue samples, the reproductive tract below the ovaries was dissected out in 20 µl Dulbecco's PBS (DPBS) with protease inhibitors (Roche Complete Protease Inhibitor Tablets, Indianapolis, IN). Two sample types were collected (supernatant and pellet), each of which was obtained using two independent biological replicates that consisted of tissues from 17 to 20 mated females. To verify the effectiveness of our labeling technique, the reproductive tract below the ovaries was dissected from 20 ^15^N-labeled virgin females and 24 unlabeled virgin females (3–5 days post-eclosion). Samples were prepared and stored as in [36].

To distinguish Sfps from sperm proteins, we obtained sperm-enriched samples from the testes and seminal vesicles (where sperm are stored) for proteomic analyses following the methods described in [36]. Testes were dissected out from 40 virgin males (3–8 days post-eclosion), and seminal vesicles were dissected from an independent set of 30 virgin males (3–8 days post-eclosion). It is important to note that our transcriptome did not include testes tissues, so the proteins we identify in the testes samples are ones that are present both in the testes as well as the seminal vesicles and/or accessory glands.

Proteins from the samples were separated using gel electrophoresis on one-dimensional 4–20% polyacrylamide Mini-Protean TGX precast gels (Bio-Rad Laboratories, Hercules, CA), and gels were stained using SimplyBlue SafeStain (Invitrogen, Carlsbad, CA). Each gel lane was then divided into several bands (six to eight) in order to maximize sensitivity of protein identification using mass spectrometry, and to facilitate estimation of the molecular weights of the identified proteins. All bands (i.e. the entire lane for each sample) were submitted for analysis.

### Protein identification by nanoLC-MS/MS analyses, verification and selection

Proteins were identified through nanoLC-MS/MS analysis followed by comparison of the observed spectra to those generated from our transcriptome-based *Ae. albopictus* predicted protein database (see Text S1 for information on the methods used to develop this predicted protein database). These analyses were performed at the Cornell University Proteomics and Mass Spectrometry Core facility. 1D gel bands were subjected to in-gel trypsin digestion/extraction and lyophilized. Tryptic peptides were reconstituted in 2% ACN with 0.5% FA for nanoLC-ESI-MS/MS analysis on an LTQ-Orbitrap Velos Mass Spectrometer (Thermo-Fisher Scientific, San Jose, CA) equipped with a “CorConneX” nano ion source device (CorSolutions LLC, Ithaca, NY) and coupled to an UltiMate3000 RSLCnano chromatograph (Thermo).

All MS and MS/MS spectra were processed using Proteome Discoverer 1.3 (Thermo) and the raw data were exported as MGF files for subsequent database searching using Mascot Daemon (version 2.3.02, Matrix Science, Boston, MA). The acquired spectra were searched against our custom *Ae. albopictus* transcriptome-based database containing 29,503 protein sequence entries with one missed trypsin cleavage allowed. Peptide mass tolerance was set to 20 ppm and MS/MS mass tolerance was set to 0.8 Da. Carbamidomethylation of cysteine was set as a fixed modification, oxidation of methionine as well as deamidation of asparagine and glutamine were set as variable modifications. A false discovery rate was estimated as described previously [36]. A peptide was considered to be a high quality peptide only if it met all of the following criteria: at or above the 99% confidence threshold, peptide score ≥31 that was also at or above the identity threshold level, expectation value ≤0.001, and delta mass score ≤5 ppm.

The mass spectrometry results were screened against our predicted protein database to identify proteins of high confidence of being transferred from males to females during mating. For the mated female samples (supernatant and pellet samples), an identified protein was considered as “high confidence” if it had hits to the predicted spectra from either two high quality peptides in the same sample or one high quality peptide in two independent biological replicates. For proteins found in male samples (seminal vesicles and testes samples), a protein was considered a high confidence protein if it had a hit from one high quality peptide, since only one biological replicate was analyzed for each of these two tissue types.

To verify that our labeling technique was effective, the number of proteins identified in the reproductive tracts of unlabeled virgin females was compared with the number identified in labeled virgin females [36]. Using the criteria of one high quality peptide hit (see above) for a high confidence protein, 573 proteins were identified in the unlabeled virgin females but only six proteins (Aa-3848, Aa-15006, Aa-35743, Aa-38093, Aa-63600, Aa-136683) in the labeled virgin females. Since our labeling technique was 99% effective in masking proteins from labeled females, proteins we identified from the labeled mated females are most likely male-derived.

Any high confidence hit proteins in the mated female samples were then classified as being putative sperm proteins or Sfps based on which male samples they were also found in. Specifically, proteins identified in sperm-enriched samples from both the testes and seminal vesicle samples were classified as putative sperm proteins. Otherwise, proteins were classified as Sfps. Three proteins in the mated female samples were found in both the sperm-enriched testes and the seminal vesicle samples but were also identified in the labeled virgin female samples (see above; Aa-35743, Aa-38093, Aa-136683), so were not classified as putative sperm proteins.

Several of the high confidence proteins had very similar sequences and therefore likely represent products of either different alleles of a single gene or conserved gene duplicates. Since our intention is only to report Sfps that are likely to be functionally unique, similar protein sequences were grouped into clusters using BLASTp. Proteins were placed into the same cluster if: a) they were within a minimum of 50% of the length of each other, b) they had a degree of similarity equal or larger than 91%, and c) if the extent of the match was at least 80% of the size of the smaller sequence. Only one protein from each cluster was reported, which was the protein that was most abundant in our proteomics study had the highest number of total peptide hits across all of our samples. If two proteins within a cluster had an equal number of hits, we reported the protein whose sequence began with methionine, and secondarily the protein with the highest number of reads in our transcriptome data. In all but two cases, all proteins within the same cluster had the same classification as being a sperm protein or Sfp in our study. For the two cases where proteins within a cluster were classified as sperm proteins and Sfps, the reported proteins were classified as “unknown Sfp.”

In order to identify other candidate *Ae. albopictus* Sfps, we also searched our mass spectra against the predicted protein database based on the *Ae. aegypti* genome. The methods and results of this search are presented in the supporting information (Text S2).

### Functional annotation of *Ae. albopictus* seminal fluid proteins

Functional characterization of the proteins was performed by a program taking a vocabulary of ∼250 words and comparing it to matches on several databases, as previously described [37]. Based on these matches, proteins were classified into one of the following categories: cytoskeletal, extracellular matrix, metabolism (including oxidant and detoxification), immunity, hormones, proteolysis regulators (includes proteases and protease inhibitors), signal transduction, transporters and protein export machinery, RNA and protein synthesis (includes transcription factors, transcription machinery, and protein synthesis). Proteins that were classified in a different category were classified as “other” (includes bacterial product, nuclear export, nuclear regulation, protein modification, proteasome machinery, transposable element, salivary, storage, viral product). Proteins that were not assigned to a function were classified as “unknown.” Annotations of these categories and of protein classes were reviewed manually by J.M.C.R.

### Determining sequence similarity with proteins from other species

Putative orthologs of the *Ae. albopictus* Sfps were identified by comparing sequence similarity to proteins from full predicted protein sets from seven species: *Ae. aegypti* (Vectorbase AaegL1.4), *Anopheles gambiae* (Vectorbase AgamP3.7), *Culex quinquefasciatus* (Vectorbase CpipJ1.3), *Drosophila melanogaster* (NCBI), *Apis mellifera* (NCBI), *Mus musculus* (Ensembl), and *Homo sapiens* (Uniprot). A reference predicted protein set for *Ae. albopictus* was created by combining several available transcriptomes for the species: the reproductive tract transcriptome (reported in this paper), the sialome [38], and the oocyte/embryo/pharate larval transcriptome [39]. To account for potentially high redundancy across transcripts with different lengths, the transcripts in this combined database were clustered at the 99% identity level using the standalone version of the program CDHit [40], [41]. Protein sequences were defined as orthologs if they were reciprocal-best BLASTp hits (at the CDHit cluster level for *Ae. albopictus*) having an E-value<0.001.

To determine whether the predicted orthologs were previously reported as Sfps or sperm proteins, the orthologs were cross-referenced with published lists of Sfps and sperm proteins in their respective species: *Ae. aegypti* (Sfps and sperm: [36]), *An. gambiae* (Sfps: [19], [20], [35]), *D. melanogaster* (Sfps: [21]–[23]; sperm: [42], [43]), *A. mellifera* (Sfps: [27]; sperm: [44]), *M. musculus* (Sfps: [45], [46] sperm: [42]), and *H. sapiens* (Sfps: [47]; sperm: [48]). We were unable to classify any orthologs in *Cx. quinquefasciatus* as Sfps or sperm proteins, and any orthologs in *An. gambiae* as sperm proteins, since lists of those proteins have not yet been published for those species.

### Assessing evolutionary change in *Ae. albopictus and Ae. aegypti* seminal fluid proteins

A dN/dS analysis was performed to estimate and compare rates of evolutionary change in putative housekeeping genes and in seminal fluid proteins in *Ae. albopictus* and *Ae. aegypti*.

#### Selection of housekeeping genes in *Ae. Albopictus*

Putative housekeeping genes were selected from our *Ae. albopictus* transcriptome sequences by using genes that were expressed in both males and females, within the range of sequence length of the Sfps (57 to 2088 amino acids), and excluding genes whose products were detected as transferred to the females during mating. To avoid redundancy, that set of genes was clustered at the 91% identity level (see methods above), and the longest gene was selected as a representative housekeeping gene. If two genes within a cluster were the same length, then the gene with the highest total ESTs was selected as a housekeeping gene; otherwise a random gene from the cluster was selected. This method resulted in the identification of 13,903 non-redundant putative housekeeping genes, of which 3498 had a putative ortholog in the full proteome of *Ae. aegypti*. Orthology was determined as described above in the section “Determining sequence similarity with proteins from other species.”

#### Alignment and dN/dS estimation

Pairwise sequence alignments and estimates of dN/dS were conducted for all 72 Sfps having orthologs in *Ae. aegypti* (see above for orthology criteria) and 3495 of the 3498 control genes (with three control genes left out due to unreliable alignments). PRANK [49] was used to generate codon alignments for putative orthologs, since PRANK outperformed other alignment methods in a recent simulation study [50]. Maximum-likelihood estimates of pairwise dN, dS, and dN/dS were obtained using yn00, included in the PAML package [51].

#### Inferences of positive selection

For inferences of positive selection using codeml [51], PRANK was used to generate codon alignments for all 46 *Ae. albopictus* Sfp genes for which putative orthologs could be found in *Ae. aegypti*, *Cx*. *quinquefasciatus*, and *An. gambiae.* Molecular evolutionary parameters were estimated using codeml under four models: M0, M7, M8a, and M8 [51], where the “neutral” M7 and M8a models are nulls for the “selection” M8 model.

## Results and Discussion

### Summary of transcriptome

We identified 881,128 expressed-sequence tags (ESTs), which were assembled into 29,503 contigs (hereafter “transcripts”) representing possible gene products (Table 1). Of the tissues we sequenced transcripts from, 13.5% of the transcripts are found exclusively in the male reproductive tract (hereafter males), 25.7% are found exclusively in the female reproductive tract (hereafter females), and 60.8% have ESTs in the reproductive tracts of both males and females (hereafter males and females).

**Table 1:** Summary of transcriptome sequence datasets of *Ae. albopictus* reproductive tissues.

Reproductive tract^a^	Number of transcripts	Number (%) of transcripts that encode secreted peptides	Number of ESTs	Number of ESTs per number of transcripts	% total transcripts	% total ESTs
**Male only**	3,980	938 (23.6%)	139,310	35.0	13.5%	15.8%
**Female only**	7,576	1,447 (19.1%)	37,835	5.0	25.7%	4.3%
**Both male and female**	17,947	1,308 (7.3%)	703,301 (288,306 reads in males; 414,995 reads in females)	39.2	60.8%	79.8%
**Total**	**29,503**	**3,693**	**880,446**			

a Male: transcripts from virgin male accessory glands and seminal vesicles; Female: transcripts from virgin female reproductive tract below ovaries; Both: transcripts from both the male and female reproductive tissues.

**Figure 1:**
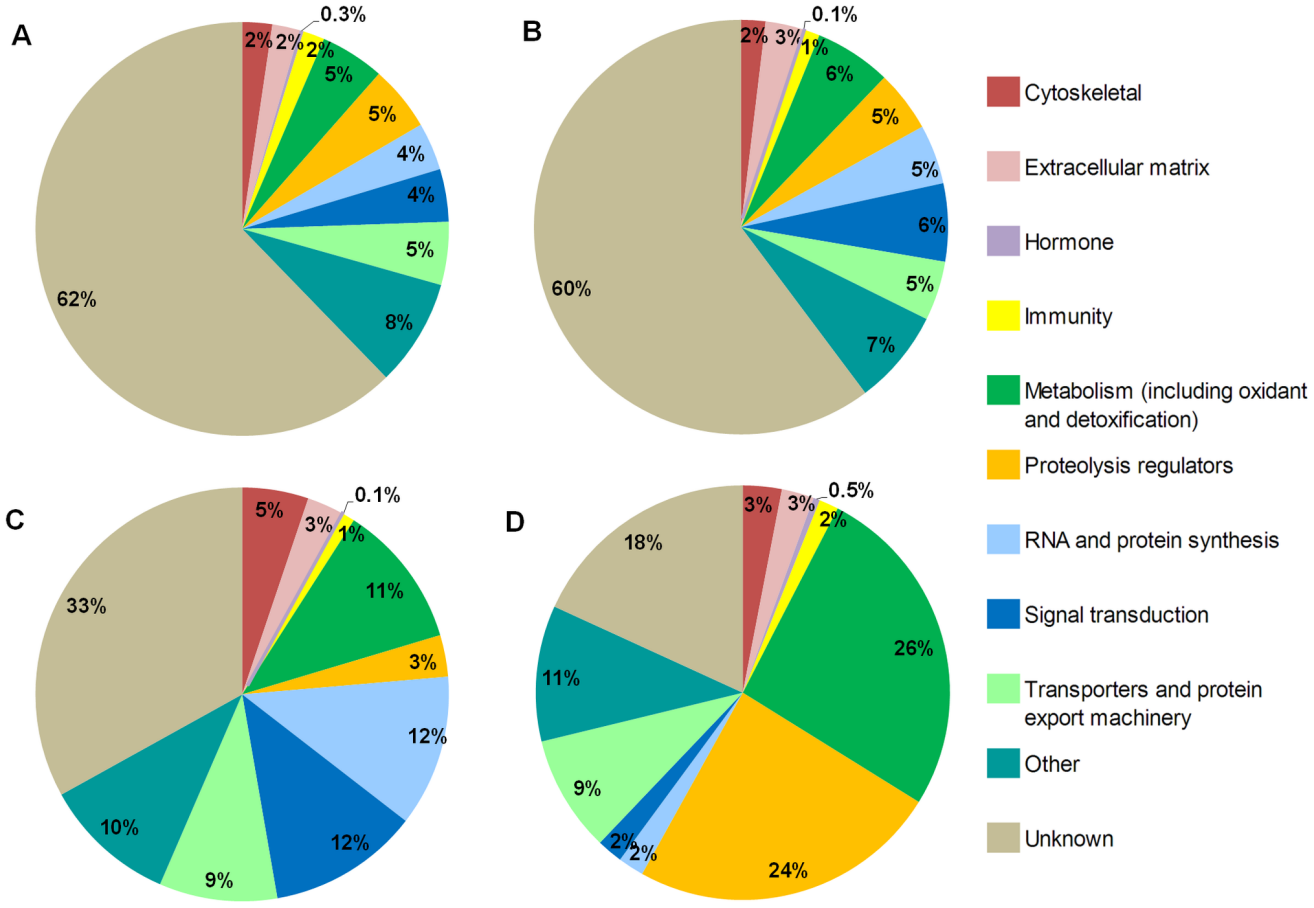
Functional categories of *Aedes albopictus* lower reproductive tract transcripts and seminal fluid proteins. Numbers indicate the percent of transcripts associated with the functional category. A. Transcripts found only in males (accessory glands and seminal vesicles; 3,980 total); B. Transcripts found only in females (lower reproductive tract; 7,576 total); C. Transcripts found in both males and females (17,947 total); D. Seminal fluid proteins (198 total).

The transcripts encode proteins that represent a wide array of functional categories (Figure 1A-C). The largest single group of transcripts found in the male, the female, and in both males and females could not be assigned any particular category (“unknown”). Other abundant functional categories included metabolism, proteolysis regulators, RNA and protein synthesis, signal transduction, and transporters and protein export machinery. Some of the less abundant functional categories included cytoskeletal, extracellular matrix, hormones, and immunity.

The distributions of transcripts across the functional classes are strikingly similar between the male-specific and female-specific sequences (Figure 1A & B). These distributions stand in marked contrast to that of the transcripts found in both males and females (Figure 1C). This difference is primarily due to the approximately halved proportion of transcripts encoding proteins with an unknown function in both male and female tissues compared to that of male-specific and female-specific tissues.

Overall, 3,693 (12.5%) of the transcripts are classified as encoding secreted proteins. Interestingly, sex-specific transcripts (those expressed only in males or only in females) are significantly more likely to encode secreted proteins than are transcripts found in both sexes (χ^2^ = 1,143.0, df = 1, p<0.001).

### Proteins transferred to females during mating: Overview

We identified a total of 314 proteins that are made in male *Ae. albopictus* and are transferred to females during mating. Of these, 198 are putative seminal fluid proteins (Figure 1D; Table S1), based on the criteria that they were not found in both the testes and the seminal vesicles. The remaining 116 transferred proteins are putative sperm proteins (Table S2), based on the criteria that they were found in both the testes and seminal vesicles. These putative sperm proteins likely are a very limited subset of all sperm proteins in *Ae. albopictus*, as our predicted protein database was derived from the male accessory gland and seminal vesicles, and did not include transcripts from the testes. We therefore focus our paper on the 198 putative seminal fluid proteins. The amino acid sequences for all proteins reported in this paper are in Table S3, Table S4, and Table S5.

It is interesting to note that the majority of the *Ae. albopictus* Sfps (134, or 67.7%) are derived from transcripts found in both the male and female reproductive tracts, whereas the remaining one-third (64, or 32.3%) are derived from transcripts found exclusively in males (Table S1). This finding highlights the benefit of using a proteomics approach to identify Sfps rather than relying on the criteria of highly male-biased or male-specific expression. It further highlights the potential existence of proteins that might function in the reproductive tract of virgin females but are additionally transferred by males as Sfps during mating (see [52]).

Proteolysis regulators (proteases and protease inhibitors) commonly comprise a large subset of the Sfps in other insects and in mammals (reviewed by [53]). In *Ae. albopictus*, 48 of the 198 Sfps (24%) are predicted proteolysis regulators, which is similar to the percentage reported in other species (for example, 20% of *D. melanogaster* Sfps and 14% of *Ae. aegypti* Sfps are predicted proteolysis regulators [22], [36]). Proteolysis regulators generally play roles in activating and regulating proteins, and potentially coordinate actions of multiple Sfps [20], [53]. Studies of proteolysis regulators in the Sfps of other species have revealed important functions related to reproduction, including roles in egg production, semen coagulation, sperm storage and activation, fertility, and protecting the female against infections, and pathogen transmission (reviewed in [53], [54]).

In the following sections we discuss the sequence similarity of the *Ae. albopictus* Sfps with proteins (including reported Sfps) from other species, highlight potential functions of Sfps relevant to *Ae. albopictus* reproductive biology, and compare the evolutionary rates of the Sfps with those of housekeeping genes.

### Seminal fluid proteins: Sequence comparisons with proteins in other species

To identify orthologs to the *Ae. albopictus* Sfps, we compared each Sfp to proteins in the full proteomes of seven species: the mosquitoes *Ae. aegypti*, *Cx. quinquefasciatus*, and *An. gambiae,* the pomace fly *D. melanogaster*, the honey bee *A. mellifera,* the mouse *M. musculus,* and humans (*H. sapiens)*. The summary of orthology information is presented in Table 2. Of the 198 *Ae. albopictus* Sfps, 93 (47.0%) have a putative ortholog in the full proteome of at least one of these species (Table S1). There is some conservation in protein sequences across these seven species. Specifically, 72 *Ae. albopictus* Sfps (36.4%) are conserved in *Ae. aegypti*, 46 (23.2%) are conserved in all three mosquito species, 43 (21.7%) are conserved in Dipteran species, 37 (18.7%) are conserved in insects, and 30 (15.2%) are conserved in all seven species examined (Table S1).

**Table 2:** Number of *Ae. albopictus* Sfps having putative orthologs in each of the seven species used for sequence similarity comparisons.

Species	Number (%) of *Ae. albopictus* Sfps with ortholog in full proteome of species	Number (%) of *Ae. albopictus* Sfps having an ortholog that is a reported Sfp for species	Number (%) of *Ae. albopictus* Sfps having an ortholog that is a reported sperm protein for species
*Aedes aegypti*	72 (36.4%)	18 (9.1%)	1 (0.5%)
*Culex quinquefasciatus*	61 (30.8%)	N/A	N/A
*Anopheles gambiae*	59 (29.8%)	5 (2.5%)	N/A
*Drosophila melanogaster*	53 (26.8%)	2 (1.0%)	4 (2.0%)
*Apis mellifera*	50 (25.3%)	4 (2.0%)	3 (1.5%)
*Mus musculus*	39 (19.7%)	1 (0.5%)	0
*Homo sapiens*	42 (21.2%)	9 (4.6%)	1 (0.5%)

To determine whether these putative orthologs are known Sfps or sperm proteins in their respective species, we cross-referenced the putative orthologs to published lists (if available) of Sfps and sperm proteins. It is important to note that any relative percentages of orthology involving Sfps of other species should be interpreted cautiously. This is because the studies of Sfps in other species have used a variety of methods, some of which are more exhaustive than others. With that caveat, comparisons of *Ae. albopictus* Sfps with those reported in the other seven species demonstrate limited conservation in the complement of Sfps. Specifically, only 34 (17.2%) of the Sfps from *Ae. albopictus* have a putative ortholog to a reported Sfp in at least one other species (Table S1). Of those, 18 have a putative ortholog that was reported as a Sfp in *Ae. aegypti*. This was more than the number of Sfp orthologs in any of the other species in our comparison (Table 2), and was expected based on species relatedness and similarity in methodology. Notably, only four of the *Ae. albopictus* Sfps have a putative Sfp ortholog in more than one species (Table S1). Specifically, three Sfps (Aa-8246, Aa-24416, Aa-45626; see Table S1) have an ortholog to a Sfp in two other species. Information about the predicted protein classes to which two of them (Aa-8246; Aa-24416; a putative angiotensin-converting enzyme and a putative serpin) belong is included in Table 3; the third is a predicted aspartic/aspartate aminotransferase which, to our knowledge, have not been reported to have a direct role in post-mating responses or fertility. A fourth Sfp (Aa-14624, a predicted heat shock protein) has an ortholog to a Sfp in three other species, and is discussed in Table 3 and in the text below.

**Table 3:** Proposed role of selected *Ae. albopictus* seminal fluid protein classes in relation to reproductive biology.

Proposed general role relevant to reproduction or post-mating biology	Putative protein class	Examples of proposed specific role or involvement of protein class, based on previous studies	Phylum^a^	Species (if specified) in example	Evidence for example^b^
Sperm protection from oxidative stress					
	Adipokinetic hormone (AKH)	Targets hydrogen peroxide [58], [59]	A	*Pyrrhocoris apterus*	C (D)
	Glutathione S-transferase	Targets hydroperoxides [61]; see references in [63]	A	*Trichoplusia ni*	C (P)
		Responses to oxidative stress in idiopathic infertility patients [117]	C	*Homo sapiens*	C (P)
	C-type lectins	Sperm binding to egg [118], [119]	C, E	*Mus musculus, Strongylocentrotus purpuratus*	C (P)
		Maintenance and release of sperm in female storage organs [21], [120]	A	*Drosophila melanogaster*	CS (D)
Spermatogenesis and sperm-egg interactions					
	EHD1	Male reproductive success, via fertility and spermatogenesis [121]	C	*M. musculus*	O (D)
	Heat shock proteins	Sperm-egg interactions and recognition [122]	C	*M. musculus*	C (P)
		Spermatozoa ability to fertilize oocytes [123]	C	*M. musculus*	C (D)
	Puromycin-sensitive aminopeptidases	Male reproductive success, via copulatory behavior, spermatogenesis, and fertility [124]	C	*M. musculus*	C (D)
	Tetraspanin	Required (on oocytes) for sperm-egg binding and fertilization [66]–[68]	C	*M. musculus*	C (D)
Egg production					
	Adipokinetic hormone	Mobilizes release of carbohydrates and lipids from the fat body into the hemolymph (reviewed by [88]; see also [89])	A	*Locusta migratoria, An. gambiae*	C (D)
		AKH-like peptide promotes egg laying [93]	N	*Caenorhabditis elegans*	C (D)
	Angiotensin-converting enzyme (ACE)	Reproductive processes, via activating/deactivating neuropeptides and hormones (reviewed in [125], [126])	A, C		C (D)
		Egg production in females, from ACE in females [127]	A	*Anopheles stephensi, An. gambiae*	C (D)
		Egg production in females, from male-derived ACE (see references in [126]; [128]	A	*An. stephensi, An. gambiae, Tribolium castaneum*	CS (D)
		ACE activity or expression increases following a blood meal; peaks 48 hours post-blood meal [129], [130]	A	*An. stephensi, An. gambiae*	C (D)
	Aquaporin	Female fecundity, via altering production of neuropeptides [131]	A	*D. melanogaster*	O (D)
	Furin protease	Proteolysis of propeptide precursors of adipokinetic hormone [97]	A	*D. melanogaster*	C (D)
	Lipase	Egg production in females, from male-derived lipase [132]	A	*D. melanogaster*	CS (D)
Feeding and/or host-seeking					
	Adipokinetic hormone	AKH receptor in fat body involved in regulation of feeding frequency and consumption [94]	A	*Gryllus bimaculatus*	C (D)
	Fatty acid synthase	Activity associated with sucrose conditions via a transcription factor involved in fat storage and feeding behavior [98]	A	*D. melanogaster*	O (D)
		Upregulated in early diapause, during transition from blood to sugar feeding [99]	A	*Culex pipiens*	C (D)
	Heat shock proteins	Nutrient assimilation in midgut and triglyceride levels in fat body [101]	A	*D. melanogaster*	OS (D)
		Protein digestion and protection from temperature increase following a blood meal [133]	A	*Ae. aegypti*	C (D)
	Kinase	Control of feeding behavior [134], [135]	A	*D. melanogaster, Apis mellifera*	C (D)
Immunity					
	C-type lectins	Innate immunity in response to gram-negative bacteria [136]; see also [137]	A	*An. gambiae*	C (D)
	Ficolin	Innate immunity (reviewed in [138])	C		C (P)
	Serpins	Regulate innate immune responses (reviewed in [54])	A	*Ae. aegypti, An. gambiae, Cx. quinquefasciatus*	C (D)
Other: diverse roles of interest					
	Cathepsin D	Processes prohormones in mated females (see [100])	A	*D. melanogaster*	C (P)
	Metalloproteinase	Processes other Sfps inside the female reproductive tract [139]	A	*D. melanogaster*	C (D)
	Serpins	Responsive to courtship - expression in males decreases during courtship [140]	A	*D. melanogaster*	O (D)
	Heat shock proteins	Production of proteins in male accessory glands [133]	A	*T. castaneum*	CS (D)

a Phylum of species in example. A  =  Arthropoda; C  =  Chordata; E  =  Echinodermata; N  =  Nematoda.

b Protein used in evidence for example (C  =  protein is in same general protein class as *Ae. albopictus* Sfp, but not orthologous; O  =  protein is a putative ortholog to *Ae. albopictus* Sfp; S  =  protein is a reported Sfp in its species), and type of evidence for example (P  =  proposed; D  =  demonstrated).

### Seminal fluid proteins: Functional categories and proteins of interest

The 198 putative Sfps together have a variety of predicted functions (Figure 1D), and likely play diverse roles that are relevant to the reproductive biology of *Ae. albopictus.* In Table 3, we propose several possible reproductive and post-mating related roles of selected *Ae. albopictus* Sfp protein classes based on the demonstrated or predicted roles of putative orthologs or protein classes.

In the following sections we limit our discussion to potential intriguing roles of selected *Ae. albopictus* Sfps in relation to two facets of reproductive biology of this species: processes affecting fertilization (sperm protection and function) and processes affecting fecundity (egg development and feeding behavior).

#### Sperm protection and function

The reproductive success of males and females hinges on the ability of sperm to fertilize eggs. Here we highlight three *Ae. albopictus* Sfps that we predict are involved in sperm protection or function: a putative adipokinetic hormone, a putative glutathione S-transferase, and a putative tetraspanin.

Sperm are particularly susceptible to oxidative stress, which can damage the paternal genome [55], [56]. At least two Sfps we identified might play a role in counteracting oxidative stress. One is a putative adipokinetic hormone (Aa-134956; AKH). This Sfp has a putative ortholog in the full genome of all three mosquito species we queried and in *D. melanogaster*, but was not a reported Sfp in any of these species (although it was weakly detected in the seminal fluid proteome of *Ae. aegypti* [57] *;* Table S1). AKH can function as a stress responsive protective hormone [58] and can combat oxidative stress by counteracting damage from hydrogen peroxide [59]. It is interesting to note that the *D. melanogaster* Sfp known as sex peptide (Acp70A) has a region with similarities to the adipokinetic hormone of *Locusta migratoria* [60].

The other Sfp whose sequence suggests a role in reducing oxidative stress is a putative glutathione S-transferase (Aa-23220). In insects, glutathione S-transferase has been implicated in combating oxidative stress by targeting hydroperoxides [61]–[63]. Aa-23220 has putative orthologs in the full proteomes of *Ae. aegypti* and *An. gambiae*, although these orthologs were not identified as Sfps for those species. Jedlička et al. [64] found a link between glutathione S-transferase and AKH, showing that topical application of AKH to the abdomens of the pea aphid *Acythosiphon pisum* led to an increase in gluthione S-transferase activity. It is therefore possible that these two Sfps interact and play protective roles for sperm in the reproductive tract of mated females.

Some *Ae. albopictus* Sfps might play roles in sperm function in mated females. Tetraspanins are proteins involved in cell to cell adhesion [65] and are important in mouse fertilization [66]–[68]. They have been reported in the transcriptome of male reproductive glands in the tick *Dermacentor variabilis* [69]. We found one putative tetraspanin (Aa-5809) among the Sfps of *Ae. albopictus*, and it has orthology to sequences in the full proteomes of the five insect species queried in our study. It is interesting to find tetraspanins in the seminal fluid of males, as the sperm-egg binding role of tetraspanins typically refers to their role in the plasma membranes of eggs [70]. For example, the tetraspanin CD9 in the mouse oocyte plasma membrane is required for sperm-egg binding and fusion [66]–[68]. To our knowledge, the role of tetraspanins in seminal fluid has not been elucidated. However, Sonenshine et al. [69] proposed that adhesion proteins in seminal fluid could play important roles (e.g., binding of Sfps to sperm in *D. melanogaster* [71], [72]; binding of sperm to the oviduct in bovine [73]). The finding of membrane proteins such as tetraspanins in seminal fluid may be explained if accessory gland cells (in whole or part) are transferred to females during mating, as occurs in *D. melanogaster* [74] and is hypothesized to occur in *Ae. aegypti* [75], [76]. Consistent with this idea, tetraspanins may influence the composition of exosomes (via recruiting selected proteins), as well as the identity of and interactions with target cells [77]. Tetraspanins are also prostrate products in humans [78].

#### Regulation of egg development and feeding behavior

In *Ae. albopictus* and *Ae. aegypti*, mating promotes egg development [15], [79], [80], and, in *Ae. aegypti*, mating increases blood meal size [81] and may decrease blood feeding frequency [82] and host seeking behavior [83]–[85]. This suggests that in these mosquitoes, mating acts like a switch that diverts resources toward egg production and may alter a female's motivation to feed. In *Aedes* mosquitoes, egg production and blood feeding behavior are linked in the process of vitellogenesis, during which females use amino acids from blood-derived proteins to synthesize yolk precursor proteins (YPPs) in the fat body, secrete those YPPs into the hemolymph, and use the YPPs to develop eggs (reviewed in [86], [87]). Here we highlight several *Ae. albopictus* Sfps that might play roles in egg production and feeding behavior. These Sfps include a putative adipokinetic hormone, proprotein convertase, fatty acid synthase, and heat shock protein.

We discussed above the potential role of the putative adipokinetic hormone (Aa-134956; AKH) in protecting sperm, but it additionally might play a role in promoting egg production. In insects, AKH can play many diverse roles [57], including mobilizing the release of carbohydrates and lipids from the fat body into the hemolymph (reviewed by [88]; see also [89]). It has been proposed that some of these nutrients might be used to develop oocytes during vitellogenesis if they are taken up by the ovary [90], [91]. An AKH receptor variant is expressed in the ovaries of *Ae. aegypti* [90] and in *An. gambiae* [92], further suggesting a potential role of AKH in egg production. The influence of AKH on egg production is likely complex [57], but an involvement of an AKH-like peptide in promoting egg laying has been demonstrated in the nematode *Caenorhabditis elegan*s [93]. If AKH influences nutrient level changes in the fat body and hemolymph, it might additionally influence feeding behavior. Consistent with that hypothesis, knockdown of an AKH receptor in the fat body of the cricket *Gryllus bimaculatus* resulted in decreased circulating sugar levels, and increased feeding frequency and overall food consumption [94].

The role of a putative AKH in the seminal fluid is an intriguing and novel question, as to our knowledge AKH has not been reported as a Sfp in any other species. However, this AKH has a putative ortholog in *Ae. aegypti* that was weakly detected as a Sfp in *Ae. aegypti* (Table S1). Kaufmann et al. [90] found an AKH expressed in the abdomen of male *Ae. aegypti*, and hypothesized that the AKH mobilizes resources for sperm motility since sperm require carbohydrates and lipids for energy [95]. Our results suggest that the finding of AKH in the abdomen of males can be explained if it plays a role in females after being transferred in the seminal fluid. We agree with Kaufmann et al. [90] that future studies should investigate the role of AKH in insect reproduction, and particularly in the mobilization of nutrients from the fat body to the ovaries.

AKH may have a potential relationship with another *Ae. albopictus* Sfp, Aa-22559. Aa-22559 is orthologous to *Ae. aegypti* Sfp AAEL017460, a predicted proprotein convertase subtilisin kexin type 4 (PCSK4). PCSK4s can activate precursors of hormones through processing [96]. Interestingly, in *D. melanogaster*, a proprotein convertase named amontillado is required for the processing of the propeptide precursors of AKH, and influences the amount of carbohydrates released into the hemolymph [97]. It is possible that the putative proprotein convertase in *Ae. albopictus* seminal fluid may be similarly involved in the processing of AKH precursor propeptides and circulating nutrient levels in females after mating. Additionally of interest, *Ae. aegypti* PCSK4 shares close sequence similarity with the *Ae. aegypti* protein that processes vitellogenin, AAEL003652 [96].

Two other *Ae. albopictus* Sfps may play roles related to nutrient storage in the fat body and feeding behavior. One of these Sfps is a putative fatty acid synthase (Aa-14663) that is orthologous with dFAS (CG3523-PA) in *D. melanogaster*. In *D. melanogaster*, a transcription factor called Mio promotes increased dFAS activity under high sucrose conditions [98]. Flies with knockdown of Mio in the fat body decreased feeding behavior and fat storage compared to control flies, suggesting that Mio acts as a sensor to regulate fat storage and feeding behavior at least partially through its influence on dFAS activity [98]. Additionally of interest, in the mosquito *Cx. pipiens*, a putative fatty acid synthase is upregulated in females entering diapause, during the time that they increase sugar feeding [99]. The other *Ae. albopictus* Sfp that might play a role in fat storage and feeding behavior is a putative heat shock protein (Aa-14624). Aa-14624 has a putative ortholog in the full proteome of each of the seven species we used for our comparisons, and its ortholog has been identified as a Sfp for three other species (*An. gambiae, D. melanogaster, and H. sapiens*). The ortholog in *D. melanogaster* is glycoprotein 93 (CG5520-PA; [100]), which has been shown to promote lipid storage in the fat body and suppress insulin signaling [101]. Insulin signaling is involved in the regulation of vitellogenesis in *Aedes* mosquitoes (reviewed in [101]). These findings highlight the potential for the involvement of seminal fluid-derived putative fatty acid synthase and putative heat shock protein in female nutrient usage after mating.

### Evolutionary analysis of Sfps in *Ae. albopictus* and *Ae. Aegypti*

In a wide range of taxa, Sfps have been reported to evolve rapidly on average [102]–[104], and to turn over quickly at the sequence level, with substantially different complements of Sfps in different species [22], [23], [105]–[110]. The latter trend appears to apply to *Aedes* mosquitoes as well - as described above, many of the Sfps identified in this study were found only in *Ae. albopictus*. Only 93 (47%) of the 198 *Ae. albopictus* Sfps share orthology with proteins from the seven other species included in our comparisons.

**Figure 2:**
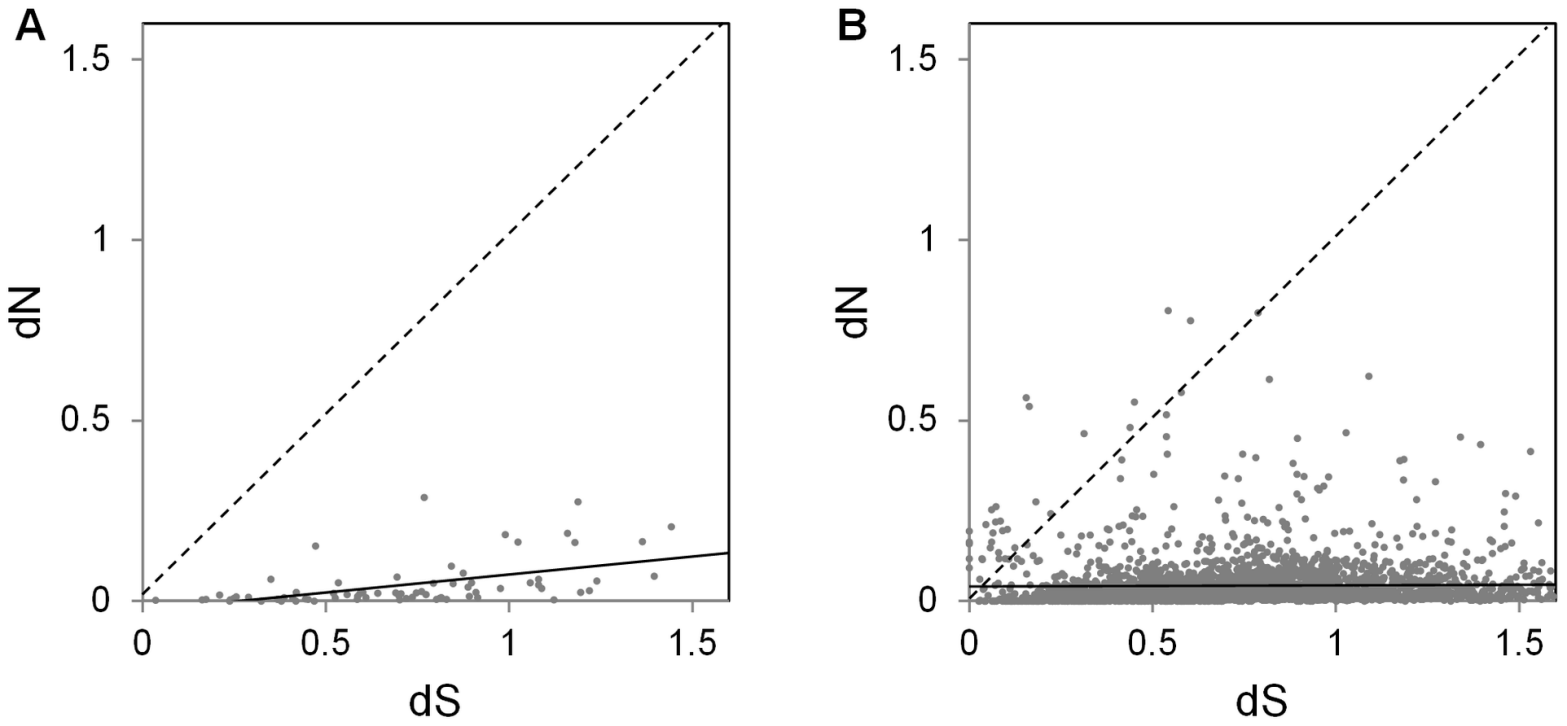
Estimation of evolutionary rates of *Aedes albopictus* seminal fluid proteins and control proteins. A. Evolutionary rates of nonsynonymous (dN) versus synonymous (dS) changes for *Ae. albopictus* Sfps (72 total); B. Evolutionary rates of nonsynonymous (dN) versus synonymous (dS) changes for *Ae. albopictus* control proteins (3495 total; 188 not shown due to extreme values of dN or dS). For both panels, the solid line shows a regression line, and the dashed line gives a 1∶1 dN/dS ratio, with points falling above the line showing dN/dS >1.

In order to assess whether *Ae. albopictus* Sfps also evolve rapidly at the sequence level, we estimated dN/dS for the 72 Sfps that have predicted orthologs in *Ae. aegypti*, recognizing that the most rapidly-evolving genes might not appear in this dataset as they might not have recognizable orthologs in *Ae. aegypti*. As a control, we used a set of 3498 transcripts (encoding putative housekeeping products) that were also identified in the current study (see Methods). The results are presented in Figure 2 (the raw data are available in Table S6 and Table S7). In contrast to results from other taxa, there was no difference in the rate of evolution between the shared Sfps and the control genes in the two species (Wilcoxon rank sum test W = 129390, *P* = 0.680). Moreover, none of the 72 *Ae. albopictus* Sfps that have orthologs in *Ae. aegypti* appear to have experienced positive selection (w = dN/dS >1) in this pairwise comparison. For both the control and Sfp genes, divergence should be sufficiently low to permit accurate estimation of dN and dS for most loci (median dS = 0.71 for both sets of genes; median dN = 0.020 for Sfps, 0.024 for control genes). Synonymous sites may approach saturation for a subset of genes, with the upper quartile of dS approaching 1 for both gene sets (Sfps: 0.91; controls: 0.97). However, the effect of synonymous site saturation should be to inflate estimates of w, and in this case we see very few cases where dN/dS >1. It is interesting to note that among those 72 *Ae. albopictus* Sfps having orthologs in *Ae. aegypti*, the Sfps derived from transcripts found exclusively in males had a higher average dN/dS than those derived from transcripts found in both males and females (Mann-Whitney U test U = 88.000, *P*<0.001).

Inference of positive selection from pairwise sequence alignments is inherently conservative. Since most sites in most proteins are likely under strong constraint, average dN/dS will be much less than 1, even if a few sites are subject to positive selection. Therefore, we used codeml (part of the PAML package; [51]) to infer site-specific selection on 46 *Ae. albopictus* Sfps for which orthologs were found in each of the other three mosquito species (*Ae. aegypti*, *Cx*. *quinquefasciatus*, *An. gambiae*). Consistent with the pairwise inferences of dN/dS, little evidence of positive selection was found using this approach (Table S8). Even before correcting for multiple testing, only one SFP showed any evidence for positive selection on a subset of codons, and even then only in the less stringent M8 vs. M7 comparison (Aa-18562; M8 vs. M7: *P* = 0.015, M8 vs. M8a: *P* = 0.165). While these four mosquito species are more distantly related than is typically used for the inference of positive selection, analyses using distant relatives can identify loci under selection [111], [112]. For example, positive selection has also been inferred for genes present in distantly related mammalian species [112], indicating that rapid sequence evolution can be inferred despite the requirement for orthology between distant relatives.

We note that these codeml analyses are limited to genes that are relatively conserved, since they are found in all four mosquito species. Nonetheless, the contrast with other taxa is striking. In *Drosophila*, for example, Sfps were initially characterized in *D. melanogaster* and/or *D. simulans*, with putative orthologs subsequently identified in additional species. As such, this set of genes was restricted to genes with orthologs in multiple species of *Drosophila*. Nonetheless, positive selection was inferred for six out of twenty-five *Drosophila* Sfps (at a false-discovery rate of 0.1) using codeml [109]. Thus, it appears that those Sfps that are detectable across mosquitoes are also constrained at the sequence level, recognizing that these mosquito Sfps are shared across genera, whereas the *Drosophila* Sfps are shared among species within that genus.

Both pairwise analyses within the genus *Aedes* and multiple-species inferences indicate that Sfp conservation differs between mosquitoes and *Drosophila*, but it is unclear what biological features underlie this difference. Rates of Sfp evolution may be driven in part by co-evolution of male and female proteins in response to conflict between male and female reproductive interests. This conflict is expected to be higher in polyandrous species, such as *Drosophila*, than in species (including these mosquitoes) in which females usually mate with only one male. Sequence constraint of this subset of Sfps suggests that alterations to their sequence are disadvantageous. Therefore, identifying and interfering with the pathways of these Sfps may prove beneficial for vector control.

### Conclusion

We identified in *Ae. albopictus* 314 male-derived proteins that are transferred to females during mating. To create a reference for identifying these proteins, we developed transcriptome sequence datasets of *Ae. albopictus* male reproductive tissues (seminal vesicles and accessory glands) and the female lower reproductive tract. The 198 seminal fluid proteins we report here represent a wide variety of functions (Figure 1D; Table 3; Table S1), and likely play important roles in aspects of *Ae. albopictus* reproductive biology possibly including sperm protection, sperm-egg binding, and egg production. Ninety-three (47%) of the *Ae. albopictus* Sfps we identified have putative orthologs to proteins in the full proteomes of other insects and mammals (Table 2; Table S1). However, only 34 (17.2%) of the *Ae. albopictus* Sfps have putative orthologs to Sfps in other species (Table 2; Table S1). On one hand, this suggests rapid evolution of the composition of seminal fluid in these species, although this finding should be treated with caution as identification of Sfps are limited by the sensitivity of the techniques used. On the other hand, for those *Ae. albopictus* Sfps for which orthologs can be detected in other species, there is little indication of positive selection on the Sfps in pairwise or multi-species comparisons (Figure 2). Further population-level studies, as well as comparative studies using more closely related species, will help to clarify the extent to which *Ae. albopictus* Sfps undergo positive selection.

This work contributes to our growing knowledge of Sfps in a diverse array of taxa, and establishes a foundation for several important lines of future research:

First, this work sets the stage for investigating the roles of individual *Ae. albopictus* Sfps on female post-mating changes in physiology and behavior [15]–[17], [80]. Much work in *D. melanogaster,* another Dipteran species, has elucidated the roles of specific Sfps on female post-mating behavior (reviewed by [21]), and recent work on *An. gambiae* has identified one Sfp that regulates semen coagulation and sperm storage [19]. Given the important vector status of *Ae. albopictus* and the potential for further disease risk due to its rapidly expanding range [5], elucidating the phenotypic effects of the Sfps in this species may assist researchers in identifying molecular targets for control [18]–[20].

Second, this work in combination with the identification of Sfps in *Ae. aegypti* [18], [36] might assist in pinpointing the molecular basis for ecological patterns of cross-mating dynamics in these two species. Studies have revealed a consistent asymmetric pattern in which the receipt of Sfps from *Ae. albopictus* induces typical post-mating changes in female *Ae. aegypti*, but the receipt of Sfps from *Ae. aegypti* has little to no effect on female *Ae. albopictus*. This pattern has been suggested with respect to several post mating behaviors including host-seeking [113], egg development and deposition [15], [79], [80], [113], and refractoriness to mating [114]. This asymmetry in Sfp cross-reactivity might create strong selective pressure for *Ae. aegypti* females to avoid mating with *Ae. albopictus* in regions where the two species coexist [115].

More generally, this work facilitates comparisons of Sfp components and functions across species. These comparisons can lead to the identification of sequences that are conserved and that promote male and female reproductive success. This work will also aid ongoing comparisons of Sfps across mosquito species that are vectors of disease pathogens, and ideally lead to the identification of novel targets for genetic-based control strategies that can be applied to multiple mosquito species [20]. Forty-six *Ae. albopictus* Sfps have putative orthologs in three other mosquito species (*Ae. aegypti*, *Cx*. *quinquefasciatus*, and *An. gambiae*) and showed no evidence for positive selection. These Sfps may be promising targets for use in genetic-based control strategies that modify and release male mosquitoes to induce reduced fertility or vector competence in their mates [116].

In conclusion, the work presented here provides a foundation for future investigations involving the molecular basis of multiple facets of *Ae. albopictus* biology, including reproductive biology, invasion ecology, hybridization and evolution, disease transmission dynamics, and vector control strategies.

## Supporting Information

Table S1 *Ae. albopictus* putative seminal fluid proteins transferred to females during mating (full functional annotation, putative tissue of origin, and putative orthologs in seven other species).
(XLSX)

Table S2 *Ae. albopictus* putative sperm proteins transferred to females during mating (full functional annotation, putative tissue of origin, and putative orthologs in seven other species).
(XLSX)

Table S3 Amino acid sequences of *Ae. albopictus* putative seminal fluid proteins.
(DOCX)

Table S4 Amino acid sequences of *Ae. albopictus* putative sperm proteins.
(DOCX)

Table S5 Amino acid sequences of *Ae. albopictus* proteins identified in the labeled virgin females.
(DOCX)

Table S6 Estimation of dN/dS for *Ae. albopictus* control genes with putative orthologs in *Ae. aegypti.*
(XLS)

Table S7 Estimation of dN/dS for *Ae. albopictus* putative seminal fluid proteins with putative orthologs in *Ae. aegypti.*
(XLS)

Table S8 Codeml analyses for *Ae. albopictus* putative seminal fluid proteins with putative orthologs in *Ae. aegypti*, *Cx. quinquefasciatus* and *An. gambiae*.
(XLSX)

Text S1 Description of methods used for transcriptome sequencing, assembly, and generation of *Ae. albopictus* reproductive tract predicted protein database.
(DOCX)

Text S2 Identification of additional putative *Ae. albopictus* seminal fluid proteins by searching mass spectra against the *Ae. aegypti* genome.
(DOCX)
