# Correction: Identification and Characterization of Seminal Fluid Proteins in the Asian Tiger Mosquito, *Aedes albopictus*

**DOI:** 10.1371/journal.pntd.0003073

**Published:** 2014-07-09

**Authors:** The PLOS Neglected Tropical Diseases Staff

## Notice of Republication

This article was republished on June 25, 2014 to correct an error in Table 1 that was introduced when the article was originally published online. In the original publication, Table 1 was only incorrect when viewing the article directly on the website. The original PDF download published contained the correct Table 1.
