# Supplementary material for: Identification and Characterization of Seminal Fluid Proteins in the Asian Tiger Mosquito, Aedes albopictus
Source: PLoS Negl Trop Dis. 2014 Jun 19;8(6):e2946. doi: 10.1371/journal.pntd.0002946 (PMC4063707; doi:10.1371/journal.pntd.0002946)
Supplement: Table S3 — Amino acid sequences of Ae. albopictus putative seminal fluid proteins. (DOCX) [file pntd.0002946.s003.docx]

| **Putative Sfp** | **Amino acid sequence** |
| --- | --- |
| Aa-10141 | ANLHVQGVESPALGTRQARNVHTLGDVDRAGQFVNILQRTLDTVEDAAHNTWTERMSGSAMYELVRVGYYELVGEIIRLEGDMATIQVYEETSGVTVGDPVLRTGKPLSVELGPGIMGSIFDGIQRPLKDINELTSSIYIPKGVNIPCLSRTQSWGFNPLNVKVGSHITGGDLYGLVHENTLVKHKLLVPPRAKGTVRYIAPPGNYTVDDIILETEFDGEINKWSMLQVWPVRQPRPVTEKLPANHPLLTGQRVLDSLFPCVQGGTTAIPGAFGCGKTVISQALSKYSNSDVIIYVGCGERGNEMSEVLRDFPELSVEIDGVTESIMKRTALVANTSNMPVAAREASIYTGITLSEYFRDMGYNVSMMADSTSRWAEALREISGRLAEMPADSGYPAYLGARLASFYERAGRVKCLGNPEREGSVSIVGAVSPPGGDFSDPVTSATLGIVQVFWGLDKKLAQRKHXXXINWLISYSKYMRALDDFYDKNFQEFVPLRTKVKEILQEEEDLSEIVQLVGKASLAETDKI |
| Aa-10382 | PHASYPGAAGSGFRATGGKKAAIRKKRKYELGRPAANTKIGASRVHFVRTRGGNRKFRALRLDAGNFAWASEGTARKARIIDVVYNASNNELVRTKTLVKNAIIVIDASPFRQWYESHYLLPLGKKREVKAGEEDVLAKKRSKRVMKKYVKRQKTAKLDPALEEQFNAGRLLAAIASRPGQSGRADGYILEGKELEFYLKKIKNKKSK |
| Aa-10393 | MTLAQSTTSAKPDDNHNSSESNDLDDQQQQQVIQLDFDVLPTQATETPPKIDSVVMHFSDDSDEQQQQQEDIQRPQTREELIRDLNADDSQQRNKRAPDSHSFEHGYFNRNPFFGSHSHESYEHHRHRPYGYNPYNNYNSYNPYNPYNSFDRYPYFNHHRGKRFVEMDLKVADQKETSEMEKMISEAIEMLDHMDADLFKRILLEVISVKRINQFSSAVEQKGDLYVARVVTANSHCIEEVEESAKCKALLIDGSNKFCTLEIRAKDKEVRLVKSECVPNKLLGGHREVDLSEPEHQQRIQAGLKGYAKGKLRSSRFIIRCGTVQVVAGTIHRYTVDLVDKDEQVTNTCNVKIYTPIQDAPEYTFDCQEVSRRVARDVERKKLSKGPKTGAPMEMTPEEFNKPEHAERIQSILLTAGGSNTERKYRIVEATQQIVAGSLFTYKLVFTDDPDKRVCTLTSHERPWLKEKSPSEARKVSFYCPEVSNSRPKRSFCAGCPTALSPNDLQDVEHKQRVNKILLDKVVGSQGELESLSPEIFNATSKVVQGMLYTYFVGFNMQGARRVCELTAWERPWLSESEAYKYTASCVEQEDSNAIRKRSKRSAFCVGCPTAITDLNGGEHKERVIKILLAKVAGSESQLKSMQPEIINATSQVVQGTMYTYFVAYRLEDSHQVCKLTSWERPWLDGAEAYQYTAECGAGQGDSNVLRKRRSKSRSKRAFGNPRVLTEEELQQDEHIKRVDAILASNPGVGAASPKIVNGTVQLVAGNSYTYYIAYTVNGEERVCQLNSWERPWLEEKQPEEAYKRTVKCDGTDESSVARARRHAKKTGASNELSAEDLKDKSHVERIKAGLVAYNTEKSKSYDDFEIVKGSVQLVAGSLYKYTFKVKSEPEVVCKISVWERVWLDTQDQRKYNVKCDGDDEPEQEQKLAASKRSARSVRPRQSDSDGEGHYSKGEDHARHLFEKFKLKHSRQYQSSLEHEMRFRIFKNNLFKIEQLNKYEQGTAKYGITHFADMTSAEYRQRTGLVVPRDDDRNHVGNPKAEIDENMEIPEAFDWRESGAVSPVKNQGNCGSCWAFSVVGNIEGLHQIKTKVLEEYSEQELLDCDAVDSACQGGYMDDAYKAIEKIGGLELESEYPYLAKKQKTCHYNSTKVHVRVKGAVDLPKNETAMAQYLVANGPVSIGLNA |
| Aa-11153 | MPKSMNVRVTTMDAELEFAIQQSTTGKQLFDQVVKTIGLAGGVVYFGLQYTDSKGDLTWIKLYKKTGSMPFSRWVMSQDVQKGDPLQFKFRAKFYPEDVAEELIQDITLRLFYLQVKNAILSDEIYCPPETSVLLASYAVQARHGDYNKTTHVPGFLVNDRLLPQRVIDQHKMSKDEWENSITTWWQEHRGMLREDAMMEYLKIAQDLEMYGVNYFEIRNKKGTELWLGVDALGLNIYEKDDRLTPKIGFPWSEIRNISFNDRKFIIKPIDKKAPDFVFFAPRVRINKRILALCMGNHELYMRRRKPDTIDVQQMKAQAREEKNAKQQEREKLQLALAARERAEKKQQEYEDRLRTMQEEMERSQANLIEAQEMIRRLEDQLKQLQFAKDELEARQNELQVMIKRLEESKNMEVAERQKLEDEIRAKQEEVQKIQEEVSVKDTETKRLQEEVEEARRKQVKRKNEAAAALLAATTTPNHHHVDEEEEDNEEELTNGAENGTSRDYSKDFDTDEHIKDPVEERRTLAERNERLHDQLKALKQDLALSRDDTMETANDKIHRENVRQGRDKYKTLREIRKGNTKRRVDQFENM |
| Aa-11858 | MSQVIMNGNGNHSTLANNHKEMHQRCLNPYRSKVTVVLGAQWGDEGKGKVVDMLATEADVVCRCQGGNNAGHTVVVNGKDFDFHLLPSGIINEKCTSIIGNGVVIHLPGLFDELAKNEAKGLTDWQSRLVISNRAHLVFDLHQQVDGMQEAEKGGKSLGTTKKGIGPCYSSKATRNGIRVSDLLGDFKVFSEKFESLVNMYKRLFPNFEVDVAAELARYRDYAERLRPMVRDTVSLVHASLKDGKSVLVEGANAAMLDIDFGTYPYVTSSNCSIGGVLTGLGLPPQTIGEVVGVVKAYTTRVGDGPFPTELHDEIGSLLQTRGGEIGVTTKRVRRCGWLDLALLRYTGMVNGYTSICLTKLDILDTLKEIKVAVSYNLRGEKIDYFPGSVTDLAQVEVNYIAMPGWLKSTENVRDFNELPPEAQDYVRMIENELGVPVKWIGVGKGRESIINVEE |
| Aa-12022 | RSISSSHVLTLLRFADDTYTESYISTIGVDFKIRTIDLDGKTIKLQIWDTAGQERFRTITSSYYRGAHGIIVVYDCTDQESFNNVKTWLEEIERYACENVNKLLVGNKCDLQTKKVVDTTTAMEYANQLGIPFLETSAKNATNVEQAFMTMAAEIKNRVGPPSSAAEAPSAVKIDKSRSVESKSGCC |
| Aa-12023 | MFCSCLTENWMTRVSSLLENGVNLAVLSVVALVAAEVYFEETFKDDSWEKNWVQSEHKGVEYGKFVRTAGKFFNDEENDKGLQTSQDARFYAMSAKFTPFSNKDDXXVIQFSVKHEQNIDCGGGYLKVFDCKVDQKDLHGDSPYLVMFGPDICGPGTKKVHVIFSYKGKNHLINKDIRCKDDVYTHFYTLVVKSDNTYEVLIDNEKVESGSLEEDWDFLPPKKIKDPEAKKPEDWDDRATIADPDDTKPEDWDKPEHIPDPDATKPDDWDDEMDGEWEPPMIDNPNYKGVWVHPEIDNPEYVEDKDLYLREEVCAVGLDLWQVKSGTIFDNFMITNDVEAAKKAAATFKDTQEGEKKIKEAQEAEERKKAEEEAKAAEADKDDEDLDDEEDEENTLGEATEVEDEGHDEL |
| Aa-121876 | MWFVHSNFNLAMSLVPYYSLLLIICALVGIKAQQSFDPCTCPCRKIIPVQVDPYLKTNANCVYIKSVYSKGLLFTSDVVIEGSRYVFHDVSHYKTAKPNYSHTAFWKIFYPWRNVSNTADVPLAVQNILSSEFMVVSHQIVEFTNRQIVTHPVLTIYSLWHFTYPGGYYKIMNQFTKEYLFSDEQHKSGWNEGKVFTDTIKRSSSDDFPGKYGFHITPCYE |
| Aa-121928 | MLHLVGLLLLAQAVAISCLPCSATTAAVRRVIVDQDGGGDDAWALLMLLMNEKQYNVKVEAITCADGNTDLENSVRNAARILDGIGRRDVPLYRGASERLITPAPSRDVNGYFWGHNGFGDVRFGSEPDLRNIPDAHAVVKMYELIRKYPGQITILCLGPLTNLAMLLKMFPKVKGDIAGIYILGGNRNGVGNTDFAAEFNFFTDPEAANIVVNNAPVILNIFPWETVLQLETDFPMDWRNEVFKVPRNKAIQVLNDVEAVVYANISAWQPCDMYAAAIFLYDNLRLVSKL |
| Aa-12606 | TLLYHIRVRSVPSRPLHKNLILAEVPNILFIQILVRIVVATLAQLFVRSAEPGDGRLELLRTVLALGVALSTVVLLQLFHVHGHLVAVDRSFIRRVRWVFSLRFQEALVVDVQDVHNEFVQQGYEIGTSGEGSRAVLLHRYDLRDEDDDDDSGNSTSSSSGGGGGNSTSTDDEKEVPTRILTSYLAKILLRIVVPTAALTACTDFVSLLNKLIVDILDINDKCFLEPQAKYPPDSADEGSIDCDEMTVYMKQLKKYYGGQCDSKGKNCPEKLKSAVSWFSRAYKQLGKSCYYDAYEYLDK |
| Aa-12657 | PESQTWKSQHNEHNNFQIHFDWLPSMFGFRSSESYESSESSQSSESSESSEFSKSLRSSKSSKETRVESRTCTGPNERYLRPWLMCDLDCRSWPGRLQSVPRIDLRCPSPCHCEKGYIRKEKGGPCIPIMECRYVRTAIEQLKVMIHGI |
| Aa-12663 | GRQPVKMNLKIIAFIVLALPCLAFRSSESYESSESSQSSESSESVEFSKSLRSSKSSKETRVESKTCTGPNERYLRPWLMCDLDCRSWPGRLQSVPRIDLRCPNPCHCEKGYIRKEKGGPCIPIMECRYVRTAIEQLKVMIHGI |
| Aa-12697 | FSSSMTAFDGKIYQRAFGGQSLKYGKGGQAHRCCCVADRTGHSLLHTLYGQSLSYDCNYFIEYFALDLLMENGQCVGVIALNLEDGSIHRFRSKNTVLATGGYGRAYFSCTSAHTCTGDGTAMVARAGLPSEDLEFVQFHPTGIYGAGCLITEGCRGEGGYLINSQGERFMERYAPVAKDLASRDVVSRSMTIEIREGRGCGPEKDHVYLQLHHLPPEQLAQRLPGISETAMIFAGVDVTREPIPVLPTVHYNMGGVPTNYKGQVLTVDSSGNDHVVPGLYACGESACSSVHGANRLGANSLLDLVVFGRACAKTIAAENRPGEKIADIKPNAGEASVANLDWVRNANGAVPTSTLRLNMQKTMQTHAAVFREEKTLQEGVRKMADIYKTIKDVKVSDRSLVWNSDLVETLELQNLLLNANMTITAAENRKESRGAHAREDYKHRVDEYDYSKPLEGQQKKPVEEHWRKHTLTWIDPXXXAVKIDYRPVIDQTLSEECNTVPPAIRSY |
| Aa-12735 | KSSHGSTLESIWSSAFDAEWMHPGERQRLSCSLEATFIFLPRREPVRAIGDWEEPLSDSCIRSLNSVEPCDDFYNYACGRFLKETNIPDEKVSVNTFSVIGDRLQEQLRSLVSEDIHEDEATPFKLAKNMYKLCMNKTRIEEKGIKPLLEILDSLGGWPVLKGDSWNADSSWSWTKSVKDFRMKGYSTDYFFDFSVGTDLKNSTRRIIDTDQAALGISREYLVKGMDNAIVSAYYSYMVDMAVLLGADEERAKRELMDSLNFEIALANISLPNEKRRNATALYNPMTVKDFQHKFPYTDWVEYFNVILKDTGIEIDENEVIIVSVPTFMEQLGPLLQNTPKRTMANYVMWRISGFSSFFLTENLRKRQLQYSTALSGKQEQEPRWKECVDITSGSLPISVGALYIRKYFKEDSKRAALDMVNGIKSVFVDILKKVDWMDEITRKSALDKVDSMVTHIGYPDELMDNNKIADYYKDLKFQPEDNYLNTILYMNQFGTTKAFKKLRQAVNKTDWITHSRPAVVNAFYSSIENSIQFPAGILQGQFFSYERPKYMNYGAIGFVIGHEITHGFDDQGRQFDKNGNLVDWWQSDTKKAYLEKARCIIEQYGNYTEPNVKLNLNGINTQGENIADNGGIKEAYYAYKQWAEKHGPEPRLPGLDLTPEQMFWLSAAQTWCSVYRPETMKMRITTGVHSPGQFRVLGPMSNMVEFSKDFNCPVGSPMNPAQKCEVW |
| Aa-130234 | TVVTAYIADRRLLIYKYIGKQYRMNKRGVIVQVEAESALEMNNRDEKLNTIDENGGITEEVRDFEESRREYISTLKDLRKKYPQHDLEQLEIMAQEQLMNKGPKSRAFYRIQATRKMMGSGNIMRKISERAQSDLSEVKAELQRVDVDEAIEEDAICKVFFEPGHYTVMESVGSFDVRVVRRGDLSSAVTVDYETEDGSAEAGSDYIGKKGTLTFPPGVDEQRFQIEVIDDDVFEQDEHFYIRLSNVSEPATLSTPKVATVMILDDDHSGIFALASKDHELVESVGVYELKVQRYSGARGRVRVPYWTEDGTGKAGKDYEEQRGELIFENNEIEKTIQLVIIEEGSYEKDVLFYVNLGEPQQIGDDELTGLIEAAESKAPEDLTEEDKMALLGRPKAGDIVRAQLRIKESKEFKNTVDKLVQRANASILIGTSSWKEQFIEAITVSAGDDDGENEGEEEPSPSCSDYIMHFLTLFWKIIFAFIPPTDMAGGYLCFVISILCIGVVTAIIGDVASHFGCTLGIKDSVTAIIFVALGTSIPDTFASKVAAIQDKYADASVGNVTGSNAVNVFLGIGVAWTIAAVYHACHGRSFDVDPGTLAFSVTLFCTEAFVAIIVLVMRRNPKIGGELGGPRKPKYLTAVFFFSLWIIYLLMSSLEAYGIIKGF |
| Aa-130358 | FEISLIHIGKRTHYYLGQWLRNRYKDLIGETYSGDEIYVQSTEVDRVLMSALSNLAGLYPPSDRDLWLPNIHWQPIPVHQIPKPLDDVIAGTKSCPKFLKLKEEYLQSDVYQGYLKSIEPVLKYANSHSKMELVSAETIYDLYSCLDIEHEHGFELPEWTSKVYPEPLKSICGEMFRYSTNTTAMARLRAGPIIKEILTRFQQKVDNTLVPSRNLWMYSGHDISVVNLLNGLGLFKPHNPPFCGLCHGGIKDGHTRFSIMFRFSTKPATTSRS |
| Aa-13381 | HTPTTIAFRNGDRLIGEDAQTLGVRFPANNFGYLVDLLGKTIDNPMVDLYRKRFPYYDIVEDPTRKTVVFRNGDEQYTIEELIAQLLQVAKGYAEESTGQSITECVLVVPGFFGQAERQALVSAARLANLKVLQLINDYTAVGLNYGIFRRKEFNETAQYFVFYDMGAYKTTASVISYQLVKDKATKEILPVVQVLGVGYDRTLGGLEMQVRLRDYLGQQFNKMGKTKTDIFTNPRAMAKLFKEAGRLKNVLSANTEHYAQIEGLLDEQDFRLLVTRDQFEELCKDLFARITAPIDRALSLSGLTLDIVNQVVLFGGNTRVPKVQEILKAHIKQELAKNLNADEAACMGAVYRAADLATGFKVKKFVVKDAVLYPIQVVFGREGESGNTRFVRRTLFGAMNSYPQKKVITFNKHTDDFEFAVDYADLESVLSKDEIANLGSLNLTKVSLTEVAKKLNANKADNVESKGIKAHFVLDDSGLFSLANVELVLEKTVKEDDESTLQKLGNTISKLFSGDSGEDKPTSTEEPSKEDSSKEEQKEDEKKSDEDKSNNGTNAENSTVSGDGEGAKNKTEKPKIVTMKEEIPSRVEVLYLSPLDG |
| Aa-13398 | MFSWLKRETKTEEVVENVLGELKKIYKSKLLPLEEHYSFHDFHSPKLEDPDFDAKPMILLVGQYSTGKTTFIRYLLERDFPGIRIGPEPTTDRFIAVMYDDKEGMIPGNALVVDPKKQFRPLGKYGNAFLNRFQCSTVPSPVLRAISIVDTPGILSGEKQRVDRGYDFTGVLEWFAERVDRIILLFDAHKLDISDEFRRSIEALRGHDDKIRIVLNKADMIDHQQLMRVYGALMWSLGKVLQTPEVARVYIGSFWDQPLRYDVNRRLFEDEEQDLFRDLQSLPRNAALRKLNDLIKRARLAKVHAFIIAELRKDMPSVFGKDSKKKDLIKNLGQVYDRIQKEHQISPGDFPDIKKMQEVLANQDFSKFHSLKIPLLEVVDRMLATDIARLMNMIPQEEMTMVSEPLIKGGAFDGVEDVVSPFGYRKGEGIDAGCGEVDWICNRDRERTDPIFESLRPIDGKISGAAAKSELIKSKLPNNVLSKIWKLSDYDQDGFLDIEEFALAMHLINVKMDGNELPVSLPSHLVPPSKRNGVAE |
| Aa-13470 | MGLSVAGCVVAICLCFGVAFGSEVARYDNYRVYEVIPTSSSQLKLLNDLKQSSDSLIFLESGTSVGVKFNIVVAPHKLADFTEALHNEGIRARLLETNMQKSIDEEKQQMVSKRARGAFDFNNYYELEDIHAWLDKLANQYDQVELLEGGHSYENRSIKGVKVSYKSGNPGVFIEGGIHAREWISPATVAYILNELLTSTDPKVRNIAENFDWYMFPSVNPDGYVYTHKKDRLWRKTRTPYSGGCFGADPNRNWDFHWAEQGTSSRCQSETYGGPYAFSEVETKTLSEFVASLKGKIQAYISFHSYSQLLLFPYGHTHEHTPNHNDLDEIAKATVTSLAKRYGTKYKYGNIYDAIYPASGASVDWAYGTLDVKIAYTYELRPGSGSWNGFVLPPKQIVPTGEETLDSLVTLLEESQKRGYYENCKDC |
| Aa-134896 | IWHSCSQSSGMMSLYEFEHPFQWATWTTVPVYPVAPARRCWFCRKLKHMENRVWPSLIPPMTQAAKGGLCGLNSPNPFSKLTTLMSCPLYIQRFLLGTSVLSTFCWNRVRISLIIGHDISVVNLLNGLGLFKPHNPPFAACVMVELRMATRDSPYVSVFYKTSNDEPELLDIPNCGPRCPLERMFELYKDIIPEDWEQECQI |
| Aa-134898 | MWKVLAITTVLLYTIIVQSKKLVSVEDHTKDKLIFAHVVYRHGNRTPLVAYDTDPWKDQSNWARGWGEITNIGKRTHYYLGQWLRNRYKDLIGETYSGDEIYVQSTEVDRVLMSALSNLAGLYPPSDRDLWLPNIHWQPIPVHQIPKPLDDVIAGTKSCPKFLKLKEEYLQSDVYQGYLKSIEPVLKYANSHSKMELVSAETIYDLYSCLDIEHEHGFELPEWTSKVYPEPLKSICGEMFRYSTNTTAMARLRAGPIIKEILTRFQQKVDNTLVPSRNLWMYSGHDISVVNLLNGLGLFKPHNPPFAACVMVELRMATRDSPYVSVFYKTSNDEPELLDIPNCGPRCPLERMFELYKDIIPEDWEQECQI |
| Aa-134922 | TERYFVVRRNRNLRSGDEIYVQSTEVDRVLMSALSNLAGLYPPSDRDLWLPNIHWQPIPVHQIPKPLDDVIAGTKSCPKFLKLKEEYLQSDVYQGYLKSIEPVLKYANSHSKMELVSAETIYDLYSCLDIEHEHGFELPEWTSKVYPEPLKSICGEMFRYSTNTTAMARLRAGPIIKEILTRFQQKVDNTLVXRRNLWMYSGHDISVVNLLNGLGLFKPHNPPFAACVMVELRMATRDSPYVSVFLQNQQRRAGATGYTELWSTLPTGKN |
| Aa-134956 | MDLVKLFSMLLICAALIFVCDAQLTFTPSWGKRAANPLSMNMPGSFGVQDSCKTPVDSLMVIYRMIQTEAQKILECNQK |
| Aa-135020 | AVSATFPFGVPLRSVRPLFWPQQTQIRKEHLLEAIRHLVLAGDLRESEVLQKAFQKSQPWWWKPPTQRLSHAPLIMIPPVKLYGRKSKNAHPSAVPRTRGKTGKPGKVAKVDITSLPAKSQHKIKQVVDELARNLQKASKGGSAVVSPKVAQKMIVHAIAKVDKIVHNEPDSDLREKMYTEAPPVMDSREVDYVDLPERRASPIRKQKKQESSSEESGSHESHESHESHESHESGESESEEYDESEESGRGSLKTLMGQLSSSLNQLMGALNKVSKKGKKGQ |
| Aa-13680 | MSLADPVAFLKDFAAGGISAAISKTAVAPIERVKLLLQVQHISKQIAEADRYKGMVDCFIRIPKEQGIGAYWRGNMANVIRYFPTQALNFAFKDKYKQVFLGGVDKNTQFVRYFVGNLASGGMAGATSLCFVYPLDFARTRLAADVGKGSGEREFKGLGDCLGKIFKSDGIVGLYRGFGVSVQGIIIYRAAYFGFYDTARGMLPNPKTTPWYVSWAIAQVVTTVAGIVSYPFDTVRRRMMMQSGRAKTEIIYKSTVHCWATIAKQEGTGAFFKGAFSNVLRGTGGAFVLVLYDEIKKLL |
| Aa-13837 | MGTGGNVKVNLRSIHSHSKVLNNSCEELVQRTRGLLRGPRHTGSRIYLYDKFDSIGNISDSDEEYEREEPEEDEYIIVPCTPTVYPADPFDANEDAATLRKAMKGFGTDEKAIIEVLARRGIVQRLEIAQAFKTAYGKDLISDLKSELGGKFEDVILALMTPLPQFYAKELHDAISGIGTDEEAIIEILCTLSNYGIKTIAEFYEQLYGVSLESDLKGDTSGAFKRLCVSLVQGNRDENTGVDEGAAAADAQALFEAGEGQWGTDESIFNQILVTRSYQQLRAVFEAYENMAGHSVEDAIKREFSGAIEEGFKAIVRCVRSKVQYFAKRLHHSMAGLGTNDKTLIRIIVSRSEIDLGDIKEAFQEMYGKSLESWIKEDCEGDLGDLLATLASY |
| Aa-13905 | GESEANVRDIFDKARSASPCVLFFDELDSIAKSRGGNVGDAGGAADRVINQILTEMDGMGAKKNVFIIGATNRPDIIDPAILRPGRLDQLIYIPLPDDKSREAILKANLRKSPVAGDVDLTYVAKVTQGFSGADLTEICQRACKLAIRQAIEAEIRRERERTEGQSSAMDMDEDDPVPNITRAHFEEAMKFARRSVSDNDIRKYEMFAQTLQQSRGFGNNFQIXXXGQSGSSSQGQGSSQPTSNPADNGDDDLYS |
| Aa-140565 | GHIDLCNFSGFSSFALNSGNRTRNCILGFGTVQFNRWKPPAQRLSHAPLIMIPPVKLYGPKSKNAIPSAVPRPRGKTGKPGKVPKLDMTSLPAKSQHKIKQVVDELARNLQKASKGGGAVVSPKVAQKMIVHAIAKVDKIVHNDPDSDLREKMYTEAPPNLPEPRVSLIRKQESSSEESGSHESHESHESHESGESGESESEESEESRRGSLKTLMGELSSSLNQLMGALNKVAKKGKKGQ |
| Aa-140567 | VEHAIASVRVVSTTHGWDVTKCNPQLTFILLAAASCHTRCRDSFQQEYHFEAYRPLFWPQQTQIRFEVEHRMEAIRHLVLAGNLREDEVLQKAFQKSQPWWWKPPAQRLSHAPLIMIPPVKLYGPKPKNAHPSAVPRTQGKTGKPAKVPKVDMTSLPVQSQHKIKQVVDELARNLQKASKGGSAVVSPKVAQKMIVHAIAKVDKIVHNEPDSDLREKMYTEAPPNLPEPRVSLIRKQESSSEESGSHESHESHESHESHESNESSESGESESEESEESGRGSLKTLMGQLSSSLNQLMGALNKVAKKGKKGK |
| Aa-140679 | VPTALGTVRVKVPARGCKSRELRGGMLIANCECLKRNGLQHDCPRSECQGKTCCLTKPEATCCPSTYARRFRNVTMGNKSNPVCPKRCRVTTVGSGCNPCAIDCYSGGPCGPCGPCGPCGPCAPCGPCGPCGPSPCGPVCDPCCGPVCDPSCPPVCDPCACVPQGDCGPCGAPTPNCMPCYDPCTPSIPPPCGDMCAPYPTCGPC |
| Aa-14389 | MSSKDRIPIFPSRGAQMQMKARLAGAHKGHGLLKKKADALQMRFRMILSKIIETKTLMGEVMKEAAFSLAEAKFLSGDFNQVVLQNVTKAQIKIRTKKDNVAGVTLPVFESYQDGSDTYELTGLAKGGQQMQKLKKNYQSAVKLLVELASLQTSFVTLDEVIKITNRRVNAIEHVIIPRIDRTLAYIISELDELEREEFYRLKKIQDKKRIAKKKEEARKAALLQEGIDVRVHANLLDEGDDDILF |
| Aa-14551 | YGPSSSDLRIVGGFADKIENVPYTVSISKRGYGHLCGGSLITLKWILTAAHCLLDDGPGNLYVRAGTTYKNKGGILRRVQTTISHALYTTQKLDLDVGLVQLFRPFPANNNLISTVRLRAPSEIVPPGVECVISGWGDTKENDGPYQILKSAKVKTVSQMRCQQALFRQVITKNMICAGAQGQDACQGDSGGPMVCAGILTGVVSWGEGCATIGKPGVYTSVSELRPWIHTFTGL |
| Aa-14623 | MLWYVLQFVMLGIIVGVNFFFLIILGVRAGVLLDEFLRFRLFVLRDSQGLAHHPLDAVGKVGRFWQLESGPQGSGVEHQDGHILGHDVIGIGFHSTEQLAHQRMAGVDFEGLALVQVVLPLWVISLLVGIGQSQTFHVTGPAEHGRNQGTRRVGQTFRDQGLGDLVLQGDVVQPLQQRFEFGLQFVKFGLGFLGLVQDETFLGNVLELLAVELGQSGDGVLVNGFDQVQDFVATREQAFGEGSLLDLGTARSGNEVDVFLLGLHPGDVIGQTGVLLGALAVGRLEPEQLGQTGPIAGVFHDSQLDVGGEFLPELVVDSDDLPLNVSRETLQQHKLIKVIKKKLVRKALDMIKKLDKETYDKFWKEFSTNVKLGIMEDPSNRSRLAKLLRFQSSNSKSSKEYTSLADYVSRMKPKQEHIYFIAGSSRAEVEKTPFAERLLSRGYEVLYLVEAVDEYSISALPEFDGKKFQNIAKEGFVLNESEEAKAKFDELKTEFEPLLKWLNDVALKDKIAKALVSERLSNSPCALVASMFGWTGNMERLALANAHQKTDDPQRQYYLNQRKTLEINPRHPLMRELLRRVEADSDDVVAKDMAVLMFNTATLRSGFQLPETADFADSIERMMRQTLGVSQDEQPEPEEFVEEDTGADSKEDEEEEVNADDDAEHDEL |
| Aa-14624 | MKYLLLLIVGVFLFAGISTVRADDDADIEDLPVVEMNLGASKEGSRTDAEAVQREEEAIKLDGLNVAQMKELRDKSEKFTFQAEVNRMMKLIINSLYRNKEIFLRELISNASDALDKIRLLSLTEPGVLDSNSNLEIKIKADKDGKVLHIIDTGIGMTKQDLVNNLGTIAKSGTADFLSKMQETKTETQDVNDMIGQFGVGFYSAFLVADRVVVTTKHNDDVQYIWESDAASFSIVEDPRGNTLQRGSQISLHLKEEAQDFLEEDTVKQLIKKYSQFINFPIYMWTSKEVEEEVPVEEEVTEKPEKKDEDATEEEDGKVEEEAEDEKPKTKKIKKTVWDWEVMNDSKPIWTRKPNDVTDDEYTEFYKSLTKDTSDPLTHTHFVAEGEVTFKSLLFIPKVQPSESFNKYGTKSDNIKLYVRRVFITDEFNDMMPNYLNFIRGVVDSDDLPLNVSRETLQQHKLIKVIKKKLVRKALDMIKKLDKETYDKFWKEFSTNVKLGIMEDPSNRSRLAKLLRFQSSNSKSSKEYTSLADYVSRMKPKQEHIYFIAGSSRAEVEKTPFAERLLSRGYEVLYLVEAVDEYSISALPEFDGKKFQNIAKEGFILNESEEAKAKFDELKTEFEPLLKWLNDVALKDKIAKALVSERLSNSPCALVASMFGWTGNMERLALANAHQKTDDPQRQYYLNQRKTLEINP |
| Aa-14663 | EGEPTLVSDVAVVTNYQPDTLVQHFGTDSGVRVVAKDAAAGPIEQGCHMVISYDILGRPDSQAVLSYLRESIRDDGFLFLEESRTNFDATKKGKALFDSLKLTTVTYQFYDKKVFVLLRPTNSYEQRKTTVIQVTEKNFTWVETLKAALAKAEETNTFVYLVCQGTQGIGAQGFINCIKNEAGGKFARMIFIQDKRAEKFSLTGKSYVEQLKKDLICNVMNSSGVWGSFRHLRLDNQTNGTSLQVEHAYVNALTKGDLASLKWIEGPLSRDRPDPKDKKQELCTVYYAPINFRDVMLSSGKLGVDALPGDLPTQDCILGLEFSGRDASGRRIMAMVPAKSLATTCVAHRNMMWEIPDNWTMEQASTVPCVYSTVYYALVVRGRMKRGESILIHAGSGGVGQAAISVALAAGLTVFTTVGSKEKRDFLKRTFPQLQDRHIGNSRDSSFEQMVMRETQGRGVDLVLNSLADEKLQASVRCLGLNGRFLEIGKFDLSNNSPLGMSVFLKNTSFHGILVDSIMDGDDDVLAEVVRLVSEGIKSGAVRPLPTSVFGDQQVEQAFRFMASGKHIGKVVLKIREEEKAKVVVPAPKLISAIPRTYMHKEKSYILIGGLGGFGLELSNWLVSRGATKIVLTSRSGVRTGYQALMIKRWSERGVTVSIDTNDVTTLKGAQKLLQAANKLGPVGGVFNLAAVLRDGLLENATEADFKTVCVPKVDGTKNLDQATRELCPDLDYFVCFSSVSCGRGNIGQVNYGLANSAMERICEARQAVGLPGTAIQWGAIGDTGLVLENLGDNDTVVGGTLPQRMPSCLQTMDFFMQQPCPVLASMVVAEKRKAETGGAGLVSCVANILGLKDTKNVSDSATLADLGMDSLMGAEIKQTLERNFDTVLSAAEIRMLTFGRLKALESGGADVPGSPVPPTSASPEPKEQDQNGLGDGTQVRFSADLMPTQCLVRLDSKAPPSSKDRPVFMVHAIEGVITSLIPLAQTLPVPVYGLQCVAEAPIESLETLAAFYIKHVRSVQPKGPYTIVGYSFGASVAYEMVAQLENAKETCRLLMLDGSPRYIAGYTDAQKQRIDNGEALQAEDEAYALAFFAMVCGNLDYSKTAHELIAPKTWEARLQKCAEMVRVRTPQYSQKLLETTAESFVHKIVAGHLYKPTSKISAPVKLVKPTENYAKLQGDYGLSELCTKEVKVTTVKGDHRSILVGDSMLEISKLLHELL |
| Aa-15007 | MPEAAADVETFAFQAEIAQLMSLIINTFYSNKEIFLRELISNSSDALDKIRYESLTDPSKLDSGKELYIKLIPNKEAGTLTIIDTGIGMTKADLVNNLGTIAKSGTKAFMEALQAGADISMIGQFGVGFYSSYLVADKVVVTSKSNDDEQYVWESSAGGSFTVRQDAGEPLGRGTKIVLHIKEDQLEYLEESKIKAIVNKHSQFIGYPIKLLVEKEREKEVSDDEAEEKDEEKKEEDKKEDEPKIEDVEDDEEKKDKKKKTVKVKYTEDEELNKTKPIWTRNADDISQEEYGEFYKSLTNDWEDHLAVKHFSVEGQLDFRALLFVPRRMPFDLFENKKKKNNIKLYVRRVFIMDNCEELIPDYLNFIKGVVDSEDLPLNISREMLQQNKILKVIRKNLVKKCLELFEELAEDKETYKKFYDQFSKNLKLGIHEDTSNRQKLADLLRFNTSASGDEYCSLGDYVGRMKENQKHIYFITGESVEQVKNSAFVERVKKRGFEVIYMTEAIDEYVIQQLKEYQGKQLVSVTKEGLELPEDEEEKKKREEDKAKFENLCKVMKSVLDNKVEKVVVSNRLVDSPCCIVTSQYGWSANMERIMKAQALRDSSAMGYMAGKKHMEINPDHAIIETLRQRAEADKNDKAVKDLVILLFETALLSSXXXXXXXXVHASRIYRMVKLGLGIDEDEPVSAEESAPAAGGDAPPLVDDAEDASHMEEVD |
| Aa-150366 | HFLLLFTAQWPERAQSNTVVRLEDLPSPTCFTIKTFLHRYIVDSAKFWRNDPKKRRVFRYNTLSRYMPDAAMWEATTVDPQAHGIHLMNIRTTSFMYASSNADSLSASCRNVFLRSDARETQGEASRFLFHYNSTTQAYRIQNLATKEYLQNLPSAFQNNLAVVCLDRRGGVEQGAEENYDFWLNSCDDEYFENKKITAAINSNYGLM |
| Aa-150479 | MAESRGYSVVIFLLLSSFAAVVWTTDNMPADKDVSKLFPLTLIHINDLHARFDETNMKSNACTAKDQCIAGIARVYQKIQDLLKEYKSKNAIYLNAGDNFQGTLWYNLLRWQVTADFITKLKPTAMTLGNHEFDHTPKGLAPYLAELDKAGIPTLVANLVMNNDPDLKSSKIQKSIKVTVGGKTIGIIGVLYDKTHEIAQTGKVTLSNAVETVKREAAALKKDKVDIIVVLSHCSYDEDKKIAKEAGQDIDVIVGAHSHSFLYSKESNKPYDQKDKIEGPYPTIVESNNKRKIPIVQAKSFGKYVGRLTLYFDNEGEVKHWEGYPEFIDNKVKQDPKILEALIPWRKKVQEIGSTKVGETTIELDRDSCRDKECTLGVLYADAFADHYTNSSFRPFAIIQAGNFRNPIKVGKITNGDIIEAAPFGSTADLIRXXXXXXXXXXEHSFALDDENRTNCLQVSGLRIVIDPSKSVGSRVVKIDVMDNRNPKSEDLKPLDRNAEYFIALPSYLADGKDGFSAMKKATARWTGPLDSDVFKSYVEKIKKVDKLKLGRVIVCKAGSPCT |
| Aa-15254 | MTTYFNYPPKDVQEELARIAKAIVAPGKGILAADESTATCGKRFADIGVENNEDNRRQYRQLLFTADDRLQENISGVILFHETLYQKSDDGTPLAAMLKKKGILAGIKVDKGVVDLMGSEGECTTQGLDDLGARCAQYKKDGCDFAKWRCVLKIGKNTPSYQAILENANVLARYASVCQSQRIVPIVEPEILPDGDHDLERCQKVTETVLAAVYKALNDHHVFLEGTLLKPNMVTAGQSCAKKPSAQEIALATVLALRRTVPAAVPGVTFLSGGQSEEEASVNLNAINQVPLLRPWALTFSYGRALQASVLRAWGGKKENIKAAQDELIKRAKANGDSSLGKYGGGVVGAAGQGSLFVANHAY |
| Aa-15307 | MYRTVLPILLVVVLSSAADNGPLVLKDTDYWLERIKHPPRNETKHIIAPLIVTIPGSLDNKQAEQLVQNLVELLKIHLGDSMPKTNRTVIEQTGYGVFNNSDAVLKMLMKKQAERERARERKKEEEKVAAGIKKVGKLIDDAEKAAKKAEKAKKDKEKKGSNKKEPDWYVKKPRLPTYKPFELKKSHEHDRKKSSEEDNKDDSREDSKEHRKEYSREYSKEDSEENSKEYSKEDSKQDSDEDSKGDSKKHSKKYSKEDSEEDSKEHSEEDSKEKKEDSPNPIAGISKMLKEEAKKLKKLKPKKSSDEKLKEMVQQLTKSLTVLVDIIDEVIETXXXXXXXDVPSSVVDRVDDVIPDALIAADGIAKQIGKD |
| Aa-15322 | IFLFLILFGLFGFLSCLLRVVNQFPHLLYSCGDLLLFFLPFPRSFPFGLLLHEHLQHCIRIVEHSIARLFDHCVIGLGHAIPQVDLKKLHQVLNEPLSLFVVQGIKKVGKLIDDAEKAAKKAEKAKKDKEKKDSNKKEPDWYVKKPRLPTYKPFELKKSHEHDRKKSSEEDSKDDSREDSKEHRKEYSREYSKEDSEENSKEYSKEDSKEDSDEDSKGDSKKHSKKYSKEDSEEHSKEHSEEDSKEKKEDSPNPIAGISKMLKEEAKKLKKLKPKKSSDEKLKEMVQQLTKSLTVLVDIIDEVIETGDVPSSVVDRVDEVIPDALIAADGIAKQIGKD |
| Aa-15323 | EPRPAELAAPFGTFFPWRSSTTKKQAERDRARERKKDSNKKEPDWYVKKPRLPTYKPFELKKSHEHDRKKSSEEHSKEDSKEDSKSHEHDRKKSSEERSEEDSREYSKEHSKKYSREYSKETSEEHSEEHSKEDSKEDSKEEKKDSPNPIAGISKMLKEEAKKLKKLKPKKSSDEKLKEMVQQLTKSLTVLVDIIDEVIETGDVPSSVVDRVDDVIPDALIAADGIAKQIGKD |
| Aa-15332 | QGTQQGIQRGNIARKTDKEYSKEYSKEDSKKTATKIDKETARKIARNTARKTAKSTVKNTDKEHSKEDSKEKKEDSPNPIAGISKMLKEEAKKLKKLKPKKSSDEKLKEMVQQLTKSLTVLVDIIDEVIETGDVPSSVVDRVDEVIPDALIAADGIAKQIGKD |
| Aa-15336 | MLIKKQAERDRARERKKEEEKVAAGIKKVGKLIDDAEKAAKKAEKAKKDKEKKDSNKKEPDWYVKKPRLPTYKPFEMKKSHEHDRKKSSEEDSKDDSREDSKSHEHDRKQNSEEGSEADSREYSKEHSKEYSREYSKEDSEEHSEEHSKEHSKEHSKEHSKEDSKEKKEDSPNPIAGISKMLKEEAKKLKKLKPKKSSDEKLKEMVQQLTKSTSTVSDFVNCWSTSSMRSSRRVMFRRQWSIELMRSFQMR |
| Aa-15468 | FRCKSGDVLRSLSTDEEFQKLLDTTNRLHKKFPNFLAGFDLVGQEDPGRSLFEFAPALLKLPASINFFFHAGETNWYGMKTDQNLVDAVLLGTKRIGHGFAVLKHPKVLKEIKRRQICIEINPISNQVLKLVQDQRNHPAALLFSDNYPVVVSSDDPSFWRSTPLSHDFYVAFTGIASAKQDLRLLKQLALNSIEYSAMNSEEKTEAKEKWNKAWDHQISGLAVDIVAGKI |
| Aa-154956 | MKTTVGIVVLLALGALASPIDQRLSLIRDETGNLHLVNPDPYSVIEAELEPFFTPETDVIFRLFTRSNPVHGQILQWNNPASVSNSNFNPAHPTRFTIHGWNGGETSGLHGNIRQHYFTVGEFNVISVDWGAGANTINYITARNRVESVGDIMSRMINTMVSATGMSRNSISLIGHSLGAHAAGCAGKFQNGQIHTIVGLDPAGPLFSLGQSDIMAPSDAQYVEAVFSNAGLLGFDLPLGDANFYPNGGRSQPGCGIDLAGNCAHSRAHELFAESVSTTVGFWATRCASHAEVNAGNCTPSGASAAMGGEPSNHGRGVNGVFRFNTNSAFPFAMG |
| Aa-15563 | MKILLAVVFVLNLTNLAVPQHLITSSPSLPESKPVGRRPTYEEYKQQRESFLQTEDHHLLGANVTLTENEQLVNKFIMQMKLDEMEKGFNDSYNFIPARHIFEVLDRFGQSKVFNVIRRLPKGGVLHAHDMALGSTDLIVNATYLENLWQKGNFGLNNGPEFKFSRERPGKEWSLVSEIRQWMTNEVYDAKVAEVFSLYNADPLNAYKSLDNVWSKFQNLFACLAPLITYAPVWRQYYHDSLKQFYDDHVQYLEFRGVLPEVYDLDGKVYSAEEIVQLYYEETEQFKAKYPDFIGVKFIYAPGRYATDEEFQKLLDTTNRLHKKFPNFLAGFDLVGQEDPGRSLFEFAPALLKLPASINFFFHAGETNWYGMKTDQNLVDAVLLGTKRIGHGFAVLKHPKVLKEIKRRQICIEINPISNQVLKLVQDQRNHPAALLFSDNYPVVVSSDDPSFWRSTPLSHDFYVAFTGIVSAKQDLRLLKQLALNSIEYSAMNSEEKTVAKEKWNKAWDHQISGLAVDIVAGKI |
| Aa-15857 | MTKLYHRVSLLILLVSSVGYLDALDFAGLAKSTGDVAKVAGKTVKTVVEKVPEIFSPEQLADFAKQSIAGVPLEALAATINKICSFAVSANATESENSVNISALNYVLMTGSENVTVPLLDSDALWSYPLFNDSQDTVILVTGWTSNINGSNRAIDTIFSAYQARGGINFVVIDTSDFVDTLYTWSAFNTNELGEALAVGLQHLINFIPVEKIHLIGHSLGAHIVGSAGRHFQRLTNASIPRITGLDPANPCFNEGEALSGISRGDADFVDIIHSNAKVLGKRDPIGDVDFYPNGVVSVQPGCLDPSCSHARAWELYAETVYPGNENHLLAVKCNSILSLDTGACPGKAIPLGYACPRTAKGNYFLKTNDKFPFGKNL |
| Aa-159758 | MANGCPRRRSSSCRPGKTSRNPYINFLRDYRKKHCGLHPVEVIRQGACAWNRLSDQQRLPYIRTAFYRPIRREPCSSTRGRRSRSRGRSVSRSRSRSRSRRR |
| Aa-16385 | VVLFVRCYVSFCFHTVDSYAVEPVFTDGYSSVSAPFCQCPCENRISISKDWFNCITIYSMKTKGYVTADEHYPFFKEDRYVFATNQNKLWGTAKWKVEYREGNNGTYGLKNLYVKEWLHVGRNDQARDSFRRYLLTKIKGDPNIPPRDGYWQFIPDPKIGKDVYRIRNTFTGEYLFVDDEQHIQRYDYNRLYLWRHTGNYQSTDNRHWFKMNKSLFYAQNY |
| Aa-16482 | MKLIVIVVLALALSCLASDSSSEYDELEASLNIDKTCKHPHEVYDDCGSACEMTCENWQPEPLVCIRKCDPGCFCDSGHIRSNATGLCIPKGKCWTIVKAQS |
| Aa-16497 | MSEPIRVVVTGAAGQIAYSLLYMIAKGDVFGPNQRLVLHLLDIPPMMGVLEGVVMELADCALPLLAGVVPTTDPAVAFKDVVAAFLVGAMPRKQGMERKDLLSANVKIFKVQGEALNNHARKDVKVLVVGNPANTNALVCSHYAPSIPKENFTAMTRLDQNRAQAQIAARLGVGIAQVKNVIIWGNHSSTQFPDAKNATVEVDGTKKSVVDAIANNDYLNGEFVETVQKRGASVIAARKMSSAMSAAKAASDHMRDWFAGTKDGEYVSMGVVSDGSYGTPKDVVYSFPVQIQNGQWKIVQGLSVDDFARGKLDATGKELLEEKEEAMSVCASD |
| Aa-16724 | ERLPKDIIAACHNSSDSVTISGPVDSINKFVAELNAEGVFAKAVKSSGIAFHSRYIADAAPKLRKSLDKIIPNPKNRSPRWISTSIPEEAWNTPLAQQSSAAYHVNNLLSSVLFSEGLRHVPSNAICIEIAPHGLLQAILKRALGKDATNLSLMKRGHDNNVIFMLSNIGKLYAAGAQPQVQKLYRPITYPVGRGTPMLNSLVKWDHSTKWYLARFGVENKSGETVIDVNLEKPDDAYLAGHTIDGRVLFPATGYMTLAWRTFAKMRGSDMEKTPVVIENAVFHRATILPKDGSVKFGVNFFDGTGAFEICEGGSLAVSGKITVPENIDNEELPLYPIDEDKSGLSMSTSDSYKELRLRGYDYGGLFRGITKADASRVTGELQWKDNWVSFMDTMLQFSILGKNMRDLYLPTRIEKIVINPARHMDMMNTLKSKDQDVPVAVYRNIDVIKSGGVEMRGLKATLAPKRSGSQAPPTLEKYVFVPNFNEKELAESGEKSRFRSITR |
| Aa-16726 | AAIPSRGKRSKQKKTRRNKSATMPARFDDVTTDTRRGVPRDLGGHYDGCQIQDDICITGFSGRLPESSSIEEFKRNLMEGVDMVNDDDRRWPKGLYDLPTRIGKIKDEDLQNLDAEFFKIHQKQAECMDPQMRMLLECTYEAIIDAGINPQELRGSRTGVYIGCSNSETEQHWCADPDLVNGYGLIGCARAMFANRLSFTFDFKGPSYAVDTACSSSLIAMSTAFADMKAGRCDAAIVAGCGIILKPTMSLQFKRLNMLGKEGMCKVFDESGNGYVRSDGCVVTFMQRASDSRRIYASVLNVRINTDGYKEQGITFPNGAMQKRLIQETYGEINLNPADVVYVEAHGTGTKVGDPQEVNNITDFFCKDRKAPLLIGSVKSNMGHSEPASGVCSIAKMLIAMEAGIIPGNLHYKNTNPDLYGLMDGRVKVVDRNLPWNGGIIGLNSFGFGGANAHVIMKSYPKPKPVSPKDGFPKLVLASGRTEEAVEAFLDAANGNKDDEEFVGLVNEVHSKNIPLHYFRGYTVMGEGQAVREVSDLSDDKRPIWFIYSGMGSQWASMAKEMMQVEVFNNSIHRCAEALRPEGVDLIDILTKSDESRFDNILNSFISIAAVQVALTDVLNHLGIAPDGMVGHSVGELGCAYADGCFTPEQTVLAAYWRGRSILDTQLIPGLMAAVGLSWEQCKERLRRTSSQLVITAVTALRFPDRWIRSTNLSPN |
| Aa-16746 | MRVLRSLGLVLFVAGLALVASDAEVKEEDGVLVLTKDNFQSVVEGNEFVLVEFYAPWCGHCKALAPEYAKAAKALAEKNSNIKLGKVDATEEQELAEKHGVRGYPTLKFFRSGTPIEYTGGREKDTIISWLEKKTGPAAKELETVADAEEFLKENNVAVVGFFKDRESAECKAFLTTANAVDDYPFAVTSSEDVYAKYEAKCGSVVLFKHFDDGKAVFDGEYTEEALKKFVAAQALPLIVDFSHETAQKIFGGEIKNHLLFFISKEAGHMEYIESA |
| Aa-16750 | RTGSSRGTSWRLPWRFDVFHVASFLGDEEQQVILDFTSEDLLVGFFKDRESAECKAFLTTANAVDDYPFAVTSSEDVYAKYEAKCGSVVLFKHFDDGKAVFDGEYTEEALKKFVAAQALPLIVDFSHETAQKIFGGEIKNHLLFFISKEAGHMEYIEAAKEVAKKFREKILFVTIDADQEDHQRILEFFGMKKDEVPSMRIIHLEEDMAKYKPETNDLAAEKVEDFVSKFFEGKIKQHLLSQELPEDWDKNPVTVLVADKFDEVAMDSTKDVLVEFYAPWCGHCKQLVPIYDKLGEKYADDDTVVIAKMDATANELEHTKINSFPTIY |
| Aa-16751 | FLISPPKIFWAVSWLKSTISGRAWAATNFFRASSVYSPSKTAFPFAVTSSEDVYAKYEAKCGSVVLFKHFDDGKAVFDGEYTEEALKKFVAAQALPLIVDFSHETAQKIFGGEIKNHLLFFISKEAGHMEYIEAAKEVAKKFREKILFVTIDADQEDHQRILEFFGMKKDEVPSMRIIHLEEDMAKYKPETNDLAAEKVEDFVSKFFEGKIKQHLLSQELPEDWDKNPVTVLVADKFDEVAMDSTKDVLVEFYAPWCGHCKQLVPIYDKLGEKYADDDTVVIAKMDATANELEHTKINSFPTIYLYRKGDNQKVEYRGERTLEGFVNFLEGKTEEPEVEETEEEHKPEKDEL |
| Aa-16763 | MFSVKVIATVALVAVLGLQTAFAGEADVLDLTDSDFSTRVAETETTLVMFYAPWCGHCKKLKPEYAKAAELLRGEDPPIALAKVDCTEGGKDTCGKFSVSGYPTLKIFKNGEVSQEYNGPREASGIAKYMKSIVGPASKDLLTLEAFEAFLKVQETSVVGFFQKESDLKGVFLKYADSQRERLRFGHSTAQEVLDKQGETDAIYLFRAKQLANKFEPDFVKFEGKTKDELSTFVKENFHGLAGVRTRDTLNDFKNPLVVVYYAVDYVKNPKGTNYWRNRVLKVAKEFVGRVNFAVSAKDDFQHELNEYGYDYVGDXPLVLARDAKNQKFIMKDEFSAENLQAFATELEEGSLEPYVKSEPIPENNDAPVKVAVGKNFEEVVMNNGVDTLIEFYAPWCGHCKKLAPAYDELAAKLKDEEVAIVKMDATANDVPPTFDVRGFPTLFWLPKNAKTSPQRYEGGREADDFLQYIAKHATEELKGWDRKGNAKKTEL |
| Aa-167926 | MTTSASTLTLLIILLNWCKWATPTIEDARIVGGYVTRIENVPYTVSINERRIGHFCGGSLISLEWVLTAAHCLEGAVPDNLYVRAGSNYKNKDGILQRVKAIISHTLYDKYITLDFDIGLVKLLEPLPAHNNYISTIRLASPYEFVPPGSYCLVSGWGDTRQNVSEYQILKSAIVQTVNRITCQRVLFRQPITKNMLCAGAHGHDACQGDSGGPMVCFGSLAGVVSWGDSCGTIGAPGVYTSVRALRFWIYTVTGI |
| Aa-17313 | MASGVTVSDVCKTTYEEIKKNKNNRYVIFYIRDEKQIDVEVIGDRNAEYDQFLEDIQKGGPGECRYGLFDFEYMHQCQGTSESSKKQKLFLMSWCPDTAKVKKKMLYSSSFDALKKSLVGVQKYIQATDLSEASREAVEEKLRATDRQ |
| Aa-18562 | MELKIANNAPKKRSSDKETGCELGIDERTGRLQWCPGYRFAKVLFVIPAAMLPLALLFLVLTRLHVVGSVNHSTLKRHDDHDLRYYADHSNKLDDLQCKPVRSSLSEVLEAEERDILKDVRTSFVPSSSVIHCAPDDRYDDLHSIPDDVGRSDIVNFFRAKRFSNRQRKAKKSVLQKIAWSNDGSAESIRSAQVEIMKKYMDLKTNPCDDFYQYACGNWDRVNPIPKDKAGLDTFEMLRESLDSVLKHLLLETSDGSLLEIENSLGTTLNPLETEHLRRVRKRIISKRDTLNKLVRDAQIRKTRRELASHVALENAVENAETKARHLFISCMNYSLIEQRGLDPLFKLIDSLGGWPVLNPNWDEEGFDWLNLTAQIRKFNNDILIVEWVGPDIKNSDENIIQFDQTSLGLPTRDYFLQPSNKKYLDGYKQFMIDVIQLLGVQPEVANVAANEMLDFEIQLANITSSVEERNNVSVLYRKVILENLHEEIPEIDWTRYLSIVMEKPINSSEFVVMFAVNYMKDLVLLINDTEPRTVANYILWRFVRHRINNLDDRFLQAKQKFSNVLFGREKSPPRWKNCVNQVNANMGMAVGAMFVRKYFDENSKRDTLAMTHELQQAFREILNETEWLDSPTKHLAEMKVNAMSLRIGYPDFILSHKDLNEKYADLEIHPEKYFENTLNVLSHIRRTDQNKIGQTVNKTAWHTAPAVVNAYYSRNKNQIMFPAGILQPPFYHRYFPKSMNYGGIGVVIGHELTHGFDDKGRLFDRDGNLYRWWSDHAIEAFHERATCLVQQYGKYTIDEIGVQIDGENTQGENIADNGGIKQAFRAYTKWLSEQSDPRVLEQETFPELNVTSAQLFFLNFAQVWCGAMRPEATRNKLKTAVHSPGRFRVIGTLSNSEDFAREYGCPVGSAMNPADKCSVW |
| Aa-19306 | MIKSVERAQMLKQLSRLLLASNGAASQMHNMPPKCRVVAGMQKRLSSSFANPNVKMSSTATATKVKQQNATASQEVFDREDKFGAHNYHPLPVALARGEGVYVWDVEGKRYYDFLSAYSAVNQGHCHPKIVQALTEQAKVLALTSRAFYSNVLGEYEEFVTDLFGYDKVLPMNTGVEGGETACKLARKWAYKVKKVPENKAKIIFAEGNFWGRTLAAVSSSNDPSSYEGFGPFMPGFELVPYNDTVALEKAFQDPNVCAFMVEPIQGEAGVVVPDEGYLKKVRELCTKYNVLFIADEVQTGLARTGRMLAIDHEDVKPDILILGKALSGGLYPVSAVLANDDVMLCIKPGEHGSTYGGNPLGCKVAMAALKVLIDEKLAENADRMGRKLRKSLSELPQDVVSVVRGKGLLNAIVINSKFDAWEVCLRLKENGLIAKPTHGDIIRFAPPLTINEEQMDDCLRIIKTTIMSFANKQ |
| Aa-193700 | MNTLKRVNPSRLVLQIRSKHTLPELPYDYSALEPTICREIMELHHQKHHAGYVEAFNSAEEQLREALVNCNPNKVIQLGKILRFNGGGHINHSLFWENLSPHCTAPSKALESGLKQNFYSKEDFKQLMCDTALSVQGSGWIWLGWHKPSKTMKIAACCNQDPLEATTGLLPLLGIDLWEHAYYIQYKNDRAKYFDALWEIIDWKMVSKRYEKLRCLIEK |
| Aa-195203 | FEDEAGLVSTNADFPSFCHFFNKMKHAMLLSLLALACTVTPALSYFPEEKGILELNPSNYVAAVRQFPYLMVEFYAPWCPYCQTFAPKYEDAALRLTTTNSPAKLAKLDASRYATFAAQLKVQEYPTMYFYRQGHPLLYNGQMEIVPWFDGFN |
| Aa-19705 | MTAQANSAEPQTSRTVMIETNPAVKISSEADSIGCVEVLATVCSTILMVLTLPISIFLCFKVVQEYERAVIFRLGRLRSGGARGPGVFFVLPCIDNYCKVDLRTVSFDVPPQEVLTRDSVTVSVDAVVYYRIRDPLNAVVQVANYSHSTRLLAATTLRNVLGTRNLSELLTEREAISHSMQVTLDEATDPWGVQVERVEIKDVSLPDSLQRSMAAEAEAAREARAKVIAAEGEMKSSRALKEASDIMCESPAALQLRYLQTLSSIAGEKNSTIVFPLPIELIGPLMNITSSITSKMAAASSTATTTAVADPRPSEVID |
| Aa-20439 | FSLEQWRSSTSFSTTTEALEQPKEHDEDMSPFLPNIESSSAKTRGSYAVTETILETETTPTLLTHVDQSPFLPEIEQNASLVKFLHEGLDRQVDGFDNDSQQQYLEVLPLSKESDRNEYKKQLDTVTKTVFVATTIRTYAPTTDSLEDLITAASSRNIVEQTTNSGGEEELMATSTEKDVEDTTLGKMGAVATTTERESARLEQSTEHVEEKPTTTAENETTNSSPKAEVEEIGTTESEQAEKATTVVMDDSKDDTPKGDVELVATNDTAEKLTATEAEEPTTEKHVMETTMGETKLELEEERVTASKDETEEVTDAVREDTTEKQAKALVEATSHKDVEETTVHAVVNASSTTTEPSGNAKLMEESTVETKEPPTTASPTTTPTATSDLTTTVAATTTEATSTTPEATTTTVVATSTTETATTPTTPTTTDRTKTDFLERTLQDLDQDNDISENDLKVLPLDRKPDKFEESPPPPDPLASTQKTTVDQESNFETTTGHFLGETTFHLIRSERSNETSLIKSRNSDDDDLSELVASASGMFTKCAVGQFECVNGTSIKDGSSCIQKSERCDSVSHCSDNSDEQDCERLGCPGHFQCKDGFCLARQHVCDGITHCNDGSDEVECDQLQCNFDEISCDSSGRKGPCLPAQWKCDGLEQCANGFDESDCPDTCTNDQYFCVGQRKCIPEAWRCDGKSDCTNDEDERLCDCPVDNFRCNTGGCVPSAYVCDGQPQCPDLSDEWGCFSLEDSKLKIRVESEKLNPVCADNWSKELSDAVCAELGYLQAKSYSISNETGGDAFYRFVGANSTNLLRSLALATSCSEGLVNIDCELYRCGSDRITPISDQRIAGGLHTDSNQLPSLALVYSNNAAIKCTANXXXXXXXXXXXXXXXXXXXXXXSPRWALASYSCMMGKTEFIDNRNIAEMDWKLFAGTSQFNFTMIRGNNANSSYQVVDVLRIVPYP |
| Aa-20752 | MITQSIVLVLILAISTCSIYADYCDPAICKNVKAQHVACNAPQKFGTPCGKDAKYITMDNKLKTIILNKHNELRAEIARGMYGFPQAARMPTLAWHDELAKVSTYNARNCTLEHDKCRNTKEFRYAGQNLGIIWYRRYNYQPEDRVTDFIQQWFDEHKDCPKSYIDKFPAKHDGPLIGHFTQMVNDRVTMVGCSLVHYVTYPNGEETLNYYFVCNYSMANVIGGRVYTKGTTGSNCKTGQNPNFKGLCSSKEKVEPKP |
| Aa-20813 | AAKRIDPRRLCHLLQSIFVSHFYSYESLLDNLPHNVLWSDCTQQYIPYITYKKNWTDAIEHCKCYGMRLAVVDTAAKQRLLEQALIGSAIFNDSWTSVWIAANDRAVEGQFVWQPTGKKVQYTNWRSLMPDNYQGYEDCVHVFRGIGFNFKWNDWPCTWLTQVACEEVKKC |
| Aa-20904 | MSEYWLISAPGDKTCQQTWETMNNLTRHQNNLCENFKFHIPDLKVGTLDQLVGLSDDLGKLDAYVEQSTRKIAAYLGDVLEDQRDKLYENLQANNNDLTTYITRFQWDLAKYPTKQSLRNIADIISKQVGQIDADLKTKSAAYNNLKGNLQNLEKKQTGSLLTRNLADLVNLFRDDVGNVAERLLRWVLGQIPLER |
| Aa-20951 | DTEVVVGCPAPYLTLARSQLPDSVGVAAQNCYKVAKGAFTGEISPAMLKDLNIGWVILGHSERRAIFGESDELIADKVVHALAEGLKVIACIGETLQEREAGQTEAVCFRQTKAIADKVKDWSNVVIAYEPVWAIGTGKTATPEQAQEVHAALRKWFTDNVSADVAGAIRIQYGGSVTAANCRELAAKPDIDGFLVGGASLKPEFIQIVNARQ |
| Aa-21004 | MNIQRLVHSILVVFCVLSCVSSKPYDGYKLYEVPISTGHEVQLLLYLQHHRPSIDIWALNKLGPKRMLVPANLQKTVEHFFKDQHINYTVKVEDFGKKYSVRQAPLMQLEDPTFLNSYPTYDEINDYLVQLATENFEWIRFRIIGWSVEGRPIRAITINPDKQRTIIVDAGIHAREWITVSTALYLIKKLIEDSDQYRILHEYKWVIVPLVNPDGYIYSMETDRYWRKNRRRLSDKCVGVDLNRNFGFRWDVGASLFSGECHPGFRGYAPFSEPESRALRTVFDNNPEAELYINLHSFGGYLIYPWSYESTPVANVGDFKQVGVAAARAMWHYSHQEYKVGSSAEILRYQASGTSIDYAYSVGIDYPFAMEIAEFGYSDFQPPTNAIADIVKESFVGIKELVYAVRKLKTQQKG |
| Aa-21108 | METATATSVKDYKKVGVDLKTQLESFNTENLSKADTQEKIVLPTAEDVQTEKSQKSLFAGIESFDATKLKHAETCEKNPLPDKEAIQQEKGQKNFINCIESFDTSKLKHTETHEKNPLPTKETIEEEKKA |
| Aa-21165 | GKPIPLGFVLGGIGLSVHEMFSQFVVQFLFLIFAPLSIDADGYLKWDGQCGGEKQPSQWYPRPVALHVGNECYPFKPDDGSSDIPIFPDRNDMCCACPNKPSRPPAWPPGVHEVTPPPKLTLTPKPGVGTTDKPIPPRTKPSVDKDGKPIPPGPGTKPSVDKDGKPIPPGPGAKPTVDKDGRPILPGPETQPSVDKDGKPIPPGPGAKPAVDKDGKPIPPGPGTKPAVDKDGKPIPSGPGTQPSVDKDGKPIPPGPGTKPSVDKDGKPIPPGTSGQTGAGGPGTKPTVDKDGKPIPPGPGAKPTVDKDGKPIQPVPGAKPTVDKDGKPIPPGPGTKPSVDKYGMPIPPGPGTKPAVDKDGKPILPGPETQPSVDKDGKPIPSGPGAKPAVDKDGKPIPPGPGTKPAVDKDGKPIPSGPGTQPSVDKDGKPIPPGPETKPSVDKDGKPIPPGTSGQTGAGGPGTKSTVDKDGKPIPPGPGAKPTEDKDGKPIQPVPGAKPTVDKDGKPIPPGPGTKQCVRDRRLPRHT |
| Aa-21179 | MLDTLKFFIFVLLLLITILLNQNVTSKKSGERDISQFWRIENENGTLKIENQTIPSGIYSALEQALIIESVLDFKNDLTTKWIARDNWTYSVPLTCNIKELNFTNVVLTLHGVDTFSKVYLNEELLGETSNMFVRYRYNVKNALRNECKNSPELRIQIRSPVSEASLLASQYDQLKIVPECPPPRYNGECHVNMIRKMQASFAWDWGLAVPSMGLWKPVRLEYYDSAKIRDITFALFDEETHWRMLVGVYLESGTTKRKLQGTLMFKILGVQLENEIVNVSKNSDESGELFVEHTLKVNKTSVELWWPNGYGRQKLYNLYVKWEDNAINNVQLHYRETLIDEKVIRIGFRTIQLSQERTSDGLMFYFIVNSVPMFMKGSNWIPSSVLPESSYDENYVKFLLYAARDANMNMLRVWGGGVYESDYFYQLADELGILIWHDMMFACSMYPANEAFLNSVKVEVIQNVRRIQYHPSIAIWATNNENEVALRQNWYGTNVNEDVYIEEYKKLYVSVIQPRIAKVDKWRAILISSPSNGDQSTKEGYISKNPQDPLYGDVHYYNYIHDGWNPVIYRGGRFISEYGFQSFPALTSWPVRDLRNEELTDLIEHRQHSPLGNVPILHMIDENLPMPSNESTDYWRDIIYLSQISQAMIVKTETEVYRSKRIEHGTMGALYWQLNDVWIAPSWSSIEYGGKYKILQYWIKDVFAQSHVIAYINAMNKLDVYLVRDTLGSDEVWTVEVNIHQWDKFMRVDNLTFHSIKVPENTVVRIGTYDIYDHLRQKNMDPKEHLLLINLLRDGTKIAENFVLLKKIKTATQIADPSPRPPLGSRAPHWWPHYSWPRLS |
| Aa-21184 | LASCLLLVVICYLLSGIVPPSSAIVGGYPEEIEAAPWMVSITLQNVGLICGGVLVSPRLVLTAAYCVFNRRATELSIRVGSKFADRDGYVYYPENIIIHNQFNATIKDYNLALLTLPVVLRESRDVYPVKIREATEYLPVGSQCIISGYGSTDAFNPQAFKGLKSAMVRVLDRENCAQRMYPWAVTPSMMCASGNGEDGCAGDGGGPLICAGKLTAIISWGKGCGRDEGIGVYADLTSSRRWMTDYGVP |
| Aa-21220 | TNLLSLLNKHNPYGSHLLSSISSISLSSLRWMFGVGFKFGFFVAFLPSGPSSCGLSLPFGILPGGFLVGRFPGFAVLAAPLGVVPAPVLPGSPGLPSLPVLGVVVPTTIISSPSMPDLLSLSSLGGVSGLPSPDSPVLPGGIGLPSLSTLGCVSGPGGIGLPSLSTLGSVPGPGGIGLPSLSTVGFVPGPDGIGLPSLSTLGCVSEPGSIGLPSLSTAGFAPGIGLPSLSTVGFAPGADGIGLPSLSSLGFVPGPGKIGLPSLSTASFAPGPGGIGLPSLSTLGFVPGPGGIGLPSLSTAGFVPGPGGIGLPSLSTLGFVPGPGGIGLPSLSTLGLVPGPPAPVSPEVPGRIGLPSLSTLGCVPGPGGIGLPSLSTLGFVSGPGGIGLPSLSTLGCVPGPGGIGTKPSVDKDGKPIPPGPGTKPSVDKDGKPIPPGPGAKPAVDKDGKPIPPGPGTKPRVDKDGKPIQPGPGAKPAVDKDGKPIPPGLGAKPAVDKDGKPISPGPGAKPVVDKDGKPIPPGSGTQPSVDKDGKPIPPGPGAKAVVDEDGKPIPPGKTGESGLGSPETPRSEDKDSKSGILGEEIIVVGTTTPSTGKDGKPGEPGSTGAGTTPSGAAKTAKPGKRPTKKPPGKMPKGKDKPHDEGPLGKKATKKPNLKPTPNIQR |
| Aa-21221 | PIPPGPETKPSVDKDGKPIPPGTSGQTGAGGPGTKPTVDKDGKPIPPGPGAKPTEDKDGKPIQPVPGAKPTVDKDGKPIPPGPGTKPSVDKDGKPIPPGPGAKPTVDKDGRPILPGPETQPSVDKDGKPIPPGPGAKPAVDEDGKPISPGPGTKPSVDKDGKPIPPGPGAKPAVDKDGKPIPPGPGAKPAVDKDGKPISPGPGTKPSVDKDGKVIPPAPGTQPSVDKDGKPIPSGPGAKPTVDKDGKSIPGAKPAVDKDGKPIPPGPGAKPAVDKDGKPIPPGPGTKPGVDKDGKPIPQGPGAKPGVDKDGKPIPPGPGAKPAVDKDGKPIPPGKTGESGLGSPDSSEDKDSKSGILGEEIIVVGTTTPSTGKDGKPGEPGSGGAGTTPSGAAKTAKPGKRPTKKPPGKCQKAKTNRMMKDRWAKRLRKTKLETNTEHPA |
| Aa-21222 | MELAYRLYLQSVLLQGQVELACRLCLHLVLFQTTDSSKPIPPGPGTKPGVDKDGKPIPPGKTGEPGLGSPETPPSEDKDSKSGILGEEIIVVGTTTPSTGKDGKPGEPGNGGAGTTPSGATKTAKPVKDGKPIPPVPGTKPSVDKDGKPIPSGPGTQPSVDKDGKPIPPGPGTKPSVDKDGKPIPPGPGAKPTVDKDGKPIPPGPGTQPSVDKDGKPIPPGPGTKPSVDKDGKPIPPGPGTEPSVDKDGKPILPGTSGETGAGGPGTKPSVDKYGKPIPPGPGTKPSVDKDGKPIPPGPGTKPSVDKDGKPIPPGPGTKPSVDKDGKPIPPGPGTKPSVDKDGKPIPPGPGTKPSVDKDGKPIPPGPGAKPAVDKDDKPISPGPGAKPVVDKDGKPIPPGSGTQPSVDKDGKPIPPGPGAKAVVDEDGKPIPPGKTGESGLGSPETPRSEDKDSKSGILGEEIIVVGTTTPSTGKDGKPGEPGSTGAGTTPSGAAKTAKPGKRPTKKPPGKMPKGKDKPHDEGPLGKKATKKPNLKPTPNIQRKLDKLIDDIEDNKCDP |
| Aa-21223 | QRPLTVSHLLSSISSISLSSLRWMFGVGFKFGFFVAFLPSGPSSCGLSLPFGIFPGGFLVGRFPGFAVLAAPLGVVPAPPLPGSPGLPSLPVLGVVVPTTIISSPSMPDLLSLSSLESGLPSPDSPVLPGGIGLPSLSTAGFAPGPGGIGLPSLSTPGFAPGPCGIGLPSLSTPGFVPGPGGIGLPSLSTAGFAPGPGGIGLPSLSTAGFAPGPGGIDLPSLSTLGFAPGPGWNWLTVFVYTRLRSWSWWNTLPSLSTLGFVPGPGEIGLPSLSTAGFAPGPGGIGLPIPPGPGTKPGVDKDGKPIPQGPGAKPGVDKDGKPIPPGPGAKPAVDKDGKPIPPGKTGESGLGSPDSSEDKDSKSGILGEEIIVVGTTTPSTGKDGKPGEPGSGGAGTTPSGAAKTAKPGKRPTKKPPGKMPKGKDKPHDEGPLGKKATKKPNLKPTPNIQRKLDIINQFIEDNKCDP |
| Aa-21871 | MAEPKADIALIGLAVMGQNLILNMDSKGFVVCAYNRTVDKVKHFLDNEAKGTKIIGANSLQDMVNKLKKPRKIMLLVKAGSAVDDFINQLLPLIEKGDVIIDGGNSEHQDSARRYEELKAKGILYVGSGVSGGEEGARYGPSLMPGGHPDAWPLIKDIFQAICAKSNGDPCCEWVGEGGAGHFVKMVHNGIEYGDMQLICEAYHLMLALGMTQKEMAQEFDEWNKGVLDSFLIEITRDILNYKDEDGYLLERIRDTAGQKGTGKWTAIAALHHGVPVTLIGEAVFARCLSALKEERSKASKQLSGPNVKATVADRKAFLTHIRNALYCAKIVSYAQGFMLLREAANEYKWNLNYGGIALMWRGGCIIRSVFLGNIRDAFVRNPQLSNLLLNDFFKDAIVSNQQSWREVVSHAVLWGVPVPAMSAALAFYDGYRSERLPANLLQAQRDYFGAHTYELLGKEGKFVHTNWTGKGGNVSASTYLA |
| Aa-21938 | AVFLAIFPAVFLPVFLAIFDCSLLSRRASPWEECTERCCQFCLSWFSAPPLTMDPSFSGHGLLAGTKSSTLLEIETKHIIAPLIVTIPGALDNKQAEQLVQNLVELLKIHLGDSMPKTNRTVIEQTGYGVFNNSDAVLKMLMKKQAERERARERKKEEEKVAAGIKKVGKLIDDAEKAAKKAEKAKKDKEKKGSNKKEPDWYVKKPRLPTYKPFELKKSHEHDRKKVAKNIAKKTAEKIARHTARN |
| Aa-22540 | PFLLFLLVLAGLSPSALSLECKSCESTIGWDDCQANVFLEDCATISPNETDPIHQCFQLEEIDSATNTVRYRQGCTGDAAFCTARPPGSIKQCSLCTDDSNSSECTSLVHRVRKTSDGKLVDVVDTIHFQRTEADVEPATSGRAATNASTTIDIASRKNDTNESFWLK |
| Aa-22559 | MVVSDSAPEHRAEAVYHQLTEGRSSGSECHRLRHRRQQRYHISGGVLLGLCSLALFLLSQSIPSSSGVLLNGTDGALDSLADLISSNDVDYVNANNDDDGDDNVSSNNYNASTDAVISSATGSDRNFGRRPNSGGGRNRPVPIYLNEFAVYIPSGPDVATVIADKYGFTNFGQIGPLKGYYLFHHSHVRKRSLDRSEVHHSALNTEPEVRWMQQQHEKVRRKRDYSTFDQDFVRFPDPRSIAPGSRVNYRDTGSHNIFPDPLFKEQWYLNGGAKDGLDMNIGPAWQKGYTGKGVVVSILDDGIQRNHPDLLQNYDPDASYDINGNDSDPMPRDNGDNKHGTRCAGEVAAVAFNNFCGVGVAYNASIGGVRMLDGTVNDAVEAKXXGLNPDHIHIYSASWGPEDDGSTVDGPGPLARRAFIFGVTSGRQGKGSIFIWASGNGGRYTDSCNCDGYTNSIFTLSISSATQGGYKPWYLEECSSTLATTYSSGTPGHDKSVATVDMDGSLRPDRICTVEHTGTSASAPLAAGIAALALEANPSLTWRDMQYLVVLTSRPEPLEKESGWILNGVKRKVSHKFGYGLMDAGSMVSLAEQWTSVPPQHICKSREINEDRPIEGSVGYTLQTHMDVNGCAGTVNEVRFLEHVQCKITLRFFPRGNLRILLTSPMGTTSTLLFERPRDVTKSNFDDWPFLSVHFWGEKAEGRWTLQILNGGRRRVSQSGILSKWQLIFYGTDSNPIRLKNEAQRGSIANYGNVNPFVFPTETDINQPALSGAGNYYQSDIYSNSMSYPFLFSGAGSSIDKTVTTLNGHNIPTAQRENVMADSNNKLVVLHDCDPECDSQGCYGKGPTQCVACKHYRLDNTCVSRCPPRSFPNQGGVCWPCHESCETCAGAGQDSCLTCAPAHLYVTDLAVCLQICPDGYYENYDNRTCVPCEANCASCQDRPDYCTSCDHHLVMHEHKCYSACPKNTYETEDYNCADCHPSCMTCNGSSESQCILCRAGRFAHEGRCLNACPDGFFGDKKRHECMPCPIGCSTCSSGSICTGCRDNWTMNKKAKCIANGSNNCDESEYYENGHCHSCHSTCETCTGPTEHECLTCASPLLLQNQRCVNECDEGYYMEVGVCAKCLHTCTQCVSRMNCTSCQKGLQLQSGECRTTCADGYYSDRGTCAKCYLSCNTCSGP |
| Aa-22560 | MNIGPAWQKGYTGKGVVVSILDDGIQRNHPDLLQNYDPDASYDINGNDSDPMPRDNGDNKHGTRCAGEVAAVAFNNFCGVGVAYNASIGGVRMLDGTVNDAVEAKALGLNPDHIHIYSASWGPEDDGSTVDGPGPLARRAFIFGVTSGRQGKGSIFIWASGNGGRYTDSCNCDGYTNSIFTLSISSATQGGYKPWYLEECSSTLATTYSSGTPGHDKSVATVDMDGSLRPDRICTVEHTGTSASAPLAAGIAALALEANPSLTWRDMQYLVVLTSRPEPLEKESGWILNGVKRKVSHKFGYGLMDAGSMVSLAEQWTSVPPQHICKSREINEDRPIEGSVGYTLQTHMDVNGCAGTVNEVRFLEHVQCKI |
| Aa-23220 | MDFYYLPGSAPCRAVQMTAAAVGVELNLKLTNLMAGEHMKPEFLKLNPQHTIPTLVDXXFSLWESRAIMGYLVEKYGKDDKLYPKDPQKRALVNQRLYFDMGVFYQRFGDYWYPQIFAKQPANPDNFKKMEEAVGFLNTFLEGHQYAAGDELTIADLSLAASAATYEVAGFDFSKYPNVQAWLERCKKNAPGYDLNQAGADEFKAKFLSGM |
| Aa-23254 | KDELGKKCDTHSFQYILTEGPTVKKNGAPKENKNKFEEYSEGLRDYQVAQIAKLDAENAEQVYKAVLKDNPNFVGAHLALIDNLDSNDLKTNLPLTFASNLDRSDKNAAALLKVKLIKVIELADLTLKEIDQNALLAYYGLKTDSRPNAAKIKTQMDKQKQQLLDAAQKKLIALSKLRVIKTLVDSNEVGD |
| Aa-24272 | MAPPNNEFTKSEVIVPREVIQQDDVRGQQQQQQQQQPTKPTMDWEAPAAQGLYDPQNEHEACGVGFIVSIEGKANHKILRDAQTLAIRMNHRGACACDNDTGDGAGVCTSIPHNLYAKDLASRGLQLPELGRYATGIFYLDKNSHEEAKKDFNTLAESLGIQVICWREVPTHQEAVGAVARKSEPMSEQVFVTADVDEETFKRQVFVLRKRATHELVRPGRRFYICSLSTKTIVYKGLFTSDQLWEYYLDLKNPDFMTYLALVHTRFSTNTFPSWERAHPLRVLAHNGEINTLRGNVNFMKAREGVMKSEQYGEELKKLYPVVEPNLSDSGSCDCVLEFLTQVGNRSLPEAVMTMVPEAWQNDRTMSQEKRDFYHWSACVMEPWDGPALISFTDGRYIGAILDRNGLRPSRFYVTRDNLLIMASEVGVYDVDPKDVTLKSRLKPGRMLLVDTEKKALIQDVELKSQIATSRPHSEWLQQQITMDEIRQASAELNGCTELSAYRLSIEQSGMMDPRLQLYGYTTETIHMLLLPMIKNKKEALGSMGNDAPLACLSAFQPLPYEYFKQLFAQVTNPPIDPFREKIIMSLQCPVGPEANLLQANPLQVHRIWLDNPILSIPDTETLKRNVHRGWKTKVIDITFLAREGPDGYLNALRRICSEAQSAAQGGYQLLMLSDRASSKERAPVSALLSLGAVHHHLIETRQRMKVGLVVETAEAREVHQMCVLLGYGADAICPYLVFELAEALRDETVIDPTLTDDEIYKAYAQAVETGILKVMAKMGISTLQSYKGAQIFEAVGLGSDVIDFCFRGTQSRIGGVSLEVLAQEGLQRHELVYGHHSVDMKILRNPGQYHWRAGGEGHINEPAAIAALQEASINENKGAYARFRDTTMNSVQQCALRGQLEFIKGRPKIDISEVEPASEIVKRFATGAMSFGSISLEAHSTLAITMNRIGGKSNTGEGGENADRYLHQNPQNNMRSAIKQVASGRFGVTAAYVANADDLQIKMAQGAKPGEGGELPGYKVTTDIAKTRHSVAGVGLISPPPHHDIYSIEDLAELIYDLKCANPKARISVKLVSEVGVGVVASGVAKGKAEHIVISGHDGGTGASSWTGIKSAGLPWELGIAETHQVLVLNDLRSRVVVQADGQLRTGFDVVVAAILGADEFGFSTAPLIVMGCTMMRKCHLNTCPVGIATQDPVLRAKFAGKPEHVVNYFFMLAEEIREIMAGLGLRKFQDLIGRTDLLQVREDLTNKAALLDLQMLLKNALDLRPGTNIIGGSLKQDFALEKRADNMLIEKCRGVINGTQQSITMDMDIKNEERAFTSTLSYEIAIKYGDEGLPNGRSININLQGSAGQSFGAFLVKGITLNLIGDSNDYVGKSLSGGTIIIRPPEESPFESHLNVIVGNVCLYGATSGKAFFRGIAAERFCVRNSGVTAVVEGVGDHGCEYMTGGMVLILGLTGRNFAAGMSGGIAYVLDVDGSFRSKVNPGMVELLGLDTDEDRETVKVLMEEFVETTGSEVAKDLLTRWPESCNRFVKVFPYEYQKVLKALKEEKALQQNIKPIMNGTPRHEPKVKDIEESIQDAALAKKKLDQMLDKTRGFIKYKRETSIYRNAEERQQDWSEVYNFPHVRKNLKVQAARCMECGVPFCQSNSHGCPLGNIIPRWNDLVFNGSWKEAIAQLLQTNNFPEFTGRVCPAPCEGACVLGISEPAVTIKNIECTIIDHAFEQGWIVPQIPVERTGKRVAIIGSGPAGLAAAQQLNKAGHVVTVYERNDRPGGLLQYGIPTMKLSKEVVKRRIDLMKAEGIEFKCNVHIGKDIMPSQLEQENDAVLFSTGATWPRDLNLPNRDLKGIYFAMEFLEASQKKLLGSRPDWISAEGKDVLVIGGGDTGCDCIATSLRQGAKSITTFEILPTPPEKRAQDNPWPQWPKIFRVDYGHEEVRVKWGNDPRQYSTTTKEFVSDGNGNIKGVNTVQVEWTQSPTGQWSMKEVAGTEKYYPADLILLAMGFLGPEKLAPTEMKLELDGRGNIKTPVGTYGTANPKVFAAGDCRRGQSLVVWAITEGRQAARQIDSYLIGKPSSLPGPGGVIDLRSP |
| Aa-24416 | MAQTVDAQFVRKTNDFALDLYKQIISNEKKNVVISPFSISTCLSLAAMGAGGLTAEEMFRGLKYDPAQKSAIAESYGNVMTNLDGNKSLKIANKMYIMEKYSVKANFHEIAQKGFRSEAESVNFSDNTAAAKKINTWVEQKTNDKIKDLISPDSLDDMTRLVLVNAIHFKGTWTHQFNPASTRPMPFWISETESVDVPMMNTKKHFKHGVFDDLGLAALEMTYNDSDVSMLILLPHERTGLTKLEENLQNIDIPDMLTKMYSQEVEVFLPKFKIEFDLDLKETLEKLGMGTMFSDSADFSELLEQHDPLKVSKVVHKAFIEVNEEGAEAAAATGMIMMTRCMIIHPYFTVDHPFLYILKQNDQMFFIGRMNQI |
| Aa-24496 | MLRTLLLVAATSALCFVLVYGSYTPRSKPGQSGGRIVGGIAVNITDYPYQVSLQRNNHFCGGSVLNDRWILTAAHCTKGITNASVLRIRAGSTEVRSGGVLAQVRTIYFHPKQNSWSNYDFSLLELKEALKLSKAVQPISLPAHGDSFEDGTLCEVSGWGNTRNANESSLSLRAATVPLYNQEKCSTVYKEYGGVSESMICAGYEEGGKDSCQGDSGGPLVCNGVLVGVVSWGKGCAEPGFPGVYGRVTSAAEWISETMNEVVSER |
| Aa-25684 | LGKLDAYVEQSTRKIAAYLXXVLEDQRDKLYENLQANNNDLTTYITRFQWDLAKYPTKQSLRNIADIISKQVGQIDADLKTKSAAYNNLKGNLQNLEKKQTGSLLTRNLADLVKREHFILDSEYLTTLLVIVPKQMVNDWNANYEKITDMIVPRSSQMITQDNDYALCTVTLFKKVVDEFKLHARERKFVVREFTYNEEELAAGKNEITKLVTDKKKQFGPLVRWLKVNFSECFCAWIHVKALRVFVESVLRYGLPVNFQAILIHPNKKNTKRLRDVLMQLYGHLDGSAASSGGNADNVDIPGLGFGQSEYYPYVYYKLNIDMVENKV |
| Aa-25757 | MSQALPIRFQEHLQLTNININPSSISFTNLTMESDKFICVREKIGETAQVVIIDMNDAQNPIRRPISADSAIMNPASKVIALKAQKTLQIFNIEMKSKMKAHAMTEEVVFWKWITLNTLSLVTETSVYHWSMEGDSTPVKMFERHSSLNGCQIINYRTDPKQAWLLLVGISAQQNRVIGAMQLYSVERKVSQAIEGHAASFATFKMEENKEPSTLFCFAVRSANAAKLHIIEVGAPPTGNTAFTKKAVDVFFPPEAQSDFPVAMQVSPKYDVIYLITKYGYIHMYDIETGTCIYMNRISADTIFVTAPHESSGGIIGVNRKGQVLSVTVDEEQIIPYINTVLQNPDLALRMAVRNNLSGAEDLFVRKFNQLFQNAQYAEAAKVAAIAPRGILRTPQTIQKFQQVPAQPGTNSPPLLQYFGILLDQGKLNKYESLELCRPVLAQGRKQLCEKWLKEEKLECSEELGDLVKPSDPTLALSIYLRSNVPNKVIQCFAETGQFQKIVLYAKKVNYSPDYVFLLRSVMRTNPEQGAGFASMLVADEEPLADINQIVDIFMEQNMVQQCTAFLLDALKNNRPSEGSLQTRLLEMNLMSAPQVADAILGNAMFTHYDRAHIAQLCEKAGLLQRALEHYTDLYDIKRAVVHTHLLNGDWLVGFFGTLSVEDSLECLKAMLTANIRQNLQICVQIATKYHEQLTTKALIDLFESFKSYEGLFYFLGSIVNFSQDPEVHFKYIQAACKTNQIKEVERICRESNCYNAERVKNFLKEAKLTDQLPLIIVCDRFDFVHDLVLYLYRNSLQKYIEIYVQKVNPSRLPVVVGGLLDVDCSEDIIKNLILVVKGQFSTDELVEEVEKRNRLKLLLPWLESRVHEGCIEPATHNALAKIYIDSNNNPERFLKENQFYDSRVVGRYCEKRDPHLACVAYERGHCDRELIAVCNENSLFKSEARYLVRRRDAELWADVLSEANPYKRQLIDQVVQTALSETQDPDDISVTVKAFMTADLPNELIELLEKIVLDSSVFSDHRNLQNLLILTAIKADRSRVMDYINRLDNYDAPDIANIAINNELYEEAFAIFKKFDVNTSAIQVLIEQVHNLERANEFAERCNEPAVWSQLARAQLQQGLVKEAIDSYIKADDPSAYMDVVETASKNESWEDLVRYLQMARKKARESYIESELIYAYARTGRLADLEEFVSGPNHADIQKIGDRCFNDKMYEAAKLLYNNVSNFARLAITLVHLKEFQGAVDGARKANSTRTWKEVCFACVDAEEFRLAQMCGLHIVVHADELEDLITYYQDRGHFEELIGLLEAALGLERAHMGMFTELAILYSKYKPAKMREHLELFWSRVNIPKVLRAAEQAHLWSELVFLYDKYEEYDNAVLAMMAHPTEXXXEGHFKDIITKVGETIELYYKAIQFLVSDSNRFC |
| Aa-25949 | MEKSNLLFKSASLALVLVAVFQLVPASPLHEAEDPITVPSIQFRSKTDKDLCSAYQLNGKMFIALTRCLGNEASRINQLTELDVVGKGQQTIQAFALQPAGTDSLGDLAVIMYTGAIDGEDQPRDSSAGLFEKFGLRSLVRFGRRSRQDEPAAGAADNATDSAPFTGKNAQLIGAALTFVGSVLLMQ |
| Aa-26716 | MSEINPEFCGYRSPLSTRYASKEMQYLFSEQNKFSTWRKLWIILAKAQKELGLEISDAQIAEMEAHIEDIDFPAAAEEEKLTRHDVMAHVHVFAKQCPLAAPIIHLGATSCYVGDNTDLLVLKGGLDSLLPKLVGVIKRLSEFAVEYRDLPTLGFTHLQPAQLTTVGKRCTLWVQDLLMDERALRNCRDNLRFRGVKGTTGTQASFLQLFAGDGDKVRQLDQKVTNLAGFEKYYAVTGQTYTRKVDLEIVSALSSLGATVHKMCSDLRLLASRKEVEEPFEKTQIGSSAMAYKRNPMRSERCCALARHLITLHASAANTLAVQWLERTLDDSANRRLTLSEAFLSADACLLTLLNISQGLVVYPKVIERNIAQELPFMSTENVIMAMVQAGGDRQICHEKIRVLSHEAGAQVKQHGKDNDLVERIRGDAYFAPILGDLDKILDPKTFTGRAADQVLEFVREEVDPVVARYGSNVETKSIGLAI |
| Aa-26944 | MPRLQSVYVLLAVVLLQTSSRAHTQNDELKLKRDNHRPKEISVSGLQHLSELSRNSELNEILKHLLVERVVGTPGHENVKNYIVNYLRKLDWQVELDEFQDETPTFGKLGFANIIATLNPNAERFLVLACHYDSKYFKDQVFIGATDSAVPCAMMLDLAQSLKAQLNKKKLDNSLSLQLVFFDGEEAFHQWGPKDSIYGARHLAARWEAEDKLKRIDMLVLLDLIGTPDPNFYSYFQNTESWYSQLLSAEERLERAGHLERYSYSSVAPNQQTVRYFQPHSYYAYIEDDHIPFLQRQVRILHVIPSPFPDVWHKLSDDANAVDIGTVQNLIKVFRVFLVEYLHLAV |
| Aa-27539 | MQHIKTTFVLCALFITSALSQNGDNAEEVGNHFEGDMILNPEQLMATDDELRNVMIKSKYRWPHNTVRYRIDIEKFGPSQLEYIRKAMDTIESVSCIKFVEAGQMAKKYVKIGFEKPGCFATIGYQGKPQTLNLTPTRLESGCFRLGTIMHELLHTLGFYHQQSATNRDQYVKILWKNIEPEQKHNFKKYSYDKVTDFNIKYDYGSIMHYVRKAFSMNGEPTIIPKVPNVFIGQRLKLSEGDIMKLNRLYGCRTK |
| Aa-27575 | MTVDKGHSNTAFVGDEQVTSKKSAPVKTISGNNYVLEIQEKKPGFEADYNPYEHRHVEHPTTSNETLIHLLKGSLGTGILAMPNAFHHAGWLVGSVGTLLIGILCTYCIHLLIKAEYELCRRKRVPSLNYPAVTQTALLEGPNALKPLSNIIIHIINVFLLVYQLGTCCVYVVFVASNIKAIADYYTETPTDVRLFMLIILLPLILINWVRNLKFLAPFSTLANFITLVSFGIILYYIFREPVTFEGKEAVGKITEFPLFFGTVLFALEAIGVILPLENEMKKPKQFGGNFGVLNKAMVLIVTLYIGMGFFGYLNYGADAKGSITLNLPEHEILAQCVKGMLAFAIYITHGLACYVAIDITWNDYAKKRFGESPRSVFYEYIVRTVLVLITFLLAVAIPNLELFISLFGALCLSALGIAFPALIQTCTYWHERHGWDKTWMIIKNVIIGIIAIVGLVVGTTTSLKEIVQTFSRKNRLASWIDPLALVSSDDEEMSAVIRNDDDDDGGSRWT |
| Aa-28317 | MDFYRILAVFHSNRVPRNSAFRIERIREFEEPLYYIKLISSREYVFGGDMSTARDNLRLPVFSYHHQVNTTRQDDGIWGFEKAYVNRVWRGAWYIKNIYYDNYLIASTVHADPPPMATNARFKTLKLDRRRQVPLTNAHMFYIEDC |
| Aa-28387 | QYYILLYSTPTSITVFENTVNAISNYILHPAHFPHPPHPPPPPPHPHPLRFFLFGSSPFAFYGRCPNEPMFLPLESARGTELLGKPKAFADFQHCLETCAVFRPVCGKTSTLWXXXXXXXXXXXXXXXXXXXXXXXXXXXXXXIESLLLVAALSNVHTTYVVYPPKYELSDSQTTELMSCLGPTENEADPQSKYEPICGTDGFSYYNKHQMKCLARTIPSVAFAFYGRCPNEPMFLPLESARGTELLGKPKAFADFQHCLETCAVFRPVCGNDLKTYTSPCALECAKAHRPTLELKGNGFCLEDQSVDGKCPEILQPFCGTDGITYLNYCHLKFAQLHAKELLPAHMGDCVRRLITVKGVSS |
| Aa-29081 | MTRACRLLWIVSITLCLAAVQLAQAQNPRCCEDYCYSRDHDRSQAKRFATKTSYELIHGSSSSREHIVPNCIPSKLWLLSRHGTRLPGKKDIQVLPQALNNLRESILDNYDNRRTAPDIGRMCADDLDLLRSWRWDRNISVEYESFLTDQGWSDLKLLARREKDRFNEVFNWPYDKQRYLFRHTKSQRTEASFKAFVEGLFGDGAYNFINAEPEPTDDTLLKPYDFCPAYDANKDKNKQPDSELNKFLRSRIYLQTLSDISTRLGFRYNLSNDQIEAMWDICRYEQAWHLQQYSPWCSVFTKNQVNILEYKEDLRYYYQNSYGYEKSADLACYAVNDMVKNLGRSDGQQVIAYFTHEAEIQIFLTALGAKKDRDSLRAENYYAMQNRNFRSSELTPFAANLAAVRYQCADPVEPVKVIFFLNEKALMFDWCRVGLCDWSEVVRRYERYTKADCAKMYCGGSGASSWTLCWTTMIGIVLTIAVGLLQR |
| Aa-29155 | MKFVLIESLLLVATLSYVHTTYVVYPPKYELSDSQTTELMSCLGPTENEADPQSKYEPICGTDGFSYYNKHQMKCLARTIPSVAFAFYGRCPNEPMFLPLESARGTELLGKPKAFADFQHCLETCAVFRPVCGNDLKTYTSPCALECAKAHRPTLELKGNGFCLEDQSVDGKCPEILQPFCGTDGITYLNYCHLKFAQLHAKELLPAHMGDCVRRLITVKGELPNKRKKSGCGCGGGGGGCGGCGK |
| Aa-30099 | IVFDDTDAGGSSTAQITARVQTDITKTLHDEGLAAPARGGSNHGHVVGLLDEVVQSVENTATGGRDTSVDTALVDWLAGDASVGIDVQMADRLGVGISNPGHFTLASSHIRGRHINAHACVTGKPINQGGIHGRVSATGRGVFHGLDNFIKEANYMAMIGTTPGWGGKTFIVQGFGNVGLHSCRYLCRAGATCIGIIEHDGSIFNPQGIDPKALEDYRNEHGTIVGFPGAMPYEGENLMYEPCDIFIPAAIEQVITSENANKINAKIIAEAANGPTTPAADKILIDRNILVIPDLYINAGGVTVSFFEWLKNLNHVSYGRLTFKYERESNYHLLASIQESIERYIHEDQSVQASLERRFGNVGGKIPVTPSEAFQKRISGASEKDIVHSGLDYTMERSARAIMKTAMKYNLGLDLRSAAYVNSIEKIFQTYRDAGLAF |
| Aa-30184 | SYDTTRHVSVPKNVDHLCGPPSNLCLNTHHSKTPRSVTVRRKDHPKRRTLIVQKPIGNIECGEVVAYMRSLSRFYGRRCQQQGSECRDRDTYPSRWFDKVGTKITGMCQRLEMYERGEKPVGAPAWDTSDKKGDGKKCKPKNSIL |
| Aa-30185 | AIAYRQRPLHVMFPFRKTLIILVVLQVTCALIPIIPRPPVSELCAAKPPKVNTNRQKPIGNIECGEVVAYMRALSRFYGRRCQQQGSECRDRDTYPSRWFDKVGTKITGMCQRLEMYERGEKPVGAPAWDTSDKKGDEKKCKSKNSIL |
| Aa-30266 | LWQSLLQQFLFKGVELRQSQVLLQTVLAQHQRRGEVLGFGHIGADVGALNHVLFALHGAGQSQSEPSSSVGHRQGSRTSSGLGLDDFGTGLLDALGIGQPLSLLLKQSPLVTELSLYDIVHTPGVAADLSHIETHSKVTGYNGAENLEKALANADIVIIPAGVPRKPGMTRDDLFNTNASIVRDLAAGCAKACPKALIGIISNPVNSTVPIACETLAKAGVLDVKRVFGVSTLDIVRANTFIGEAAGVDPQKVNVPVIGGHSGVTIIPVLSQATPSVSFPQDKIAALTERIQEAGTEVVKAKAGAGSATLSMAYAGARFALALARAMKGEQNVVECAYVRSDVTEAKYFSTPLVLGKNGLEKNLGLPKLNAFEQELLKKALPELKKNIQKGEDFVNKK |
| Aa-30847 | EAFFVPVETILQKQDLRQVLLITSVGLCSSTINYCDRSLCGRGQHIGCNATKGFAHACGKNAEYIPMDAKLKEIILNKHNTLRAKMAQXXXXXPPAQRMPTLVWDDELAPVASFNARRCKFRHDNCRNTKEFKYSGQNLAMARFRGKAVNPDERAAFFLQKWFDEYRHCPKSIIAKFPLSYKGPQIGHFTLMMNDRVWKVGCSMVRYKSGKWTNLFFVCNYSMNNILGRPVYTGGKTASKCQTGQNAKFKGLCSTKEKV |
| Aa-31529 | DCEGDPENCISENLFRTMADLVVSEGYAALGYEYINVDDCWLEKSRGPRGELVADRRRFPSGMKALADYVHAKGLKFGIYEDYGNYTCAGYPGILGFSQNDAAQFASWDVDYVKLDGCYSLPIDMDHGYPEFGRNLNATGRPMVYSCSWPVYQIYAGMNPNYSSIIQHCNLWRNYDDIQDSWASLESIIDYYGNNQDAIIPNAGPGHWNDPDMLIIGNFGLSYEQSKTQMALWAIMAAPLMMSVDLRTIRPEFKAILQNKKIIAVDQDPLGIQGRRIYKHKGIEIWSRPITPIYQTFYSYAIAFVNRRTDGTPSDVAVTLRELGLISPTGYRVEDLYEEVDYGILSPQTKIKVKVNPSGVVILRADVQPERFSKRPYNPIFYRYPN |
| Aa-32239 | MSTLKKISDEDRESKFGYVFAVSGPVVTAERMSGSAMYELVRVGYYELVGEIIRLEGDMATIQVYEETSGVTVGDPVLRTGKPLSVELGPGIMGSIFDGIQRPLKDINELTSSIYIPKGVNIPCLSRTQSWGFNPLRTYVGKGSVYQQLYPEPGKGSTSLRVLRGFGRPKNRKTYLRFGNERPKPKRLGLGPTPTRPARTG |
| Aa-32457 | MWLSGLTAIILGVKLTQTNYSEEPCQEVERTEEIYDAQSVIGNVSVFCRFIQGYKDGWTTIHQRFDGTVNFTRSWDDYREGFGDLQGEYWLGLDNVHHITRSGPFELMVIMEDYNDERMWATYDKFAVLSEKEQYQLDLGQFSEGNVEDSLKLHNGMKFSTADRDNDLWSGNCAELYESGWWFKDCHAVNLNGVYQNETEYDKMMWGKKCLKEAQMLIRITKSSQ |
| Aa-32547 | MLIKSIIVLVCLAVLAQADFIRVQLHKTESARQHFRNVDTEIKQLRLKYNAVSGPVPEPLSNYLDAQYYGAITIGTPPQSFKVVFDTGSSNLWVPSKECSFTNIACLMHNKYNAKKSSTFEKNGTDFHIQYGSGSLSGYLSTDTVGLGGVSIVKQTFAEAINEPGLVFVAAKFDGILGLGYSSISVDGVVPVFYNMFNQGLIDAPVFSFYLNRDPSAAEGGEIIFGGSDSNKYTGDFTYLPVDRKAYWQFKMDSVKVGDNEFCNNGCEAIADTGTSLIAGPVSEVTAINKAIGGTPIMNGEYMVDCSLIPKLPKISFVLGGKSFELEGADYVLRVAQMGKTICLSGFMGIDIPPPNGPLWILGDVFIGKFYTEFDMGNDRVGFATAI |
| Aa-32548 | SAPSSSKDFPPKTNEIFGNLGINEQSTMYSPFIMGVPPIALLIAVTSDTGPAIRLVPVSAIASHPLLQNSLSPTLTESIVKQTFAEAINEPGLVFVAAKFDGILGLGYSSISVDGVVPVFYNMFNQGIIDAPVFSFYLNRDPSAAEGGEIIFGGSDSNKYTGDFTYLPVDRKAYWQFKMDSVKXXDNEFCNNGCEAIADTGTSLIAGPVSEVTAINKAIGGTPIMNGEYMVDCSLIPKLPKISFVLGGKSFELEGADYVLRVAQMGKTICLSGFMGIDIPPPNGPLWILGDVFIGKFYTEFDMGNDRVGFATAI |
| Aa-33502 | MDEEYDAIVLGTGLKECILSGMLSVSGKKVLHIDRNKYYGGESASITPLEDLFSRFGVQLPEGKYGRGRDWNVDLIPKFLMANGLLVKLLIHTGVTRYLEFKSVEGSYVYKGGKIAKVPVDQKEALASDLMGMFEKRRFRNFLIYVQDFIQDDPKTWKDFDPMSRNMQDLYDHFGLDKNTQDFVGHALALYRDDDYLVEPAVKTINRIKLYSDSLARYGKSPYLYPMYGLGELPQGFARLSAIYGGTYMLDKPIDEIVYDASGKVVGVRSGEEXXKCKQVYCDPTYVPDKVRIKGKVIRCICLLDHPIANTKDALSTQIIIPQKQVGRKSDIYVSLISSTHQVSAKGWFIGMVSTTVETDXXXXXXXXXLDLLGTIAQKFVCVSDYYEPTDDGLQSQVFISESYDATTHFETTCLDVLNIFKRGTGEDFDFSKIKQELGDEEQ |
| Aa-34428 | MFLRFVSILALALVAATVVEARVKVTVFYEHLCPDSIRWISNQLAPNYNALRNHIDLEFIPFGKARSINGGQSFECQHGPLECEGNRIQSCVLNQLPDQDRQVSYVTCQMSFDADPRGWECTFRSGVDLVATQNCVEGVQGVQLQLEAERRTQQIPLTFVPSFAFNDQFDQELNQLAFQDFPAALCRVDSSIAGCQ |
| Aa-34453 | MIISTRLAFLVLVVAIVSLVDSGLASPRRQQRWRNGNGFYRRDQRGNYQEYSESFGAAGKCGYESCPEIKQGMLNVHLVPHTHDDVGWLKTVDQYYYGSRTLIQKAGVQYILDSVIESLLKDPSRRFIYVESAFFFKWWKEQTPELQEKVRELVNQGRLEFIGGAWSMNDEATTHYQSIIDQFTWGLRLLNDTFGECGRPKIGWQIDPFGHSREQASIFAQMGFDGYFFGRLDYEDKRQRLSMKDPEMIWKSSANLEDSDMFTGILYNVYQPPPGFCFDILCSDEPFIDGPYSAENNVDSKVQKFLYYVELQAKHYRTNNIVLTMGGDFTYMDANVYFKNLDKLIRYTNARQSNGSNVNVFYSTPSCYLKALHDADITWPTKSDDFFPYASDPHAFWTGYFTSRPTIKRFERVGNHFLQVCKQLTALAPSKEGHFTPHLNLLREAMGVMQHHDAVTGTEKQHVANDYSRMLHQAIEACGANAQVVLNQIADPAQKKGLPQKFPHGFQRAFTFEFETCHLLNISKCEMTESKDNFMVTLYNPLAHSGYQYVRLPVSGKKYIVKDYRGVETPSQMVPVAESVEDLNYRFSNASYELVFLANELPPLGYKSYYVSRIIETVDDFIQNPSSPTVQIQSDQPHKQWHSEEVSIGNKYLNVSFDTNGFLSTITLNGVAHRLRQTFVYYEAAMGNNVAFRNRSSGAYIFRPNGTDSPIADSVQLKVFRGNVVQEVHQVFNEWVSQVVRVYADENHVEFEWMVGPIPIEDRVGKEIVSRFYTAAQSNGVFWTDSNGREMIKRKRNHRDTWDVNLEEPVAGNYYPVTAKIALEDENIRLAVLNDRAQGGSSLEDGALELMVHRRLLHDDAFGVEEALDERAFGRGLIARGKHYVVFGSKKTSSPTLQAKERFLQNHVLLPNWVFLSDVSKFKYEDWQKRFNNIYSALSLSLPLNVNLMTFEPWKDNSLLVRFEHLLEKDEDPMYSKPVRFNLQDIFRSFSIEEIRETTLAGNQWKEDNSRFQFKADPNYLKQITRAEIVNPFNGTERVVKKDDQSALENLKNVSNEGFEIVLGPMQIRTFVMQLEFRP |
| Aa-34456 | NFMVTLYNPLAHSGYQYVRLPVSGKKYIVKDYRGVETPSQMVPVAESVEDLNYRFSNASYELVFLANELPPLGYKSYYVSRIIETVDDFIQNPSSPTVQIQSDQPYKQWHSEEVSIGNKYLNVSFDTNGFLSTITLNGVAHRLRQTFVYYEAAMGNNVAFRNRSSGAYIFRPNGTDSPIADSVQLKVFRGNVVQEVHQVFNEWVSQVVRVYADENHVEFEWMVGPIPIEDRVGKEIVSRFYTAAQSNGVFWTDSNGREMIKRKRNHRDTWDVNLEEPIAGNYYPVTAKIALEDENIRLAVLNDRAQGGSSLEDGALELMVHRRLLHDDAFGVEEALDERAFGRGLIARGKHYVVFGSKKTSSPTLQAKERFLQNHVLLPNWVFLSDVSKFKYEDWQKRFNNIYSALSLSLPLNVNLMTFEPWKDNSLLVRFEHLLEKDEDPMYSKPVRFNLQDIFRSFSIEEIRETTLAGNQWKEDNTRFQFKADPNYLKQITRAEIVNPFNGTERVVKKDDQSAVENLKNVSNEGI |
| Aa-35407 | MPQCNHKKMSGNAKLLHENLWESDPELMDLIRKEKKRQVHGLEMIASENFTSLSVLQCLGSCLHNKYSEGLPGQRYYGGNEFIDEIELLAQKRALEAYRLNPEEWGCNVQPYSGSPANFAVYTGLIEPHGRIMGLDLPDGGHLTHGFMTATKKISATSIFFESMPYKVDPVTGLIDYDKLEESAKNFKPKIIIAGISCYSRCLDYKRFRQIADANGAYLFADMAHISGLVAAGVIPSPFEFADVVSTTTHKSLRGPRAGVIFFRKGVRSVKPNGDKVMYDLEAKINQAVFPGIQGGPHNHAIAGIATCMLQARTPEFKDYQVQIIRNAQALCKGLLERGYSISTGGTDVHLVLVDLRPAGITGARAEYVLEEISIACNKNTVPGDKSALNPSGIRLGTPALTTRGLVESDMASVVDFIDRGLQLSKEITAVSGPKLVDFKRVLHEDKTLNAKVQALKEEVQAYSAKFPMPGYEEF |
| Aa-35812 | MIGSILAFFSLFSTAMAANKERTFIMVKPDGVQRGLVGKIIKRFEQKGFKLVAMKFMWAEKELLEKHYADLSARPFFPGLVSYMGSGPVVPMVWEGLGVVKTGRQILGATNPADSAPGTIRGDLCVQVGRNIIHGSDAVESANKEIALWFTEKELVAWTPASEGWVYE |
| Aa-36601 | MSWQDYVDNQLLASQCVSKAAIAGHDGGIWAKSEGFEVSKEELAKIVQGFDKTELLTSGGVTLAGQRYIYLSGTDRVIRAKLGKMGVHCMKTQQAVIVSIYEEPVQPQQAASIVEKLGDYLITCGY |
| Aa-3705 | MSERENNIYKAKLAEQAERYDEMVEAMKKVASMDVELTVEERNLLSVAYKNVIGARRASWRIISSIEQKEENKGVEEKLEMIKNYRSQVEKELRDICSDILEVLDKHLIPCATTGESKVFYYKMKGDYHRYLAEFATGNDRKDAAENSLVAYKAASDIAMTDLPPTHPIRLGLALNFSVFYYEILNSPDRACRLAKAAFDDAIAELDTLSEESYKDSTLIMQLLRDNLTLWTSDMQGDGDGGEQREQVVQDVEDQDVS |
| Aa-3745 | VPGTQFSSGGVSRFVLVLKQNFIVHAAIPVSVQKQSLHPSALLKDVPGTQSFFGGCIAVSRLVLVLKQNFIVHAAMPVSVQKQSLHPSALLNDVPGTQSCFGGCGAMALEAGQRCEPGSTFKMDCNTCRCSADGMVVSCTRKFCLPELQGDDPKVQEPSGNAQSETAAVEKEEEEVHTNGQVCTPNEVKMEDCNRCKCAANGIGWFCTRKACPPRERRAVQPKMECEPGTTFRSDDDCNSCFCTETGIAACTQKACINFSSLKPKRAIAAQPPKQDCVPGTSFKSADGCNDCFCTDTGIAACTMKFCFNTKTKRDTAMQPPKKDCVPGTSFKSADGCNDCFCTETGIAACTMKFCFNTRTKRDTPPEENCVPGTTYKHADGCNDCTCIQPGKAICTIKLCPETSLRAKRFAAKSTEKQCEPGTSFKHSDGCNDCFCSDNGMAACTLKFCFFEEPTTKSKRQVNNDELPVSELAPGTPGFTWQSRQELQVIQCNTLPVRYNWTDGGVYIQVLYSGRVLIVRWTGVRAALHNSANLVL |
| Aa-3779 | IGKKSLAARFGQSADIQFTSVDKLFFGKQVAIRECMEDKFFEEIEKLDFEKDADGQRLYINNWVENVTQGEIKDLLIPGSITKQTKLAIANAAYFKGTWQTEFKPDQTNKEIFYVSSERQAFVDMMHVLGTFNHAANEKLGCHVLEMPYKGQDESTRISMFVFLPPAAPNSLDKXXXXLTSDTGILSEIVNEGRSPAPTRLGYGYRNVNPVNPVNVTTTTYGKETGTRYGRRKEPGPRFR |
| Aa-38490 | MVSTSFALTFLVILLNWCECATALTDDARIVGGYADRIENVPYTVSISKRGFGHFCGGSLISLQWVLTAAHCLVGEGPGDLYVRAGSTYKNTGGILRKVKMVIPHNRYSKDIKLDLDIGLLQLLRPFPANNDFIGTVRLIGPAEIVPPGRDCVISGWGTTKQNDGEHQVLKSAMVKTVSQAACQRVLFRKIITKNMVCAGAQRHDACQGDSGGPMICTGSLTGVVSWGEGCATAGKPGVYTSVRELRPWIYAYTGM |
| Aa-38499 | IFRYDLSKQYPLTRCLRNRFNHSTFQYLMLAVILLGGSLCGDSLISLQWVLTAAHCLVGEGPGDLYVRAGSTYKNTGGILRKVKMVIPHNRYSKDIKLDLDIGLLQLLRPFPANNDFIGTVRLIGPAEIVPPGRDCVISGWGTTKQNDGEHQVLKSAMVKTVSQAACQRVLFRKIITKNMVCAGAQRHDACQGDSGGPMICTGSLTGVVSWGEGCATAGKPGVYTSVRELRPWIFLILECKNAA |
| Aa-39719 | QQGIQQQQPQHLQSRMPNIKVFSGSSHPDLASRIVDRLGIDLGKVVTKKFSNLETCVEIGESVRGEDVYIVQSGSGEINDNLMELLIMINACKIASASRVTAVIPCFPYARQDKKDKAGDEKLAMLMKTHEWKFRSRAPISAKLVANMLSVAGADHIITMDLHASQIQGFFDIPVDNLYAEPAVLKWIRENIAEWRNSIIVSPDAGGAKRVTSIADRLNVEFALIHKERKKANEVASMVLVGDVKDRVAILVDDMADTCGTICHAAEKLREAGATKVYAILTHGIFSGPAVSRINNACFEAVVVTNTIPQDGHMKDCPKIQCIDVSMMFAEAVRRTHNGESVSYLFSNVPY |
| Aa-39723 | MAAGDPKWQKDASDQNFDYMFKLLIIGNSSVGKTSFLFRYADDSFTSAFVSTVGIDFKVKTVFRHDKRVKLQIWDTAGQERYRTITTAYYRGAMGFILMYDITNEESFNSVQDWVTQIKTYSWDNAQVILVGNKCDMEDERVISFERGKQLADQLGVEFFETSAKENVNVKNVFERLVDIICDKMSESLDSDPTLVAGGPKGQRLTDQPQGPPNANCNC |
| Aa-39765 | SPVCLGTPTTHTHCSLLYVAVPIFSLFLLEVGAKSRTYEFQNGKVTFEEAWSNCKDKGMQLATAQSLDDYNELGRLLNQPQYKDQDFWATDQDIDRNIAWTFTFNEAEMTLISQEGDWAKTRCMLVRSFFHAGTAGTDWNDDLCGQTHGYICDRL |
| Aa-4148 | MRLFCCKLLLILSVAVSTSAKSNIFSNETDHHRSKRIVNGWKIQISKVPYQVAIVFRSYIVCGGSIIAPTWILTAAHCFYDHQEVVPELKVRAGSDRRHIGGELRRIRWQTIHPQYSPKTLLYDIALVNVDSAFALNQDVVCIRLAPQGYFPMAGKMALVSGWGLENPDADKKDPSNFPIRLNYALLPIMEFPLCQQMYQKHTLSEHTQICAGYPQGGRDACVGDSGGPVVINQYQVGIVSWGISCAQPNKPGMYTNVGSFRDWIYEIMTSKTRKGALECIRYL |
| Aa-41789 | MFARYSSNFVRSSQLRRFQSTLVLAEHNNETLNPITSNAVTAAKKLGGDVTVLVAGTKVGPIAEAAAKLDGVSKVLVAEGDAFNGLVAEAVTPLVLATQNQFKFTHIVAGASAFGKAVLPRVAAKLDVSPVSEIIDVKSADTFVRTIYAGNAIQTVKSKDPVKVVTVRGTNFEAAGTGGSAAIEKAPEGNYKSDLTEFVSQELTKSDRPSLTAAKIVISGGRGMKSGDNFKMLYDLADKWGAAVGASRAAVDAGFVPNDLQIGQTGKIVAPELYVAVGISGAIQHLAGMKDSKTIVAINKDPEAPIFQVADYGLVADLFKAIPEINEKC |
| Aa-42496 | HTLSLSPSGGHKQGKSIDHFNRPNLFGPDKGTFDRAEEDEDDPDSTDASSNKSAISLPNSVYSGKPGGMNVLKMGSKVLEKLSFRLPRHIKPRHYDLQMFPDLEQQTFSGQVGIDITISEPTDYIVLHSKQLAITETLVKRKHPDRSEEPVKVKQAYEFEPHQYWVIETEGIGSGEYRLSMNFSGSLANRIVGFYSSSYKDKGSNSTRKIATSKFEPTFARQAFPCFDEPHLKATYAIQVVHPSTNNYHALSNMDANQTLTNTPSTGLSTTVFNPSVPMSTYLVVFIVSDFEYDSIRITPSLGERFELRVYTTPFQIHNARFARDTAEKVINHYIDYFNIEYPLPKLDMAAIPDFVSGAMETWGLVTYRETSILYNEETSSTANKQRVAEVIAHELAHMWFGNLVTMKWWNELWLNEGFASYIEYKGVDHAYPDWGIMEQFALDNLHGVLTLDATLGSHPIVVKVESPNQITEIFDTITYSKGASVIRMLEDFVSEPIFKEGVTKYLERLRYGNGESKDLMDELDKLFNDPSEPDLTVTAVMDTFTKQKGFPVITVTRSGTQFRLRQSRFLADPNATETEDSEFGYKWYVPLTYTTSGNPTEVKRTWMLWADDQISIDVPSGSGSWIKFNHNQVGYYRVNYPNDVWKQFGDLLSADINTLSIGDRTGLLNDAFALADASHLSYDLALELTRFLGQETEYVPWATVSSKMKTIRNLIYDYPSYDDINLYVRELIQRAYDSVGWTVVGEDHMKNRLRTTVLDLACSFGHEDCLQKAYEMFRGWLDSDVAVHPDLRTVVYYYGLQRSANYSDWELVKERFRTENDANEKAKLMSALAGYPDAKVLRRLLDDAWDPQLVREQDHLTCIQNVAANKHGEQVAWDHVRQNWDRLVDRYSLGERNLGRMIPSITGRFSTPVRLMELEDFFRRNPDAGAGAAARVQALENIGNNIKWLERNQKIVAEWLSAAMK |
| Aa-4306 | VRPVGSGPVTGNGPRPVDKPCRCFRTFYPVCGSDNRTYLSKCHLECAMNFYPGLKFVSVDYCPGADKRYVVGDIDPAAFPWSG |
| Aa-43739 | MNEVVRYNGTPSKANPTGKPGIEWRILIVDKLAMRMVSACTKMHEISAEGITLVEDINKKREPLPAIEAVYLITPSEDSIRLLMRDFENPAKPTYKAAHVFFTEVCPEELFNDICKSVVSRKIKTLKEINIAFLPYESQVYSLDSPVTFQCAYSPALASARFGNMERIAEQIATLCATLGEYPSVRYRAEWEGNMELAQMVQQKLDAYKADEPTMGEGPEKARSQLLIIDRGFDCVSPLLHELTLQAMAYDLLPIVNDVYKFIPSPNAAEKEVLLDENDDLWVDLRHQHIAVVSQSVTQYLKTFTESKRLTQTEKQSMKDLSQMIKKMPQYQKQLSKYSTHLHLAEDCMKSYQGYVDKLCRVEQDLAMGTDAEGEKIKDHMRNIVPILLDQSVSNYDKVRIIALYVMIKNGISEENLTKLVTHAQIEPKEREMITNLSYLGINVIADGNRKKGYSVPRKERINEHTYQMSRWTPVIKDIMEDSIDNKLDERHYPFLGGRKTAGFHAPTSARYGHWHKDKSQTAVKNVPRLIVFVIGGVSYSEIRCAYEVTAAVKNWEVYIGSSHILTPETFLSDLGSLNKE |
| Aa-4511 | LVCIYGQIYFSFFAAQDIESCPQPPKHYSELGCKPIQDEGHKCPNRYECPTLTDRDGEKCYFNGHVYDVDSSLSMADQELVSCSPACRCNNRTTPASFVCAHIDCPEFFGRDDSCVYQYTSRGCCSSNKVCGKEVEKLDVCYLDGEQYMEGQKMYPKDESCHTCHCQKGFDNSTVVGNPNCYEFTCGIEVHNSDSVMQGCIPIYFGTDRCCPISWRCPDDKDTVIVEGRLDVDEPVDPKMQCTFGKLKLNYGDSISSDDKCVSCKCTVPPMPHCIQSRDC |
| Aa-4525 | MNSKQVVFSLGLLLVVVGLSGVQGAALREKRQLNSLLTLSGEANARIENGTIFCDTLKCPPESFKCIIVKNNTRENLNQVEITRECLDPAGKPTAKTIEREANQFPGTHFESYAEIDKNGNIASFDNRGNSYNHSGSGDLHSYLYKQIDELHKQILDQLQGVSSSYRIP |
| Aa-4531 | MTESAGVKQLVGVADITENRNIWRMLVAEFLGTFFLVSIGIGSTMGWGDGYAPTMTQIAFTFGLVVATLAQAFGHVSGCHINPAVTIGLMITADISILKGAFYIVSQCVGAIAGAALIKAATPSDVIGGLGVTGIDPRLSPGQGVMIEALITFILVFVVHGVCDNRRSDIKGSAPLAIGLSITAGHLSAIKYTGASMNPARSFGPAVVMGNWTDQWVYWVGPIVGGILAGAVYRLFFKVRKGDEESYDF |
| Aa-45626 | MACSKTFQGSAVLLRNSAVFNGCVSKRASSWWGAIQMGPPDVILGVTEAYKKDTNPKKINLGVGAYRDDSGKPFVLPSVHKAEKRLMDKQLDKEYSPISGTADFCKHSITLALGEDSQHVAAGQNATVQAISGTGALRVGGAFLNGFFPGTKDIYLPTPSWGNHGPIFRHSGLNVKAYRYYDPSTCGFDFKGALEDLSKIPERSIVLLHACAHNPTGVDPRPEQWAEMSAVIKKRNLFPFFDMAYQGFASGDVAKDALAVRAFLKDGHQIALAQSYAKNMGLYGE |
| Aa-4581 | MRYVAAYLLAVLGGKRPSPSNADIEKILSSVGIEADSTRXXXLKGKSVEELIASGREKLSSMPAGGAAPAAGAGAAAGGAAAAPAEEKKEEKKEESESEDDDMGFGLFE |
| Aa-45866 | LWKSSKAFKTDEATVGGLWEKTKKSIFLLTERTKTLGLCDQGITTYFSSNVTREDTELISEWMKENKFEAYICRTFKTLEDGKTVYNIKLASAEEGEKEGLTKDPVEYKDCLFKITRGDYKELMGKVAEYLREANKHSANDNQKQMIESYVQSFTEGSLDAHKTGSRFWIKDKGPVIETYIGFIETYRDPVGQRGEFEGFVAMVNKEMSAKFGTLVSNAENFIKFLPWGEDYEKDNYLKPDFTSLDILTFAGSGVPCGINIPNYDEIRQDEGFKNVSLGNVLANTNKTDSIPFLSEEDQALLKRYKAAAFEVQVGLHELLGHGSGKLLRINEKGEFNFDREKVKNPLTGEAVTSWYEPGETYDSKFKSLGSSYEECRAEAVGLYLSLNRDILKIFGHTDEKEIEDIIYVNWLLLVWGGVGVAMELYNPTQKAWLQAHYQARFVIMKVLLEAGEGLVSVEETEPGKNLLLKFDRSKVETVGKKAIEEFLLKLQYYKSTGDIVGATKMYNHYSEVSEEGPYPWAKWRDIVLLHKKPRLIFVQANTELDAEGAVQLKTYESNFEGYIKSWTDRFPDVEIDSVLERIYENDLKYFQDLV |
| Aa-46646 | MCGSVRSSSTVTMKAVAFCLAVVTLLGFHTFPVDGINQDDIPMNQRIPAIFRSSRQTIGGTMNYDLSFEKMVNLMKGFMKPKAGQGGGEAAKQPASATEAPVRKETEKSAAKVMKESEEESSLGEDDAGKARAAKPAPEAEPSKGGSVASEALESGSEEKRGNGLPPMNGGKSSEKDED |
| Aa-4681 | DQECGGTTCEKLPKAIRSECHSFVDLYGDAVIALLIQSMDPREICPQLHMCPAAREDIEIFAPAQIDVTIDANAGKDKPTCPLCLFAVTQLEETIKNDRTKENIKQALSKLCSHLSPKLKMECTDFVDTYSAELVEMLVSDFTPQEICVYLKLCVDQRPDLSLLNMEFDHDFRQQQRTHYDIETNEIADNTVNGQITVDHQATIASPECLVCQEMVKEVEKRVKNKKSKEQIKEALEHACDRLKKYKTKCERYIDQHSDQIIDLLMKQLSPKEICHSLGFCIAKEFDELEVDEALLDYVVEPGVMVEPPKELFTPVEETTVQGQPPQCAMCEFVMVKLESELADKKTEEDIENAVRSVCSKLPNTVTKQCDHLIDQYGKFIIKFLATLPPKEICTRLALCEKQLAKLEESNLEIIECAVCQGAVKTVDDILGNKKIDYDIVQDVEKICNTVPAKYFEKCRKMIEVYGVSMVRQLQKYVEREQVCVNMGMCSNPTGYVKFEDEVAQVDHVEKKEVHLVGLDECTWGPGHWCATEENAQKCNASEFCAKKKLGKWQD |
| Aa-4703 | MALSDADVQKQIKHMMAFIEQEANEKAEEIDAKAEEEFNIEKGRLVQQQRLKIMEYYEKKEKQVELQKKIQSSNMLNQARLKVLKVREDHVGSVLEECRRRLGEVTRDPARYGEILSALITQGLLQLMEANVVVRGRQADAQLIQNILPSAVEAYKSTSGKDVVVTLDTDFYLPADATGGVELVTQSSRIKVSNTLESRLELIAQQLIPEIRNALFGRNLNRKFTD |
| Aa-47229 | MEVTPIAYLIILLNCCKWATATIDERIVGGYFDRIENVPYTVSLNTKLFGHFCGGSLVANVWVVTAAHCLWGKKPSDIFVRAGSTYKNRGGEIRKAKKIILHPLYKRIVDVPLDYDIALVQLNKPLSNNSDFIEYISISNPLEKIPGDPQCIVSGWGITKSEVGQFQLLKSATVRIVKHEICQKTLYQKIISKNMVCAGGQEDDACQGDSGGPMACRGKLHGVVSWGEGCATLGKPGVYAYLPELWDFVAAHIYMDTDESEDINRMLPKIKLYGKK |
| Aa-47530 | MKTILLAIVVVLVATGDGAPRQIQELKDFPLNEVVPNTFSQRGFNGSWLSDKEFIYRNTNGDYVKYNVESQTETVVLEASRLEQWRGASVTFIKPDVDKVLIRYASRTVFRHSTLSKFVVLDINSGNTYDVANAEDISVCTVSPNGQSLAYVKDNNVYYRAVILSTSEISLTTDGVPGVIYNGAPDWVYEEEVFGTDSTLWFSADGSHLAMASFDDTDVKEFSYHMYGDPHDPEFQYPEEYKLRYPKVNTTNPTVHLRVMNLADTTKWHELPAPEVTVSADHILGTLNWIGNDLGAIWMNRRQNSATYQRCNVETQVCRQMVAVNEQNGWYVLYTPRCTKSGDRCFFLGNANGWQRIWDLNGEDTITYKSPEEYTVTSINGYDESKDNLYYTAVPASAPQTRHVYRNGDCLTCSLKDKFSNNAPATTLAISFSXXXSYFAATCVGPTHSYTNLPTGQQLIADWETNEQLRTKLTPYKETQVRFLKVPVHGGFEASVRLYLPPEIDFENPANNKETYPMIVQVYGGPNSARVIDTFTVGFGNYLTTTKKTIYCQIDGRGSANQGYDFLFSVNNRLGTVEVEDQIAVTLQLQETYGFIDRNRTGIWGWSYGGYVTSMALEKDNGSVFKCGISVAPVTSWMFYDSIYTERYMGLPQVQDNEAGYEMADVSRYVAGMKNHMFLLIHGNADDNVHYQNSMVFVRALVDEDVEFEQMSYPDEDHGLGGVTQHLYHTMDNFWNQCFA |
| Aa-47561 | MAKPQGAAFWMGAQTLPIPMTMFRENREKVIGELRKVRTFDGTALIVLQGGDNISHYDTDVDYVFRQESYFMYLFGVTEPGCYGTVDVASGSTTLFVPRLPSEYAVWMSPLLSLDDFKQKYEVDAVYYADEIDVKLGELNPSVLLTLCGPNADSGLEAKPANFKGIEKFVVDSDILFPVIAECRVIKSPAEVEVLRYVAKVSSDAHKRVMKNIRPGWHEYQGEAEFLHHAYSVGGCRHVSYTCICGAGTNSAILHYGHAGSPNDRLIKNGEMCLFDMGANYNGYAADITCSFPVNGKFTDDQKLIYNAVLAARDAVCGAAKEGVSWVDMHLLANRVMLGEMKKGGLLQGEVDEMMSAGLNGIFQPHGLGHLIGLDVHDVGGYLSHCPERPTQPGANRLRMARTLMAGMYVTIEPGCYFIEPLLNKAFADPNLSKFLVKEKLDRFRNFGGVRIEDDVLITKTGIDNFTLVPRTVEEIEAWMAQ |
| Aa-47604 | GQLLRISEYPQQFTKQLFNLREYTSQLFRMGNSGSMHGLPGYDEGFHHPSYQHQYQRYQEMMRANGHHGAPPGWLESYQRDLRNGGGGGLIGGGGGHQTFDNRRSHEGLGHRESTSSGPSLPPPMKVLPDIPGRVAKLRPTNNGNILHSGGTISKNNNLQRSKSISSPTYQQHQQQAFDESEETGVMTLLPPRLAMQRSRTQMNMMTPGRRDFDESPSRGLGPRGYATNQRKQSYEHGTLNKKRFGSEPDLRISTSSNELQDDSSGGGSGGQAGHRNTKPVQSKIIKGKNKKKAPVPPPLEKREREPEMMEKQRIIAYSPLRKTNSDASSTQPSSDSNSNNPTRKLRLFKTRAETKKNLNIAKLPEASDAKYSAKNGHKLPLAPSNSSPYKLMSRKDPEFDSGTLDK |
| Aa-47669 | TVAMRGYSWFRRLSCWSSICSMCDEKFHKIPWLENRDVLLCSVDAALHCWRRRWRQRTNVRSPLPSSCYFVSVYCRQWSAYGYDAFLKYKAVSAGEIAQGSRTVQQQQQQQTISTVTTY |
| Aa-49087 | GNKFGEPVVSGFTISFGMVTDDKERLEYVKPILFSGGIGTMNSKQINKLDPEPGMILTKLGGPVYRIGVGGGAASSVEIQGDNDSELDFNAVQRGDAEMENKLNRVVRACIEMGDKNPILAIHDQGAGGNGNVLKELVEPGCAGAVIFSKEFTLGDPTITIMELWGAEYQENNAVLIAPEHRQLLLEICERERCPVSFVGYVTGNGYVTLVDEKFDSSKYSKRDNPKNFANLPFDMHLDNVLGKMPRKEFKLQRKPMKLNEFDLSNVNLMEALNRVLSSVTVGSKRYLTNKVDRCVTGLIAQQQCVGPLHTPLADYSISAVSHFGFEGIASSIGTQPIKGLLNSAAGARMSVAEAVSNLVFAGITQLADVKCSGNWMWAAKLNGEGAKLVDACRAMCDFMGKLHIAVDGGKDSLSMAARVNTETVKSPGTLVISTYAPCPDIRVKVIPDLKAASMGLETTLLYVSIETKFRLGGSVFAQCYGQLGMHSPDVQQTDVLLQAFNTTQQLLKAGALLSGHDVSDGGLLTCVLEMAFAGLTGVKLDLTELYKKFGKQFESLEEAAKHVCFAEECGWVLEVDPKHVANVLDAYRLAGVPCMAIGHAYKTDVHQNGSASVTMNGSQIVKASVISLFKQWERTSFEIEKLQADEKCAVQEYESYDYRTGPTYSCSINPDVLYASKAFGTKPRVAVIREEGTNGDREMCAALYEANFEVHDVTMSDLLTRKTCLDNYRGVVFPGGFSYADTLGSAKGWAACILYSDVLSPQFKHFKARADTFSLGVCNGCQLMGLIGWVSTEDQSSGTDVPDVALLPNKSNRFECRWSTLKIGENNSIMLRKLKGSVLGCWVAHGEGRFSFKSKPVLDKLKKNNCIAMQYVNDQGEATEVYPMNPNGSVEGIAGVCSLDGRHLAVMPHPERCAKMWQWPYVSKGFDFQTSPWHSMFAEAYNWCVEK |
| Aa-49088 | MVIVRFFSAVDEDQRQRILHRFQKVNAHVISLRVEKCYHVQNSKYSEFPRDVEKLLRWILKGPQQEDNLSAVSTLAKEKENEELVEIGPRFNFSTADSTNSVSICHNVGLQFIDRIETSLRYLIGFDSSRFSEKDLAPLMDVICDRMTQCRYTKANIPQKDFYENFALSDEKWYTVPVIERGVEALKEVDKKLGLAFDDWDLQYYTNLFRNVLKRNPTNVELFDCAQCNSEHSRHWFFKGRMIVDGVEEEKSLIDMIIDTQKFSNPNNTVKFSDNSSAIKGFSHQILTASTHAVPGPLEVKPINSDLIFTAETHNMPTALSPFSGATTGTGGRIRDVQSIGRGGLPICGTVGYCVGMLNIPGYELPYEKQLEYPPSFSKPLKILIDASNGASDYGNKFGEPVVSGFTISFGMVTNDKERLEYVKPILFSGGIGTMNSKQINKLDPEPGMILTKLGGPVYRIGVGGGAASSVEIQGDNDSELDFNAVQRGDAEMENKLNRVVRACIEMGDKNPILAIHDQGAGGNGNVLKELVEPGCAGAVIFSKEFTLGDPTITIMELWGAEYQENNAVLIAPEHRQLLLEICERERCPVSFVGYVTGNGYVTLVDEKFDSSKYSKRDNPKNFANLPFDMHLDNVLGKMPRKEFKLQRKPMKLNEFDLSNVNLTEALNRVLSSITVGSKRYLTNKVDRCVTGLIAQQQCVGPLHTPLADYSISAVSHFGFEGIASSIGTQPIKGPLNSAAGARMSVAEAVSNLVFAGITQLADVKCSGNWMWAAKLNGEGAKLVDACRAMCDFMGKLHIAVDGGKDSLSMAARVNTETVKSPGTLVISTYAPCPDIRVKVTPDLKATSMGLETTLLYVSIETKFRLGGSVFAQCYGQLGMHSPDVQQTDVLLQAFNTTQKLLKSGALLSGHDVSDGGLLTCVLEMAFAGLTGIKLDLTELYKKFGKQFESLEEAAKHVCFAEECGWVLEVDPKHVANVLDAYRLAGVPCMAIGHAYKTDVHQNGSASVTMNGSQIVKASIISLFKQWERTSFEIEKLQADEKCAVQEYESYDYRTGPTYSCSINPDVLYASKVFGTKPRVAVIREEGTNGDREMCAALYEANFEVHDVTMSDLLTRKTCLDNYRGVVFPGGFSYADTLGSAKGWAACILYSDVLSPQFXXXXARADTFSLGVCNGCQLMGLIGWVSTEDQSSGTDVPDVALLPNKSNRFECRWSTLXXXXXXXXXXXXXXXSVLAVS |
| Aa-49090 | GHIRNVCAAGLVLGGYPTDQSHQLAAVADAQRERIGTCLEMLELWRKHIAVQDTRGPTLGRAQRVGVTEATWENNTPVVIQTSLPGQQIAHSDVMHFEVCLVESSAHLAIPVGAFFANHSHSRLRTKRLGSIQHIGIDAAAVRRSGPVVVGFVFLHGTLFVGLKLFDLKASSFPLFEQADDACFHDLRSVHGNRGRTVLMNVSFVRMANGHAGDSCQSIGVQDIGNVLGVYLKNPTAFFSETTLLYVSIETKFRLGGSVFAQCYGQLGMHSPDVQQTDVLLQAFNTTQKLLKAGALLSGHDVSDGGLLTCVLEMAFAGLTGIKLDLTELYKKFGKQFESLDEAAKHVCFAEECGWVLEVDPKYVANVLDAYRLAGVPCMAIGHAYKTDVHQNGSASVTMNGSQIVKASVISLFKQWERTSFEIEKLQADEKCAVQEYESYDYRTGPTYSCSINPDVLYASKAFGTKPRVAVIREEGTNGDREMCAALYEANFEVHDVTM |
| Aa-49591 | MAAVTEVGGIKLGKLLIEGKTKQVYDVPSMPGHSILLNKDRITAHNGVRAHDLEGKAQISNQTNAKVFGLLNQVGLKTAFVRMVSDNAFLARKCDMVPIEWVTRRLATGSYLKRNPGVKEGYRFSPPKQETFYKDDANDDPQWSEEQIVSAGFKVNGVLIGQDEVDIMRQTTILVFEVLERAWATRNCALIDMKIEFGVDADGQLLVADVIDSDSWRLWPSGDKRLMVDKQVYRNLASVTASDLDTVKRNFLWVSEQLDNLQPKNDHLVVVLMGSPSDKDHCEKIAKNCRELGLNVELRVTSAHKGTKTTLHIVNEYESVLSNLVFITVAGRSNGLGPVLSGNTNYPVINCPPVKPENVNLDVWSSLNLPSGLGCATVLYPEAAALNAAQILGLNNFLIWSKLRVKQLANFTSLIYADKSFRGVRKE |
| Aa-51219 | MKRKQIQISAGLLLLTFILAPAINADELPKEIKDLDIDKLREQLPEGLLPPELLNVTLPSLEDIQRIVKDKCSRVAGSDAAYQQAEQSGQKLNDCLQGLLDFSDLQNEIKKAKPTGDLDTVFNKYCRRRSTAIECINTFSNDVDVCLEEEERESKKVLVNIIQGLLNFVCHKDGDQIALFIAEEGPECFEEQKQPMIDCFNSTLRGYLDEPTPQASEGIPKLVMGKKQCDDMDNLRDCFVRVLEDCKQSTPANLVESLFKFVRRETPCANFTTPEHSKRGAADVSRASLHIILATWLLALLAKMFVQ |
| Aa-51263 | MAVASHSGFMLQTMLFMVNHPWNNSLHRTIHSGTTSTSFNEESRCYGVYGCFSLGYPWVDETRPMVYPRSPDQLNIRFPVYNKYAPYTPKYVDVDDPDGVQQLGIDRKGWLYVIAQGHITSGTEPWVQRLVNTLLQNDLHGTSSIITVDWRKASTLGYTQHASDVRVVGAITAHMIHMIYEETGMPNLDKVHLLGHSVGAHLCGYVGYHLQKDFGLQLGRITGMDPAEEMFFGADPIVRLDNSDAKFVDVIHGRAEIYQSIGHVDFYPNGGHDQPGCNESMRDHIYQNQDRSCDHMRSPDLFINAVRRNCSAVAIGCQSFEQFLAGDCFECNEDGHYCIDFGLNAWSSYRGLIENGVMTVPGQVRAFMITGEDESYCRNHFRVTLHVSDGEESLVHGGEIGKLAVEIIGKHRNHSGLMDLSKNPLYFEPGRSYSSVVAGKDVGIPKRVLLNWEYKKHPGYSRKNWQVTRTPRIYVDHILVQSLGHRAWQKFCPPNREPVKAVNEDYVRSPVNEFREEYCR |
| Aa-5134 | MRSLLLIAAVLVAGALSLEAGQRCEPGSTFKMDCNTCRCSADGMVVSCTRKFCLPELQGDDPKVQEPSGNAQSEAAAVEKEEEEVHTNGQVCTPNEVKMEDCNRCKCAANGIGWFCTRKACPPRERRAVQPKMECEPGTTRCRWQRTCSGCSPPSSPHWGCTPARWCALPLPPFRQRQPPTVRYR |
| Aa-5198 | CCSKQDVQQGCYCPEDSVYQQIQQYYKQHGASINIKKIFQEDDIRFDKFSLKLNTPKDGEILLDYSKNRITDEAWNMLMDLAESRDVVKTRNDMFNGERINITENRAVLHIALRNRSNKPIMVDGKDVMPEVNAVLDHMKEFTEQVLNGVWRGYTNKKISDVVNIGIGGSDLGPLMVSEALKAYNTGIRSHFVSNVDGTHIAETLKKLDPETTLFIIASKTFTTQETITNATAAKTWFLERCGEQEHVAKHFVALSTNKEKVAAFGINTKNMFEFWDWVGGRYSLWSAIGLSISLAIGFDNFEKLLEGAHYMDNHFLSAPLNENAPVILALMGIWYSNFYGAETHALLPYDQYLHRFAAYFQQGDMESNGKGVTKSGKRVDFNTGPIVWGEPGTNGQHAFYQLIHQGTRLIPCDFIAPVFTHNPVENGGMHKILLANYLAQTEALMMGKTEEQARAELEKAGMSGEKLEQLLPHKVFTGNRPTNSILVKKITPFVLGALIAMYEHKIFTQGVIWDVNSFDQWGVELGKQLAKAIEVDLNDANKTISHDSSTNGLINFIKVHWEQEGN |
| Aa-5201 | MAASTGNSIEIGQSEQELVGSKKRNTKKYLLIGGGVAIVLAVVITLVVVFTGDDSSDNGDNSPIVSGGDPITLEDFLTGKLSARGFNGAWSPSGKVISRDDVGRVLAYDPATNETKTLLDEQHEDLLQGFKFDLSADERYLLVARGYSKIFRHSFLAVYDIVDLQNNRVIPINVNGERKALNVVEWSPVGNSFIFVFLNNLYYKASPDAQELQITSDGEASIYNGIPDWVYEEEVFSTNLATWFSADGQKLAFIRFNDTTTRLMKIPIYGPPGHPEFQYPHELALHYPKAGTPNPSVNLYQVDLVNPSVKTEIKPPAALVTPENDHIITSVGWASNNRLITIWKNRVQNHAIVTTCDENNSCNEIQDIRVDGGWLELFSAPVFNKDGSQFVIIKSQDQPDAGGYNHITMMSTQTRSSMAFTSGKYVVQEILLWEPETNLIFYAANTEEDSHVLHIFAVQGHSGAKPMCLTCTVGSSPKQSYFNAQMSKKGNYIVLEAKGPGVPWSEMFEWSFANDAVTLKPVKTLETNSELRNRLEGKSLPTVQYHEIDLDNGFTSKVMLLVPPGADLSGKTKYPLLVDVYGGPN |
| Aa-5202 | CNEIQDIRVDGGWLELFSAPVFNKDGSQFVIIKSQDQPDAGGYNHITMMSTQTRSSMAFTSGKYVVQEILLWEPETNLIFYAANTEEDSHVLHIFAVQGHSGAKPMCLTCTVGSSPKQSYFNAQMSKKGNYIVLEAKGPGVPWSEMFEWSFANGAVTLKPVKTLETNSELRNRLEGKSLPTVQYHEIDLENGFTSKVMLLIPPGADLSGKTKYPLLVDVYGGPNSYSVTSSWSIGWGHHMSSNRSVIYAKIDGRGSGLRGDKLLFQIYRKLGTFEIEDQITTAKKLSEKLPFVDPARAAIWGWSYGGYASAMALAKDSNRVFKCAVSVAPVTDWTFYDSIYTERYMGLPTATDNKQGYEQSRLTAMYEKFRDRKYMLVHGTFDDNVHYQQAMQLARALETHDIMFKQVSYPDEDHSLAGVRPHLYHTLGRFFSECFDLRD |
| Aa-52703 | MSALTKGVFIVAAKRTAFGTFGGAFKNTNATQLQTVAAKAALDAAGLKPDQVDSVNIGQVLVLSSPDGAFLPRHVSLHCGIPIDRPALGVNRLCGSGFQSIVNGAQDILLGAAKVSLTGGVDNMSQTPYTVRGTRFGVPLGTNPALEDALWVGLSDSFCKLPMALTAENLAEKYKIPREKVDEFALRSQQLWKKANDEGVFKTEITPFKLKVKGKEVDFAVDEHPRPQTTLEGLNKLPSLFKKGGAVTAGTASGICDGAAAVVLASEEAVKEYNLTPLARLVAYSTVGVPPEIMGIGPVPAIQNVLKVAGVKKDDVDLFEINEAFGVQAMSCVQELGIDMNKFNLNGGAIALGHPLGASGSRITGHLVHELKRKNLKRAVGSACIGGGQGIALLVESV |
| Aa-53503 | MAKLGLLFIVIFCIIQFTLAARLRRDAFDEAVDTVKKGISDTFTKENVDNFLGKLTELGDTIKAKASEIGQTLQDKAQEAMKKE |
| Aa-5373 | GDEKGFMFSGRVLIRMLCDEDAMNITLHSKNLTIGEKDIKLLELSDSGSKTMDIKRVQYITDNDYVVFHTSESMKKGYRYDITIPFEGVLGTGLLGYYRSSYVDQKSQKKIWLSVTQFEPTYARQAFPCFDEPEMKATFDISLGHHKQLTALSNMPMNRSEPMTAITDWVMDHFGTTVPMSTYLVAYTVNDFEYRESMTKMDGDVVFKIWARRDAIDQVDYARDVGPRVTRFYEEYFAEKFPLPKIDMIAIPDFSAGAMENWGLITYRETALLYHPNISTANNKHRVASVIAHELAHQWFGNLVTMKWWTDLWLNEGFATYVASLGVEYLHPEWHSLEEESVDNTLGIFKFDALTSSHPVSVEIGHPNQISQIFDAISYEKGSTVIRMMHLFLGEETFRNGVRRYLKLHKYANAEQNDLWAALTEEARVNKVLPDDVDVKTVMESWTLQTGYPVITVTRNYESSTAEITQVRFLSDREKQANATDYCWWVPLTFVTSENPNFEDTRAKDWMMCGAGKLRKGPIKQLQKMPPADQWVLFNVQLAGLYKVRYDKTNYKLLIKQLNSEQYFTISLANRAQLIDDAMDLAWTGEQQYGIAFAMINYLRQEVEYIPWKSALSNLNAINRLLKRTPIYGVFRSYIQFIVEPIYEKLQIFSEDRAVSQRLDATKQLVQIAAWACKFDVGDCVERSVALFAKWMAVQDPELSNPVPRDLRSVVYCNAMRNGKETEWNFLWDRVGQLRILDGHPLREQSYRAFNAVSDVEFTGPGSDLNQLFGGVQPLTDGSIFAEDLQLLVDGFNDELDVASEHSIDRGALEQSVDRVEVREGRLPGDVFHLLAKVVDHGEGDAVLLLAGPGQIHGVIDQLSTVRQADRRVLFAVQLLDQ |
| Aa-5381 | LVELQVSTDSVAEGFLAEEEVHHSNNSRSLLVRDGVEYLHPEWHSLEEESVDNTLGIFKFDALTSSHPVSVEIGHPNQISQIFDAISYEKGSTVIRMMHLFLGEETFRNGVRRYLKLHKYANAEQNDLWAALTEEARINKVLPDDVDVKTVMESWTLQTGYPVITVTRNYESSTAEITQVRFLSDREQQANATDYCWWVPLTFVTSENPNFEDTRAKDWMMCGAGKLRKGPLKQLQKMPPADQWVLFNVQLAGLYKVRYDKTNYKLLIKQLNSEQYSTISLANRAQLIDDAMDLAWTGEQQYGIAFAMINYLRQEVEYIPWKSALSNLNAINRLLKRTPIYGVFRSYIQFIVEPIYDKLQIFSEDRTVSQRLDATKQLVQIAAWACKFDVGDCVERSVALFAKWMAVQDPELSNPVPRDLRSVVYCNAMRNGKETEWNFLWQRYLKSNVGSEKVMIIGALSCTREVWLVERFLLWSLNSTSGVRKQDTTIVFGGVAKSDVGFHLAKSFFLENVEEIYNYLSPDTSRVSRFIKPLAEQMSSMKELQELKDLIESKRTVFEKATQGVKQALETVEINLQWKSYGYTQMTRFLPLLSYRSGNLDVMELLD |
| Aa-5389 | MGLSNALFLVLSALLIDTGYSSVSAPFCQCPCENRISISKDWFNCITIYSMKTKGYVTADEHYPFLQYYERYVFATNQNKLWGTAKWKVEYKEGNNGTYGLKNLYVKEWLHAGRNDQARDSFRRYLLTKIRGDPNIPPRDGYWQFIPDPKIGKDVYRIRNTFTGEYLFVDDEQHIQRYDYNRLYLWRLTGNYQSTDNRHWFKIAKCK |
| Aa-54049 | MASGKIAILSVSDKSGLLDFAKGLNQLGLKLVASGGTAKAVRDTGIPVRDVSDITSAPEMLGGRVKTLHPAVHGGILARETPSDLNDMKRQNFEFVQVVVCNLYPFGLTISKPDVTVEDAVENIDIGGVTLLRAAAKNHKRVTVLCDPSDYGKVLDEIKQFGDTTEATRQVLALKAFTHTAEYDNLISDYFRKQYSAGVSQLNLRYGMNPHQKPAQIFTTLEKLPLKVVNASPGFINLCDALNGWQLVRELKKALGLPAATSFKHVSPAGAAVGVPLTMDQAKLCMVDDLFDSLTPLATAYARARGADRMSSFGDFVALSDTCDLATAKIISREVSDGIIAPGYTEEALELLKKKKNGGYCVLQIDPTYEPSPVERKTLFGLQMEQRRNDADINKALFTNVVTKNKNLTDGALRDLIVATIALKYTQSNSVCYAKDGQVVGIGAGQQSRIHCTRLAGDKADNWWLRQHPRVTSMQFKKGVKRAEISNAIDNYVNGTVGKDMPLAQFESMYEKVPEFLTEADKLNWAKQLTGVSLGSDAFFPFRDNVDRARLSGVSYIASPSGSTNDAGVIEACNDHGIVMVHTNLRLFHH |
| Aa-5517 | MENQIEPDVEVTFNFNFANAPPSNDDQKPVKGTVPTAASLVDEDNRVISAKDVPTPKSKLQELESKAQKAQEAFEKEEKARKELEALNSKLLAEKTALLDSLSGEKGALQDFQEKTAKLTAQKNDLENQLRDTQERLSQEEDARNQLMQTKKKLEQEMNGQKKDAEDLELQIQKIEQDKASKDHQIRNLNDEIAHQDELINKLNKEKKMQGEVNQKTAEELQAAEDKVNHLNKVKAKLEQTLDELEDSLEREKKLRGDVEKAKRKVEGDLKLTQEAVADLERNKKELEQTVMRKDKEISALSAKLEDEQSLVGKTQKQIKELQGRIEELEEEVEAERQARAKAEKQRADLARELEELGERLEEAGGATSAQIELNKKREAELAKLRRDLEESNIQHEGTLANLRKKHNDAVAEMAEQVDQLNKLKTKAEKERSQYYAEMNDARLSLLSWSTCSAISATASLCFLRRLAKVPSCWMLDSSKSRRSLASSASRFLFSSIWAEVAPPASSRRSPSSSSSRARSARCFSALARA |
| Aa-5541 | PKCQTWKSQCNEHNRFSVCGSSMFGIWVIGKLFPNPSENQVIKLSESSESLEGTRADSKTCTAPHEIYVDRLLMCPLNCQSWTEWIMGVDRAFGRCINPCHCEKGYIRRESEGPCIPIMECASVRNAVGLLREMIHGMYTGKIKYLD |
| Aa-5546 | MPGFMFNIDGGYLEGLCRGFKCGILKQADYLNLVQCETLEDLKLHLQGTDYGQFLANEPSPLAVSVIDDKLREKLVIEFQHMRNHAVEPLSTFLDFITYSYMIDNIILLITGTLHQRPISELIPKCHPLGSFEQMEAIHVAATPAELYNAVLVDTPLAPFFVDCISEQDLDEMNIEIIRNTLYKAYLEAFYEFCKNIGGTTADVMCEILAFEADRRAIIITINSFGTELSKDDRAKLYPRCGRMNPDGLAALARADDYEQVKAVAEYYAEYAALFDGSGNNPGDKTLEDKFYEHEVKLNVYAFMQQFHFGVFYSYLKLKEQECRNIVWIAECVAQKHRAKIDNYIPIF |
| Aa-55677 | MASETAALPEVLKNLFSHIDANKTKYISALSEAVAIKSVSAWPDSRPEIFRMVNWVADRLKALGATVELADVGKQTFPDGRVLDLPNVILGTLGNDPAKKTVVLYGHLDVQPAILEDGWDTEPFVLTEKDGKLFGRGASDDKGPVLGWIHAIEAYQAINEPLPVNLKFVFEGMEESGSEGLDELLYKRQNDFLANVDFVCISDNYWLGTTKPCITYGLRGICYFDVEVGCSGKDLHSGVFGGTVYEAMNDLVYLLGTLADKEGKILIPNLYKEVAPLLQNEQEMYDAIDFDVSEYRDQLGARRLMHNEDKSKILMHRWRQPSLSIHGVEGAFYEPGQKTVIPKKVIGKFSIRIVPDQTPELVEKYVTEYLTTKWAERGSPNKFAVRMAHGGKPWTEDPNHPHYQAASVATKYVYNVDPDMTREGGSIPVTLTLQQTTGKNVLLLPMGASDDGAHSQNEKIDVRNYIEXXXXXXXXXXXXXXXXXXXXXXKLLGAYLYEVAKIK |
| Aa-56317 | MIRVFVAVFSAITLATVAVSAIESQLFSSEFIWDFFKSSFDERRNLATSPFSIRLGMTMLANSVTDGYTLKQMTDKLHLPSSIARASEQNRRKLMVLQKDKHFAYATKLIVLGTESLNPKFLAAMHNFDTPAERHPLSNLKSIPTLANRWAKNMTSGMVSTVLMDNELLPDTRMILLSATAFASKWENQFNVNVSKVEMFSAYTTGKLYMTNFMNLEHTLLPVAMNYDLKMKAIELPFEKGSDYSFMIIMPLDRDGNMTEMVGRLNHKTFTKLYDSLVPMRISVKMPRFKVSTGVNVNSVLKKLHLTAPFQWSTFQIFRQEKLTLDKVKQSVTVQVDEQGVRAAAVDAYVMVTRSAPITFQADRPFVFAILKKSVHFPLFVGHYAYPANSLPIKP |
| Aa-5646 | MQFQTIVSAVIAQLLLASLVADSAKLPFNKYVIMDDKVTFFEAWRSCQYYGLQLASVTSSEDNQQLSKLMNRSARGNDTFWLAGTDVGREGKWVWITTNKLVLHFSNWGSISPLFAEVNDCMAIGSFTEDRTLWDDIPCSEAHKYVCQKV |
| Aa-57652 | MPFPIFKECNVNLQDHLAPLEPLFIKNGQLWAPDGPELTWEEQESTTVACAKTKLVNVNSNTASLTCVSGQDFIVNGTPVNSTDLQCSGRMTGEVEETGETCGSSGGTLLKLGFNVEEIGFMTYIESCYDREEASVIYTRHTIPGRAIEHSIKESYRPSFKVAGASSHVNPATSYTQQAQLNRLTELLGSEEQAKKFLQGGSYYLARGHLAPDADGVYRSWQWATYFYVNVAPQWQVVNAGNWLVVENLSRSKAAQLQEDVVVYDGVHDVLLLPHVDGQSIPITLEAGGIRAPKWYWKIIVSPATSAGVAFVTNNDPFRTSLPVNEFLCEDVCSQYGWSDERFQDFTRGYTYCCAVADLQTAIEDIPRDLQVNHVLQK |
| Aa-5799 | EARCLCAPSICVCIRKKESPQKMSGKKADPYGFAKDFLAGGISAAVSKTAVAPIERVKLLLQVQAASKQIAADKQYKGIVDCFVRIPKEQGFGAFWRGNLANVIRYFPTQALNFAFKDVYKQVFLGGVDKNTQFWRYFLGNLGSGGAAGATSLCFVYPLDFARTRLGADVGRAGAEREYNGLIDCL |
| Aa-5809 | MGLGGRMDCCGQCVKYSMFVSNFIIFIGGAIVFGLGVWTVVDKNFINELLGTNLFSGAVYVLIATSALVCLLSFFGCIGAAKEYKCMLLTYFILIFLIFVTMLIGGILGYVFREKVSQTMQQEMHSSMTFYGAYGKRSITQAWDVTQERLKCCGVKHYNDWRGSIPQSCCQKTFTDYKPCQDHPTPENIYVQGCLDITSNLIRDNAAIIGASGIIVAILLIFGMIFSCSLFRMIE |
| Aa-6192 | MGSLSLLLVVLYATCHLRTGSAQITTFIVPEHKANWFDAAKYCYEKGWMLAAIMGADEERRVTAFAQRNSPTGWLNPRFWVAENDQEEDAEFCPQITLESYFEGENDCMEMLYFVCESLE |
| Aa-6278 | MKHIETTLVLCTLFITPILSQDEVNAEEIGNHFEGDMILNPEQLMAVKEESRNVLIASKYKWPHNTVRYRIDIEKFDPSQIEYIRKAMDTIESVSCIKFVEAGQMAKKYVNIVFEKPGCYAIVGYQAKPQRLNLTPARVGFKCFRIGTIIHELLHALGFVHQQSAADRDKYVKILWKNIEPERKHNFKKYKYSEVSDFNVKYDYGSVMHYPEKSFSKNGEPTILPKEPNVTIGQRVKLSEGDILKLNRLYKCKKKSSG |
| Aa-6300 | MRNLPAIVLLLVALSARTIHGQCLAQDDNIQHTKADNPLSRNRLYKGESIFTLKLLEAINTATPTENVFFSPYSLYHVLLLAYFGARTETEKMLRTGLELHWTEDKPVVWQAYNIGKKSLAARFGQSADIQFTSVDKLFFGKQVAIRECMEDKFFEEIEKLDFEKDADGQRLYINNWVENVTQGEIKDLLIPGSITKQTKLAIANAAYFKGTWQTEFKPDQTNKEIFYVSSERQAFVDMMHVLGTFNHAANEKLGCHVLEMPYKGQDESTRISMFVFLPPAAPNSLDKVLARLTSDTGILSEIVNEGIPRMVDVKFPKFSIEKTVELKPVLERLGLGKMFENGADFSAFSDKEQIGFDEILQKSKIEVNEKGSTAASSTVLFSFRSSRPVEPAMFHCNHAFVFLIYDYGTQAVLFNGVYRQPE |
| Aa-6408 | MFAVCILLIALFNFPNYVVPGKIVGGYVDTIRNVPYTVSLQLIYDGHFCGGSLISPDWIVTAAHCVHGRSPSDLTVRIGSSYRDSGGVLRDVRQIIVPEQYNPTASFDFDVALLNLMQRVSSSYETVDFIRMYEANFPTQKGMKCLTSGWGATKNPNQSSGRIKSAMLDIVDIDMCRYALYPSPVTDKMICAGGQNDDACQGDSGGPLVCAGRLAGIVSWGRGCGVVGTPGVYTFLPKVRMWIYEKTGV |
| Aa-6435 | MKLLAFVHLIILLTYCKFVAPKKGAYVNKIKNVPYTVSLQEKPFGHFCTGALVSKRWVVTAAQCVWGKKPSDIFIRAASSYKNKGGKIRKAIKIIIHPLFKRIEDVPFDYDVALVKLKKKLSDHSVYIGYIPVSSPYVMPALIGSDCLVSGWVISNKTDKQHQRLKSATVEIVGDLANRCQDKVSPKIVTRNMVCIGAQKSDACLGDLGNPTVCSGLLVGVSSWGERCATPEQPGVNTFIPEMWDFIASHVDVDTSYKANSNIL |
| Aa-66467 | QLVKAAIDAGYRHIDTAYLYRNEKEVGQAIREKIAEGVIKREDIFVTTKXXXXXXXXXXXXXXVILQLWNGYHDPAHVEESFNRSMSNLDIGYIDLYLMHTPMSYQFVSWEPENPDIPSVLTPTEVDYVDTWRAMEKLLETGKLKSIGVSNFNSEQVARIVKECNVKPVTNQVECNPGLNQRKLTEFCKKLGVTLTAYSPLGRPNYYEKDPKNTPKPALDDPRVLEIGKKYNKTPGQVILRYLVDIGTIPVPKSSNLERLRQNIDIFDFKLTDAEIKIMDGFNTGKRTVPFALSATHKYYPFNIEF |
| Aa-6693 | MTATKEYLAESELQKPNGRFGTRHYVVFMLFLGMANAYVMRTNMSVAIVAMVNQTAIEHEAEVFDDECPDTDYGEPADPADHKDGEFLWSTSLQGYILSSFFYGYVITQIPFGLLAKKYGAMKFLGWGMLINSVFAFLVPIAARQGGAPWLIVVRFIQGLGEGPIVPCTHAMLAKWIPPNERSRVGSIVYSGAQFGTVISMPLSGLLADHGFDGGWPSIFYVFGIIGTVWSVAFLWTCHEDPITHPSIAEDERKYIQQSLWGKAGVNIPPIPWKSISRSLPFYAILLAHLGQNYGYETLMTELPTYMKQVLRFSIKANGTLSALPYLAMWIFSIGVGWVADWMLTSGRFTHTLTRKLSNSIGQYGPAIALIIASYTGCNRALTVAILTIGVGFNGGIYAGFKINHLDLTPRYAGILMAFTNCSANLAGLLAPIAAGNIIEGKPTIAQWRIVFVIAACVYIFTATFYNIFASGTRQPWDNPDNDEPQKPVSIEAPAYENGHSNLANGGTTATMYRANNAAEQRQ |
| Aa-6845 | MPFKSIKARQIFDSRGNPTVEVDLVTDLGLFRAAVPSGASTGVHEALELRDNVKADWHGKGVLKAVENINKTIAPAVLNSGLCVTQQKELDEMMLKLDGTENKSKLGANAILGVSLAVCKAGAAKKGIPLYKHIAELAGNGDIILPVPAFNVINGGSHAGNKLAMQEFMILPTGASSFTEAMKIGSEVYHHLKNVIKAKFGLDATAVGDEGGFAPNILENKEALNLIQDAIAKAGYTGKVEIGMDVAASEFHKDGKYDLDFKNPNSDKSAWLTPDALEGMYQGFIKDFPIVSIEDPFDQDHWDAWAKMTANTSIQIVGDDLTVTNPKRIATAVEKKACNCLLLKVNQIGTVTESINAHLLAXXXXXXTMVSHRSGETEDTFIADLVVGLSTGQIKTGAPCRSERLAKYNQILRIEEELGSAAKFAGKSFRHPQ |
| Aa-69884 | MATRGFSVRTTLGYGFGVLLVIALSAATASVNNGVSNGTPANQSDRAAAVPAGHHIKDDDTFCEDGLLLKVWEPQDNLTTGDRFARGMVYFFALLYLFIGVSIVSDRFMAAIEVITSKEKEVRVKKPGGEEQIVVVRVWNETVANLTLMALGSSAPEILLSIIEIVAKNFNAGDLGPGTIVGSAAYNLFVIIAICVLVIPDGEVRKIKHLRVFFVTATWSVFAYIWLYLILAQITPGRVDVWEGLLTFMF |
| Aa-7100 | MVRISLQLLILGLTLLTITSTGDGAAPPKKKPKQPPTVQDELNRLYKLLSKLSRHTNRAIKETICIVPMVREPVCGSDGQTYDNRWLVECTDLLRKGGAAPKLTVAKKGEC |
| Aa-71480 | KVLSQGTRVEVDASQFHIPPVFGWLSKTGNLSAQEMLHTFNCGVGMALVVAKQHVQTVLEKLQSQRATLIGRVLNSSVDEPKVTVLNFVESLKTCQLTSCLPKKRIAVLISGSGSNLQALXXATRDTTYGIRGEIVFVLANKDNIYGLERAAKAGVPSKVILHKQFPSRDQFDAAMSEELERQKIDLICLAGFMRILSEEFVKKWKGRLINIHPALLPKHKGIHAQRQALEAGDSESGCTVHYVDEGVDTGAIILQERVPVLKDDTEETLTERIHRAEHVAFPKALRLVANGLISLNKDGKVQLH |
| Aa-71774 | MTAWRAAGLNYINYSNIAARLVRKALKPEQRAQAVRRDESHIKFTKWVNGKPENEKL |
| Aa-72237 | HTKTLEFNCRFGDPETQVILPLLQSDLFEVMEACCDDRLESVELKFRQDVHAVGVVMASKGYPETSTKGCVIKGLESIEKRPDHLVFHSGVGRNADGEFVTNGGRVLINVVLQPSLRAAAALASAACFDVKFDGSQYRTDISQKALKYSQLTYKGCGVDREAESDLLKRIEPLARGTDRPGTLRSFKGFGGLLRMKEVTYEDGTGKHVTYKDPVLVQGTDGVGTKLKIAEAMNYWDTIGIDLVAMCVNDVLCAGAEPLAFLDYIACGKLDVPTAALIVKGISEGCRETHCALLGGETAEMPSMYEPGKYDLGGYCVGVVEHDAILPRVDDIRSGDLLIGLPSSGVHSNGFSLVNKILSANGYKLTDVAPFSKHKLSFGMELLKPTTLYIKSVLPVLRKGQVKALAHITGGGLVENIPRVLSADLAVEIDANNFKILPVFGWLAANGNVPDSEMLRTFNCGVGMVLVVPPNDKSWESLKPYGGAVIGKVLNRKSSSTPQVVVNNFSQAIAEASKPFKGTTAKVAITYKDSGVDITAGDNLVTKIKPFAKATTRKGCIGGLGGFGGLFRLKEAGSFKDPVLVLTTDGVGTKLKIAQQINQHGTIGIDLVAMGVNDGLCNAAEPMNFLDYYACGHLEVDVAAQVISGIAKGCTESGAALLGGETAEMPGMYEKGATTLQDLFWALPSTIIFCQELPA |
| Aa-74051 | MSATLKNVLVVGGGGREHAMCWKLAQSPKVAKLYALPGSPGIGQLDKVTLVSDISVKNLDAIVAWCKSHAIDLVAVGPEDPLAEGIGDKLQSAGIKCFGPSLKGAQIEADKNWSKDFMHRHGIPTARYSSFTKADDAKAFIGSASFDALVVKASGLAAGKGVIVAENKNQACAAVDEILGDKKFGSAGDVVVVEEKLTGEEVSVLAFVDSKTVRVMLPAQDHKRLQNEDRGPNTGGMGAYCPCPIITQEELKMVTREVLQRAVDGLRKEGIKYNGVLYAGMMLTPSGPKTLEFNCRFGDPETQVILPLLQSDLFEVMEACCEDRLESVELKFRQDVHAVGVVMASKGYPETSTKGCVIKGLESIEKRPDHLVFHSGVGRNADGEFVTNGGRVLINVVLQPSLRAAAALASAACFDVKFDGSQYRTDIA |
| Aa-74092 | MATQYLLAVFTLLGLVAANDYFVYVEPSIYTTPLDCVRIRSANMQRLIYGTGYLYNTNRGRYIGVYNHHTIPHNSAFRVERIKEFKESLYYIRMINTQEFLFGGWTNTATHPDRRPVFSYVYSFNRTQGDVDVWMFEKVIKNDKFLGSWYIRNAYYGEYMIASKEHAQPPTQAGPAAQFYTIKLDRRPQPDLTIEHQFFLRIVEQANK |
| Aa-7717 | MAERFLPCKVLQPLTSFDRGYLKDNHYDTVCVIDGSQVPAXXANCFAARKSIDEAFDSEMCCFKSDALDCRVVYAPVGKLTDFDDVRRYSEAAGKALDRAIKAGAKQPVLVVPSSKDFSEADLVSVLGALAKLYVPLQLREDVPEKRQRFVQIGFFHPDKAKLELIVKEAKAFEAGLFVARDIGGGDPERMAPPRVEQYVSDGVYNSKLIASSVISDHNVLVKEYPLFAAVNRAAVSVPRHQGRLIFLEYKSGSNPKKTLILVGKGVTYDTGGADIKAGGVMAGMSRDKCGAAAVAGFMKVVEQMQPQDVHVIGVMCMVRNSVGEECYVSDEMITSRAGVRVRVGNTDAEGRMAMADALCQMKERVIAEKMPDAHLFTIATLTGHAVLAVGNHSIVMDNGPARASGHGQLLQEEGEKIGDPFEISILRKEDFEMHSGKCYGEDVLQANNLPSSRTCRGHQSPAAFMMLSTGLDKHGLKSERPIKYSHLDIAGSAGDIPDPPTGAPILALARAHLLR |
| Aa-7816 | TLAHRKTIRLQPKSTDTCLESFSCMSWKPSKVAPYWWSGKFTTQGEYVLTQHSAQSSLSTTAGAFIQWSVYTAIHTDHPLSFGLFDGILDKVVRAIQLKAVSDDELKLFWDATKKLLPSCFSVIRKLRKKTAGDKLVVKMLTDALNIIAKVSMLEPPEGTDLFPLQQYSWIRSNTADPNWDIREAVASAVASGAEDWFNSIKEGHFVESGLDEDRLQNLIKIIQLVRSDIQRSIEHYDKLFQEILHFQYTKELYITHELKLAELIKPCVEEICRALKRIDIPESNTTLMEYEDINMGTTLFELYLVLKRFSQLGPALSPGESGFAIDEYHNWFTAGVTHWLDISVYKALMRIHKAIELDKLQPVDETVKYSSSAVDTLAIFYQIKIFWQQLDWPDVEGAYIFVAKIVDDICRCCVFYADRMSTRVENLGLIENVYERKFEVTTEWCLAINNIDYIRQSLKPFAAELGVDDIITRLSNIQSSSEAERCAQTLRAVLENAVDTEKNKIIDLVEKLAKKMAPAMRRFLVEGAELLQQDSNSMDRLMMYMEDSLSTLNSELNEINFERVLDAIWGELTTILYDLIQSNLDKRRPPAFFANLRDTLHIMVQNFKSAENRESQNASDRDTLDHIERLLQLHGYETTDLIHQYYIDRLTEQEQAEEDAVFGMLTVQCFFKNNVLELEIMNARNLKPMDSNGTCDSFVRVHFIPEERFVGVTKPRTNTQSKTLFPLYDEKFVVTFTPEQRAIKDAVILFSVKDKDLFGMSNQYLAECYLSFNDIADISGESGKIEQKHLKLTRPHRLDTDCVRALEYRQGDKQAKDFIKKLRQKMG |
| Aa-7819 | RIKMKLKTIVFIALVLPCLAFGSSESISKSESSESSEGTRADSKTCTAPHEIYVPRLLMCPLNCQSWTEWIMGVDRAFGRFCINLCHCEKGYIRRESKGPCIPIMECASVRNAVGLLREMIHGMYT |
| Aa-7855 | MKLKTIVFIALALPCLAFGSSESVSKSESSESSEGTTSDSKTCTAPHEIYIRYSQKCTLNCHNWSEWIRDVDRTFDRCLNPCHCEDGYIRREKDGPCIPIMECEPFRNADEYEILDMDE |
| Aa-7982 | ECFVSRTESDPYRSSTDSHIFQTVLLVVTATATRAHNFCDCPCSRASELILQPYFPTGSACVRLKNTHEWKDMYLSDYMEDGGRMPYVDLTPYTGGPIPIHKHTAYWKLYYPWKNGTGQIPYVIQNILTLDFLVPTRQHTPNHYDRVVVAKPELTAKSLWTFFTGGGSVGLKIQNFVLKEYLQMDFVLRGKVRSDGRVFTKHEDTGMVETAWRTLGSPIGEWRMGIKKVSIFPMSNAICEINYLATVENGQNGVHETSTTESMNGDTEHKKEEKIPEKAAQLQQPPPAAEVTAE |
| Aa-7983 | VHVWHASSIFHGSLNHIFQAVLLVVTATATRAHNFCDCPCSRASELILQPYFPTGSACVRLKNTHEWKDMYLSDYMEDGGRMPYVDLTPYTGGPIPIHKHTAYWKLYYPWKNGTGQIPYVIQNILTLDFLVPTRQHTPNHYDRVVVAKPELTAKSLWTFFTGGGSVGLKIQNFVLKEYLQMDFVLRGKVRSDGRVFTNMKTPEWSKRLGEHWDPPSANGGWELRKCQFFQ |
| Aa-8246 | SEMLWRLSLLVLAGVVVALAGASNPQLEEDELAAAKYVTDLEREILARNYEATEASWAYESNLTEDNLKQRNEIQTRNANYFKEVARELRKFNYNAFKDADLKRKIKKLTDLGYAALSEDKFTQLLDAISSMSENYAMAKVCDYKDRTKCDLSLEPELTETLATSRDPEELKHYWIQWYDAAGAPTRENFQKYVELNKEAAELNGFTSGAEAWLDAYEDETFEEQVDAAIEEIRPLYEQIHAYVRHRLRERYGEAVVSEKGPIPMHLLGNMWAQTWDNIADFTTPFPDSQQLDVTEEMARQGYNPIQMFEMGDEFFVSLNMTKLPASFWEKSILEKPDDGRELVCHASAWDFYRTDDVRIKQCTRVNMEDFFTVHHELGHIQYFLQYQHQPSMYREGANPGFHEAVGDVLSLSVSTPKHLEKIGLLKDFTLDEESKVNQFYRSGLGKLVFLPFAYTIDKYRWGIFRGDIQPHEYNCKFWEMRSKFSGIEPPVVRTEKDFDAPAKYHVSADVEYLRYLVSYIIQFQFHRSACELAGEYVKGDPEKTVNNCDIYQSVNAGNAIKEMLAMGSSKPWPDAMEALTGQRKMSADALLEYFQPLYDWLVVENKRLGAYVGWEATEKCQPE |
| Aa-8366 | MRAPRSPPIPAGEKVESDAAENEKDASTEKESNKDGAGDASATASVSSPTTEKQPAESDSDAANKTQENGGSADETLNDSKTAAAEDSNXXXXXXXXXXXXXXXXXXXXXXXXXXXXXXXXXXXXNGECEKVPEEPTEDAATAAAAAAPATSDVTETKPEAAEVAPATEAVKAVEEPVVEAAVASPAQETAPATAETTPATETAPAVEPAAEKPVEEEKKVEEEKPAAAPVAVPEQQAEPAKEVEKVVEPVAVVEVNKAKQVEQTPPAEVVAAAVETETKSEPIVVEQTEKPVTEVVSDKPAAEAAPAATETVAVEPVPAEPVVAADSNGDSTPPPPLPSIPPPSQVMVFAEASMSQGTSEPRTVVPSVRTLLTGTPVTVPVPFPFSVPGSGLKENDGNGNETKTKGKDDG |
| Aa-8692 | MELLKALVFLLSALFINTVYPLQRPATFCQCPCEARVAINPAWFSCVAISSLKTGGYLTADEQSAYIPNAERYTFVTNNNQFYVTGKWTVAAYNAANSTYSLMNHHVKEWLHAGSDEWARDASRRYLLTKIKGNSNMAPRDGYWQFIPDAKMGNAVYRIRNALTGEFLYVDDEQHEQRKQFKRVYLWRHAGHYQATDVRHWFRLAKC |
| Aa-8859 | MNPTEWFSEISEQLWPNQCFSLKVKKVLHEERSKFQDIKLLETYSHGTVLVLDGIIQCTERDEFAYQEMISFLPLCCHPNPKKVLIVGGGDGGVAREVVKHPLVEEVHQVEIDDRVVELSKKYLPFMACGFDSPKLKLTIGDGFEYMKQREGEFDVIITDSSDPIGPAESLFQESYFALAKKALRPNGIICSQGGTFWIDMDHIKSTLDHCRKHFPVVGYGVASVPSYPTGQIGFFIASSNENTKLNEPAKTFTDEEIDNMNMRYYTSDVHRAAFTLPRYAAKKLY |
| Aa-9223 | FATRGQDKIERKRSKKINEGKGSGASSAPAAEKPAPEFIDHRIKIYDELKAQYLEELARSRRPDQVTLRMGRXXXXXWESTPYDVAKGISQGLADNTVIARVNNELWDLDRPLEGDCKLQLLKFDDPDAQAVFWHSSAHILGEAMEKRYGGHLCYGPPIENGFYYDMFLEGSGISNQDYGVLESEVKKIVKDKQPFERLEMKKSDLLKMFEYNEFKCRILNEKVTTDTTTVYRCGPLIDLCRGPHVRHTGKVKALKVVKNSSTYWEGKADAETLQRVYGISFPDPKQLKEWEKIQEEAAKRDHRKLGKEQELFFFHELSPGSCFFQPKGAHIYNTLMNFIRSEYRKRGFQEVISPNIYNAKLWQTSGHWQHYAENMFSFESEKETFALKPMNCPGHCLIFDHRNRSWRELPLRMADFGVLHRNELSGALTGLTRVRRFQQDDAHIFCMPEQIKQEITGCLDFLAHVYGVFGFTFNLVLSTRPEKYLGDIEVWNEAEKALAESLDKFGQPWKENPGDGAFYGPKIDITIMDALKRNHQCATIQLDFQLPIRFNLNYIDDNGEKKRPVIIHRAVLGSVERMIAILTESYAGKWPFWLSPRQVMVVPVGPAYDEYADKVRQRLHEAGFMAEADLDAGDTMNKKIRNAQLAQFNFILVVGEKERSSETVNVRTRDNKVHGEVSVADLVAKLRRLADEFARGEDQF |
| Aa-9320 | MSLGHRGRWQPAVTNSSSIEPAMLLHSYTVVAICLCALATSVATQAENATAAAEAPTVREEEESLMKAIGLVVDKLKDFHSHLLTDLRSIGQQPEGNTDAEATPDGTTPKPEEKNLWKKLKQKVSEQWNALKNWWGDK |
| Aa-9784 | MAEMEDTHFEAGDSGASATFPMQCSALRKNGHVMLKARPCKIVEMSTSKTGKHGHAKVHLVGIDIFTGKKYEDICPSTHNMDVPHVKREDYQLTDIDDGFLVLLNDNGDLREDLKIPDGDLGTQLRSEFDSGKELVCTVLKSCGEETVIAIKNNTAADKN |
| Aa-9828 | MGKAQSKRSVDITTDPAKDSVVTEGTGKLEKIEDVDQLKSQANGDAQHNEGESEKKVESDAAENEKDASTEKESNKDGAGDASATASVSSPTTEKQPAESDSDAANKTQENGGSADETLNDSKTAAAEDSNKKPKKVKKKWSFRSISFSKKDKQKPAKKEKEGEEKVNGECEKVPEEPTEDAATAAAAAAPATSDVTETKPEAAEVAPATEAVKAVEEPVVEAAVASPAQETAPATAETTPATETAPAVEPAAEKPVEEEKKVEEEKPAAAPVAVPEQQAEPAKEAEKVVEPVAVVEVNKAKQVEQTPPAEVVAAAVETETKSEPIVVEQTEKPVTEVVSDKPAAEAAPAATETVAVEPVPAEPVVAADSNGDSTPPPPLPSIPPPSQVMVFAEASMSQGTSEPEPDSLNSIPEPSSSSLPVEKQEQKQEEQVVVVEESKQAEEQAPVEEPVVEKKVEVKPVESAPAPVEVVTPAPVEEAKKEETPAPVVEEKIVEKVVEAVQADAPAVEAVVELKETVSEVVEEILEKAVDKVEEQVAAPVVAEETKPVDQAPVETEAPKEVAPEVVAEPAKAEETPAPVEVVPAPVEEKLVEATPAPVEEKPVEPTPAPVEETPAPVEEKPAPVEEKKVEEQPAKAEPAPVEEAPVAAVEQKPVEQAPAPVEEKPVEPAPVEEKPVEKVTAPVEEAKPVEAAPAAEEPKSEELIPAPEPVSEADEISNTIDDLPPPPPPPATDDEPVPDSLPSPLPTIAAAAPTTGATDEQNLSVDISSPLPQNSLESLPSPPTLSQSDAMSLPPPPESPSATLTSIEATEEQSLPAAPVSEPTEALPTPPESAPVESSEPQKPAEAQVPQVVAEEKPVEVPAAAAAATTESKVEKEPVAAEEPAATTAAATEPAPESSKKEEQAVEEKEKSEAPAPQPTEATEIVVKKQQQQNGTVENGTAASTENGTTENGAATVENGQNGVHETSTTESMNGDTEHKKEEKIPEKAAQLQQPPPAAEVTAE |
