# Supplementary material for: Identification and Characterization of Seminal Fluid Proteins in the Asian Tiger Mosquito, Aedes albopictus
Source: PLoS Negl Trop Dis. 2014 Jun 19;8(6):e2946. doi: 10.1371/journal.pntd.0002946 (PMC4063707; doi:10.1371/journal.pntd.0002946)
Supplement: Table S4 — Amino acid sequences of Ae. albopictus putative sperm proteins. (DOCX) [file pntd.0002946.s004.docx]

| **Putative sperm protein** | **Amino acid sequence** |
| --- | --- |
| Aa-10138 | EATNVRANLIFQVAERLLQVFVQLVEIGLDLRFTVLHWVLELHRRQLVQDVAHRVTDHVPGDLVLRLGGGFHGVASHVVETDHVPQHTDRLVERTEPIVRRVRVLLQEIVLQELGHLQGDLVGFGQRRLTDQLHDFGQIFFLLQDLLDLGTQRDELLEVLIVEVVQGAHVFTVGDQPVDRGEVLTLSQLLVQTPEHLYDTEGGGCDGIGEVATRWRYGTDDGHRTFTFRITETLDATGTLVEGGQTSTQVGRIPGIGRHFSQTTGNFSKSFGPTRGRVSHHRYVVTHITEVLRQGNTGVDGSFTSSDRHVGGVGDQCGTLHDGLGDTVNFNRQLREITQYFRHLVTTFSATDVDNDIGVGVLGQGLRDDSLTATESSGDGSGTTLDAREQRIQHTLTGQQWVIGGQLLRHWTWLRYIAPPGNYTVDDIILETEFDGEINKWSMLQVWPVRQPRPVTEKLPANHPLLTGQRVLDSLFPCVQGGTTAIPGAFGCGKTVISQALSKYSNSDVIIYVGCGERGNEMSEVLRDFPELSVEIDGVTESIMKRTALVANTSNMPVAAREASIYTGITLSEYFRDMGYNVSMMADSTSRWAEALREISGRLAEMPADSGYPAYLGARLASFYERAGRVKCLGNPEREGSVSIVGAVSPPGGDFSDPVTSATLGIVQVFWGLDKKLAQRKHFPSINWLISYSKYMRALDDFYDKNFQEFVPLRTKVKEILQEEEDLSEIVQLVGKASLAETDKITLEVAKLLKDDFLQQNSYSAYDRFCPFYKTVGMLRNMIGFYDMARHAVETTAQSENKITWNVIRDSMGNILYQLSSMKFKDPVKDGEAKIKADFDQLYEDLQQAFRNLED |
| Aa-10439 | MAGLLSISSRNLPRTALRISGGLRSLEGSSQQVNNGLRSYHQGRAALQRLVRATVAESAPIQSSTAASNAASRWLQTERSIFTSARLLNSEVVKVPPFADSVSEGDVKFEKKVGDAVAADEVVMEIETDKTTVGVPAPAHGIIEEIYVADGDTVKAGQQLFKLKITGEAPAAGAPKAEAPAPAAAAPPPPPP |
| Aa-11143 | MHRARTALQLMGHPAGQQSFGSWLIRNPSSKLTGELVAASSVKLYNSAAAEPFLNGSSSNYIDDMYNAWLRDPASVHASWDAYFRNNSYEAPPSLAPIPRNHVPASQYLGSAVPALASGSSAVGTRIDDKLIDDHLAVQAIIRSYQSRGHLVADLDPLGILNADVQRDLEHNLRANEKVTRSYMNFEEADMDRVFKLPSTTFIGGKEKFLPLREILSRLERAYCNKIGVEFMFINSLEQCNWIRERFETPNIMNYTSEEKRLLLARLTRATGFEAFLAKKFSSEKRFGLEGCEIMIPAMKEVIDVSTRLGVESIIMGMPHRGRLNVLANVCRKPLNQIFTQFAGLEAADDGSGDVKYHLGTYIERLNRVTNKNIRLAVVANPSHLEAVDPVVQGKTRAEQFYRGDGEGKKVMSILLHGDAAFSGQGVVYETMHLSDLPDYTTHGTVHIVVNNQIGFTTDPRHSRSSPYCTDVARVVNAPIFHVNSDDPEAVMHVCRVAAEWRATFHKDVIIDLVSYRRNGHNEIDEPMFTQPLMYKKIRGIKPVLDIYANQLIAEGCVTADEVKSVKDKYEKICDEAMEQAKVETHIKYKDWLDSPWSGFFEGKDPLKVAPTGVIEETLVHIGNRFSSPPPNAAEFAIHKGLMRVLAARKEMVDNKSVDWALGEAMAFGSLLKEGIHVRLSGQDVERGTFSHRHHVLHHQTVDKATYRPLCHLYPDQAPYTVCNSSLSEFGVLGFELGYSMTNPNALVIWEAQFGDFNNTAQCIIDQFISSGQSKWVRQSGLVMLLPHGMEGMGPEHSSARAERFLQMCSDDPDYFPPESEEFAIRQLHDINWIVANCSTPANYFHIMRRQIALPFRKPLVLLTPKSLLRHPEARSSFSEMTDGTEFQRIIPDASAASENPSSVKKLIFCSGRVYYDLVKARRERKLDSDIAISRLEQISPFPYDLIKAECAKYPNAELVWAQEEHKNQGYWTYIEPRFDTAINSTRDLSVQDKLVLQKTAQGFNISEGTFNAPTDGTRGRKVKISSKPLSYVGRPCSASTATGSKAQHTKELKNLLDNAMAL |
| Aa-11291 | MVYIVKDGADFDSKLESAGDKLVVVDFFATWCGPCKVIAPKLEELQNKYAEKVLFVKVDVDECEDLAAKYEISSMPTFLYIKNKKVVDQFSGANAEKLEHYIKRFTE |
| Aa-11848 | MYRLLVQKTFLSGSKRQSTQLLRSLSTRGAANIPQFREAGFGKVLVVLSPFLVGGGVVAYAKYDNEFRKTLVTNVPALEPVLKTLLQEENPLDEVSKKLDEISSTISGYTSTVTGFFTGGTKEEEKKAEKKVDLPPVTRSKSVHVPVPAPRRIQPKTRNRKGGRYVQRLRTESTSLNLNFVRPYLPYVLRRPYRRPKNP |
| Aa-12362 | MKLLVPLALVAALAVLSCTAEEKKEQDYGTVVGIDLGTTYSCVGVYKNGRVEIIANDQGNRITPSYVAFTADGERLIGDAAKNQLTTNPENTVFDAKRLIGREFTDSTVQHDAKLLPFKVIEKNSKPHIKVSTSQGDKVFAPEEISAMVLGKMKETAEAYLGKKVTHAVVTVPAYFNDAQRQATKDAGVIAGLNVMRIINEPTAAAIAYGLDKKDGEKNVLVFDLGGGTFDVSLLTIDNGVFEVVATNGDTHLGGEDFDQRVMDHFIKLYKKKKGKDIRKDNRAVQKLRREVEKAKRALSSSHQVRIEIESFYEGDDFSETLTRAKFEELNMDLFRSTMKPVQKVLEDADMNKKDVDEIVLVGGSTRIPKVQQLVKEFFNGKEPSRGINPDEAVAYGAAVQAGVLSGEQDTEAIVLLDVNPLTMGIETVGGVMTKLIPRNTVIPTKKSQIFSTASDNQHTVTIQVYEGERPMTKDNHLLGKFDLTGIPPAPRGIPQIEVSFEIDANGILQVSAEDKGTGNREKIVITNDQNRLTPEDIERMIKDAERFADDDKKLKERVEARNELESYAYSLKNQLGDKDKLGAKVADDDKAKMEEAIDEKIKWLDENQDADSEDYKKQKKELEDVVQPIIAKLYASTGGSPPPTAGEDDDLKDEL |
| Aa-13766 | MAAFVGRICGSGLLNSKNGVTLQKVQQFSTSRPWTRSQKLATAAGIAVGGAGALLYALEQSVSASGTEVHPPELPWSHKGVFNSLDHASVRRGYEVYKQVCAACHSMRFIAYRNLVGVSHTEAEAKAEAAEIMVKDGPDEAGNYFMRPGKLSDYFPSPYPNEEAARAANNGAYPPDLSYIALARHGGEDYLFALLTGYCDAPAGIVLRDGQYYNPYFPGGAISMAQALYNESAEYTDGTPASAAQLAKDVSTFLVWSADPCHDERKRMGIKSLGIILVLGSLAYYLKRHKWAALKTRKIAFYPKEK |
| Aa-140678 | MCDPCCSPCDPCYPCYPCGPCPPPCAPGSLCDPCVPKTYAAHDLNCMPQCPVICPPAQCGPRFITVQQPPRVVAQRKLINCARTVVDKHVVPRTKTIVEPKIIYEPRCYVEPCIIYRKRVVPDPKILYYKRIVPDPKIVCTPRTIVEPKEICTTMVCQPKPQTVQIPPPPQYCCVPTGTGFTKAPACSPCDPVCQPRRC |
| Aa-14332 | MSMISARLAAQVARQLPSTASQVAKIAVPAVTVAARNLHVSTANRGAEISSILEESILGSAPKADLEETGRVLSIGDGIARVYGLKNIQADEMVEFSSGLKGMALNLEPDNVGVVVFGNDKLIKEGDIVKRTGAIVDVPVGDEILGRVVDALGNAIDGKGEIKTNQRFRVGIKAPGIIPRVSVREPMQTGIKAVDSLVPIGRGQRELIIGDSQTGKTALAIDTIINQQRFNNGTDESKKLYCIYVAIGQKRSTVAQIVKRLTDAGAMNYTIIVSATASDAAPLQYLAPYSGCAMGEYFRDNGKHALIIYDDLSKQAVAYRQMSLLLRRPPGREAYPGDVFYLHSRLLERAAKMNPTLGGGSLTALPVIETQAGDVSVSDRND |
| Aa-145956 | MCDPCCGPVCGPCGPCGPCGPCGPCGPCGPCGPCPPCGPCDPCCEPRSFSAGQLACMPQCPTGCPPPTCGPRFITVQQPPRLVSQKKVINCTRTVIDKHVLPQTKAIVEPKLIYQPKTIVEPCVIFKKRIVPEPKVIYYSRTVPDPKVVCTQRLVVEPKEYCTTMVCQPKPQIVPVPPAKEYCCMSTGTTFFNNACCPPTPGPICPPRKC |
| Aa-14696 | YSCVGFVRRCFSLERLVVTSKLAIRKKIIHIPEVTETSRNLVPDTMAEGKNEDLATAILKRKERPNRLIVDEAGNDDNSVISLSQAKMDELQLFRGDTVLLKGKRRKETVCIVLSDDNCPDEKIRMNRVVRNNLRVRLGDVVSIQSCPDVKYGKRVHILPIDDTVEGLTGNLFDVYLRPYFLEAYRPIHSDDTFIVRGGMRAVEFKVVGADPSPYCIVAPETVIHCEGDPIKREEEEEALNAVGYDDIGGCRKQLAQIKEMVELPLRHPSLFKAIGVKPPRGILMYGPPGTGKTLIARAVANETGAFFFLINGPEIMSKLAGESESNLRKAFEEAEKNSPAIIFIDELDAIAPKREKTHGEVERRIVSQLLTLMDGMKKSSHVIVMAATNRPNSIDPALRRSEIMSKLAGESESNLRKAFEEAEK |
| Aa-14916 | MGVPAGDVEKGKKLFVQRCSQCHTVEAGGKHKVGPNLHGIFGRKTGQAAGFSYTDANKAKGITWSEDTLFEYLENPKKYIPGTKMIFAGLKKPNERADLIAYLKSSTA |
| Aa-150405 | NFPQIVNPVSRNRKLSQNGCSRPAIDACPAKCIRIPAAAEVFHEYWRKPXXKGLVLGLYEQEIETDEPRLTPVAGHFDAKTEGQLVNLIKESNLKGKVGQVKVFNNIDPDYGSVAVVGLGLEGLGYNELEQLDEGLENVRIGAGVGAKCLAKQGCSRISVDPMQAAEQAAEGSGLATWKYQANRMKSERIPTPKLELFDSPDGDAWTRGLFKADAQNLARSLSDAPGNQITPTAFAQAAVDALCPCGVSTEVRNMDWIESKSLGSFLAVAKSSCEPPIFLEISYCGEHDSGRPIMLVGKGITFNSGGLCLKEPHGMSQYRASMSGAASIVATIRAAAALSLPVNLVGLIPLCENMPSGMAFKPGDVITTLNGKTVAIHDTNNAGRLIMADTFIYGQNTFKPKVVMDVATLTNGVTHALGGAASGVFSNSDFLWKQMQKAGAITGDRVWRMPLWKYYTHKVTNYTNVDISNTGXXXXGSSCLGAAFLKEFVPCVDWIHLDITGVGMLKKGVGIPYLAEERMTGRPTRTLVQFLYQMACPDEQVKSLSKESCA |
| Aa-15044 | MLSSLKNVLSTGSRIQAELVRNYAAKSAAKAAAGAQGKVVAVIGAVVDVQFDDNLPPILNALEVQERPSRLVLEVAQHLGENTVRTIAMDGTEGLVRGQRVLDTGSPIRIPVGAETLGRIINVIGEPIDERGPIETNLSAPIHAEAPEFIEMSVEQEILVTGIKVVDLLAPYAKGGKIGLFGGAGVGKTVLIMELINNVAKAHGGYSVFAGVGERTREGNDLYNEMIEGGVISLKDKTSKVALVYGQMNEPPGARARVALTGLTVAEYFRDQEGQDVLLFIDNIFRFTQAGSEVSALLGRIPSAVGYQPTLATDMGSMQERITTTTKGSITSVQAIYVPADDLTDPAPATTFAHLDATTVLSRAIAELGIYPAVDPLDSTSRIMDPNIIGAEHYNIARGVQKILQDYKSLQDIIAILGMDELSEEDKLTVARARKIQRFLSQPFQVAEVFTGHAGKLVPLEETIKGFTKILNGELDHLPEVAFYMVGPIEEVVEKAERLAKEAA |
| Aa-150492 | MIVWRSLLKPVATKPFGGLLHRNYAQAVLDKCASPPSKGLILGIYADEEDAFDTGTLTPAGARYNEAQTNGRLLELVRLAGPIPKRGEVRVFYDLEPTFKAVAVCGLGSDCLGYNSAEKLDESKEAIRNAVARGCRELQKLETNFIYVEDMGHAESAAEGAHMSVWINQELKRPNKRKFVPHCQLHVDRALPCDADGWRIGIVKAEAQNLARQLQEMPSNLMTPTTFAQNVVQILANSGVNVEVKVKSWAEAQGMTSFLAVSKGSCQPPIFLELSYYGAGGKEKPIVLVGQGNTFDSGGICAKPCHGLQNMRGDMSGAACVVATCRAIASLKLPVNIRGLIPLCEHMIGCNAIKPGDVVPVKNGKSIEVVDADREGPMVLVDALLYAEIFGPKYIVDVATSSEHVVDSFGRVCSAVYTNSEQLWQRIKNAGVHTGDRLWRLPLWDYFTQQVCSAEHVDVQNVGRGVGGESCCQAAFCESXPCGQWMHIDAHNVM |
| Aa-150496 | VVPWTSEHARGYVRGCVRSGHVSCHRLAQVAGKHPRIDSALRAHDWLQRHQAWRRCAGQEWKEYPLCALGDADREGPMVLVDALLYAEIFGPKYIVDVATSSEHVVDSFGRVCSAVYTNSEQLWQRIKNAGVHTGDRLWRLPLWDYFTQQVCSAEHVDVQNVGRGVGGESCCQAAFLREFLPCGQWMHIDAHNVMTTKGVDYPYLRAGMAGRPTRTLIEFIAQGVCKQTDMCVQKTADKK |
| Aa-15467 | MSKIGINGFGRIGRLVLRAAVDKGAQVVAVNDPFIGVDYMVYLFKYDSTHGRFKGEVSVQDGCLVVNGMKIQVFQERDPKAIPWSKAGAEYVVESTGVFTTIDKASAHLEGGAKKVIISAPSADAPMFVVGVNLDAYNPSMKVVSNASCTTNCLAPLAKVINDNFGILEGLMTTVHATTATQKTVDGPSGKLWRDGRGAGQNIIPAATGAAKAVGKVIPALNGKLTGMAFRVPTPNVSVVDLTCRLSKPASYDQIKQKVKEAAEGPLKGILDYTEEEVVSTDFVGDTHSSIFDAKAGIQLSDTFVKLISWYDNEFGYSNRVVDLIKYMQSKD |
| Aa-15912 | MSVNRTISAHQAAKEHVLAVSRDFISQPRLTYKTVSGVNGPLVILDEVKFPKFAEIVQLRLNDGTIRSGQVLEVSGSKAVVQVFEGTSGIDAKNTVCEFTGDILRTPVSEDMLGRVFNGSGKPIDKGPPILAEDFLDIQGQPINPWSRIYPEEMIQTGISAIDVMNSIARGQKIPIFSAAGLPHNEIAAQICRQAGLVKHTGKSVLDEHEDNFAIVFAAMGVNMETARFFKQDFEENGSMENVCLFLNLANDPTIERIITPRLALTAAEFLAYQCEKHVLVILTDMSSYAEALREVSAAREEVPGRRGFPGYMYTDLATIYERAGRVEGRNGSITQIPILTMPNDDITHPIPDLTGYITEGQIYVDRQLHNRQIYPPVNVLPSLSRLMKSAIGEGMTRKDHSDVSNQLYACYAIGKDVQAMKAVVGEEALTPDDLLYLEFLTKFEKNFISQGNYENRTVFESLDIGWQLLRIFPKEMLKRIPASILAEFYPRDSRH |
| Aa-159788 | MREIVHIQAGQCGNQIGAKFWEVISDEHGIDATGAYCGDSDLQLERINVYYNEATGGKYVPRAVLVDLEPGTMDSVRAGPFGQLFRPDNFVFGQSGAGNNWAKGHYTEGAELVDSVLDVVRKESEGCDCLQGFQLTHSLGGGTGSGMGTLLISKIREEYPDRIMNTFSVVPSPKVSDTVVEPYNATLSVHQLVENTDESYCIDNEALYDICFRTLKLTTPTYGDLNHLVSATMSGVTTCLRFPGQLNADLRKLAVNMVPFPRLHFFMTGFAPLTSRGSQQYRALTVPELTQQMFDAKNMMAACDPRHGRYLTVAAIFRGRMSMKEVDEQMLNIQSKNSSYFVEWIPNNVKTAVCDIPPRGLKMSSTFIGNSTAIQEIFKRIAEQFTAMFRRKAFLHWYTGEGMDEMEFTEAESNMNDLVSEYQQYQEATADEEGEFDEEEEGGEE |
| Aa-16730 | MELISSGKKFLVSPLYSTWIFGLPSTEIIANDQGNRTTPSYVAFTDTERLIGDAAKNQVAMNPTNTIFDAKRLIGRKFDDPAIQADMKHWPFDVISVEGKPKIQVEYKGETKNFFPEEISSMVLTKMKETAEAYLGKTVSNAVVTVPAYFNDSQRQATKDAGTISGLNVLRIINEPTAAAIAYGLDKKTAGERNVLIFDLGGGTFDVSILSIDDGIFEVKSTAGDTHLGGEDFDNRLVNHFAQEFKRKHKKDLSTNKRALRRLRTACERAKRTLSSSTQASIEIDSLFEGTDFYTSITRARFEELNADLFRSTMEPVEKAIRDAKMDKASIHDIVLVGGSTRIPKVQKLLQDFFNGKELNKSINPDEAVAYGAAVQAAILHGDKSEEVQDLLLLDVTPLSLGIETAGGVMSVLIKRNTTIPTKQTQTFTTYSDNQPGVLIQVFEGERAMTKDNNLLGKFELSGIPPAPRGVPQIEVTFDIDANGILNVTALEKSTNKENKITITNDKGRLSKEDIERMVNEAEKYRSEDEKQKETISAKNALESYCFNMKATMEDDKLKDKITDSDKTLIMDKCNDTIKWLDANQLAEKEEYEHRQKELESVCNPIITKLYQGAGGAPGGMPGFPGGAPGAGAGAAPGAGSGSGPTIEEVD |
| Aa-167868 | MTSLEQKREAFRKYLESAGAIDCLSKALIRLYQEQEKPDDACKFIRQTMCETCPTDEEVANMIVELADARQEICCLKREIVSYKGELRRSASEVALALEEGFKKLQEDEECTSLLKKHLTQEVFDELKEKKTALKSTLLDCIQSGLENHDSGVGVYASDAECYELFAPLFNPIIDEYHGINLAEAPHPASDWGDASTFENLDPENEFIISTRVRCGRSIEGFPFNPRLKMAMYEEIMDRIKTVLTGLEEDDLKGEFHPLETMSDELKQQLIDDHYLFKEGDRFLQAAEACRFWPIGRAIYYNEAKSFVVWVNEEDHLRIISMEKGGDLGAIYQRLVRAVEAIGKDVAFSRNDQFGFLTFCPSNLGTTIRASVHIKLPNLGSNRAKLEEEAGKFNLQVRGTRGEHTDSEGGVFDISNKRRLGLTEFDAVSEMYNGIKQLIDLEKSTEPGEAPPAEDAAPAEGEDEEPTAE |
| Aa-167882 | THCQAVKMGIKRIRLKILLFLVPFVVAIVSGTTHHRPRHARPIPEVIISKAADLVQRDDIGSECSKSYCPENNSRMKRQDTDDAKPAPADDTAGAANGTSGSAAAVAAATSDKDAGNAPLPPPVQVFPNMPKDFPFPSLDVVQHPPVPTRPRRPIRVQSPYPAGNIPSASADSSSTLEFSNQDPISPKDLTPVEPPVEESVTASDSVSLPELGETPEVLDLSEFNVPGSNDPVVLPPAEKVGASSGDVPTVAKTQTPHVEAPQITNPLPSAPEVTNMAPPRQHTRVMGPQDWRFSEQPQRRCQSKQTCRLGNAPPMGGPQMAGPPMGGPPMAGPPMGQPAMAQPPMAQPPTGCRALPGQTMAPAAPTAAIGEDPWKVAEQQAFVEIKNRDDPPATEDKPADSAGERSGLAFAALMLANGLYLLL |
| Aa-16902 | MFKSVVPVLAQVPAEVCLGQQNRGMATLKAISIRLKSVKNIQKITQSMKMVSAAKYARAERDLKQARPYGVGAQQFYEKAEVAAKEEEPKKLYIAVTSDRGLCGAVHTGVARHIRGDLAADPNIKVICVGDKSRAILQRLYSKNIEMVCNEVGRLPPTFLDAAKLTNAILNLGYEYTDGKIIYNKFKSVVSYAVADMPIFSLKSVESAEKLPVYDSLDSDVIQNYLEFSMASLLFFAMKEGACSEQSSRMTAMDNASKNAGEMIDKLTLTFNRTRQAVITRELIEIISGASALESKD |
| Aa-17325 | MADLGAPVRVSPLIKFGRWSFLAIGVAYGAYHQQRLAKREVGIREVEAQQKVIRDAKLAEEKKRQQAEEAKAIAELSNPAKK |
| Aa-174817 | HTLPLAMAGTMDELLKELNYDFNYLLHEVRAASRNLNLESRRTVEAWIQKLSATNQSMEEVRLRNDFLFYLARSCEEGTLMPPFDQRPPSGYVLNSSHLMPMLGTEMTASTSTRPIYEAYSGPAASAGQSQKAELFKRSPDGGAFLVSQPVPRCGAFCYLAVVSKQPK |
| Aa-175987 | MYAGHFFKQAGCKVIGIKEADVALLNEEGIDVGELASYKRLNNTIKGFKGAKETKEDLLLHPCDILIPAAVEKSINSENAAKIQAKIIAEGANGPTTPAADAVLQSRKILVIPDLYCNAGGVTASYFEYLKNINHISFGKLSFRQESQNLREVLRSVEESLKEAGVLRQAQANRRTQTLSGQRQRSGCRGLGA |
| Aa-176049 | MDRDMACPPCPPSRERSSTSGAASVHAHGKVTTNPFFNFLREFRGKNGSLTVVEAAVQGANVWNRMSAEERSPYVKLACGHPQRVTPCGIPSSKGSNGHRSRSRARSRSSRRGPTDRSASRAGRKRRRSQSSGGRSHSRGRRARRC |
| Aa-176077 | MKLFRKCSRANRCQPFLVLVLAVCASRISPAWTQSWGSPQQQPGGGQYPPQQQPPQIPLQQPGMPPQQQPGGQYNPPQQQPGGQYPPSQQPGGQYLPPQQPGGQYPPPQQPGIPPQGSNPQDGYYIIPQPQGSPQVYPTMGPPTMSPTPTPPTGTGGKNFDDFQTECTAQSRLIGSINAQWNDAPYMA |
| Aa-176110 | MASLRDLKSDRHHNYGLGHFCGGIIIHPYMVLTAASCIVNRLESDIGVVVGTLNRRKQATWSQLLFVQKMVSHPSYTFQAGGHDIGLLLLRTAVFLGSKVAIAPLAYKDPRSETDCTLYGWGETTAQGQLISDCLRKATVKVQNLEECKQRFLAINIKLPPSVFCAGHFGGGPDACQGDIGGPAVCEGNVHGVIGNKVGCGAQSAGKIYTNVYSFRTWIDYTMNSLKQEFKNLYPEGALNSMRSGAVLRDDAPAAAETPATAGVAAKCASVGLALATGLTLLLA |
| Aa-18112 | MLRAPLTRVLVIGSRCPVTQTAVRTTATTPAKAPEKIEVFVDDVPVMVEPGTTVLQAAAAVGVEIPRFCYHERLAVAGNCRMCLVEVXXXXXXXXXXXXXVMKGWRIKTNSEMTRKAREGVMEFLLMNHPLDCPICDQGGECDLQDQAMAFGSDRSRFTDIDHSGKRAVEDKDIGPLVKTIMTRCIHCTRCIRFASEVAGVDDLGTTGRGNDMQIGTYVEKFFLSELSGNVIDLCPVGALTNKPYSFVARPWEIRKVESIDVLDAVGSNIIVSTRTGEVLRILPRENEEINEEWLSDKSRFACDGLKRQRLIAPMLRNPSGELEAVEWESALITIAQALRGAPKGKIAAVAGGLADAEALIALKDLLNRLGSETLCSEQKFPTDGSGTDFRSSYLLNSSIAACEEADLVLLVGTNPRYEAPLLNTRLRKGYVHNEQNIAMIGPKVNLSYEYEHLGNDPSLVRDIASGNHPFAKKLKAAKKPLIIVGANQLARKDGSAFVTALHVFANSLQPADPNWKVWNVLQTTAAQTAALDVGYSAGVDSVLASDPKVLFLLGADAGSIKKEQLPKDCFVIYQGHHGDAGAEMAHAILPGAAYTEKQGTYVNTEGRAQQTLVAVTPPGLAREDWKILRALSEIAGAPLPYDTLDELRSRMEDIAPHLVRYGRLEKANFFKAAGELLRNSAVHFDGSKVDVDQKQLADFFMTDPITRASPTMAKCVTAAKKQSAKATN |
| Aa-182963 | METPNLEENFKLFYRIAKVLILELNNEDRVLAAQWLRKLAACQSPEDSERRNHFMTLLLITLQQKRIVGPFKNSPVDGNKLEPFQKEFQTADIQKLLDEELAQRPPPPLAQFGLTGGKISTEFAVTQEIPKFGVHFYYVYTLTPIYDFQRANQSRIPPPMQAPSQSTMRNLNAGVENLLRTEKKKLKVTVDQLGRKQIQIRDEHFAEFSGSGRPRRVPKRLQQRLGIRAPEPEPEPEIQLTSFVQEPSPPRRTTPQPATSTTVEQAVAAAVGAAVAAAASTTPPRTGGIPRPTGAIPKQPRLSGGVPRYTGAIPKATRVQQPPPQPSPQHPPAIEVQPPSPKTPVRMPARGPAVDPNMDMVPMPDYMPDPFATPTPPLFAKRGRTQGLTYKPEDFELDPQTKFDYPRQRIAKSKWTPSKRGERVMIRDIIKQHPPSEQTLQLSPDVREIAPLSEIDAQCTEIARRRKLVFQTDDDRMRNVFQKFYASRPVSPKALQFTIEEEASTIQPFLLDTTPVGEQRIQSPASLPRIVTPRSVTPRMPTPRPATPGSQSSRPTSPRSPTPRRTTPREASPRPLTPRDATPRAAPPARAPRQISDWFVEDEGLLDEEDIQLRDELIYIPSPIRAQTPRAYSPSPRQVDEGLLEDEEIEFVDDMPSPSQYRSPVRPPVHSPIQYTTPYYSPEVSPGGTTGRLIDAGDEVPAISPKSPHSIGSMMDDIMLTTPERMERLGTIMSQIGQQVEAHRELIQNIEELPTSPSLENLRVECGRGASALSAMHSRLRAVRESAQAQQPPATGVGELEVVPEEPEPFSPLRLSLQFQDLADVINTTMEGAQIIQQVAESVPEPVVQDAAERSLDTVDALQQISDRVHDVADAWEKRITDLDTSSLGLIDDDVMDESLFSSPARETANRMVEALDQSVREVIEEGGDPRSPAARNLQGTLRDFREALQNLSLESPSFLDESFPEALLDDTIDDLEASIQTQAAAISAVSKTPEKAAILDRAEEIVESMNDVLDEVQTIVTPEAGEQDDDDKFVAEFFAESSPQQELPPASEAMQQEMLNLLEKTRQQQQALLESSLEIEENAVDAAAVAASQQATQRIQQAQQAVTDMIDTVARSPVVYDGSFAWLGPMSIEPEERPTAAGRRQVRSRAAPRPPAGRMPAARSAASRPAAPRPAASRTAVARPSATRPPAARPPAARPPAARPAATRPSATRVQTRPQAIRRPPQARGASPEPLALPAGVQAPRPRAGSATPIPVRRPPRPIPLPGAASVTEITTQSSSSTIRTMIPRPGSATSER |
| Aa-182964 | HDVAEPVVQDAAERSLDTVDALQQISDRVHDVADAWEKRITDLDTSSLGLIDDDVMDESLFSSPARETANRMVEALDQSVREVIEEGGDPRSPAARNLQGTLRDFREALQNLSLESPSFLDESFPEALLDDTIDDLEASIQTQAAAISAVSKTPEKAAILDRAEEIVESMNDVLDEVQTIVTPEAGEQDDDDKFVEEFFAESSPQPELPPASEAMQQEMLNLLEKTRQQQQALLESSLEIEENAVDAAAVAASQQATQRIQQAQQAVTDMIETVARSPVVYDGSFAWLGPMSIEPEERPTAAGRRQVRPRAAPRPPAGRMPAARSAAPRPAAPRPAASRTAVARPSAARPPAARPPAARPPAARPSATRVQARPQAIRRPPQARGASPEPLALPAGVQAPRPRAGSAMPIPVRRPPRPIPLPGAASVTEITTQSSSSTIRTMIPRPGSATSER |
| Aa-183071 | MSLLLKSSVNLYKLNPKSIQQQAARSFLTKKKCPPKTEEESQCGRAASASGRVNEIAAGYKLLKDKQARFQVKDNLPVWLKGGPMDRMLYVSTVGLSVVGLGCSLAFISMMALK |
| Aa-183082 | MYQMSRIGPGPAGYGLPPTVGYPEHDVRKDRKPMFSMRSRPITRYDTLGPGPARYGLGKMTRVGRPNNPSYSLAKRFSLFKPDNTPGPGAHNNDKVPSMKGNRSPAYSLGMRLKETSRDMIPGPDRYAYDLNIYKNRNPIYSMRERTTQPYALEGPGPNKYGDLDRNVTHKRNPRYTMRQTYPIMGDKCPKPGPNQYGLMELKPGTTAPKYSFGTRHSMWRPPMVIPGDNC |
| Aa-183621 | MRGWGFREALKYPLLWPLYGLCIADLSWLTFSATRTLLFNPDVTLDHNNNPEPWQAYREGRYRLWAGNYDYSKLKCKAPIFKDNDVIPVENGED |
| Aa-185869 | MFRRLQNSRNYLSYASDLLKKNFLYSLQLEANADADKWNQKRGQGVFTISAIPRPFSIRLAKSQDEPHIMHFIRENFYDEEPLIKSLNINKSLANPCLEEYLCNHLKAGFTLLAVEKDNRIVGISVNQRNCAWDGDRLREHADRVQCDPLRKLFYIWSIVSKEPRLHQKFKTPCIFEIAILATAREAQRQGIGYQLTMHSLRLARDLGFDVARMDCTNEYSSRLAQRAGMECMWSVPYKHLVDCNKKPVVKPGHPVC |
| Aa-187893 | ISFSSANARDLIQSLALTVPEKATATRTSGDDWQQQQQPSSSGAGGARPRHSRETDNRRILKMKQQVAKQNNFIAELKKKIRELASMPSKSIADHENLAFLKSRLDKENQLLKGLVDRLIKEQKTADSPGWEQIRLCTDPLEDICRNPWMLGNLPMPDQRFSFDSTLSSLSSKAQVDGESVEHVCDLDDNIKKELMNRDRVIEILQARVEALTADVMKVKRDNNAILDKTPKQTKFCEADIFNRLKFYKENTDALEKNLKQMDAALGVIRTELGPALTGECQESAGCRTFFTSSNGGQKSTGEVPRSSLASKQDDEQYNILMKEFSKKNEECQKLTDRLAKSCSCRNETPEQLEADVLKKRCSELLDIQEEFKILIKEQGEQ |
| Aa-190404 | MAHNRIFMVELIVEDLQMFSAEQLGGSSSPPPEEVQPQPTDEEAGESESSPTCPERCIRFQLSNLARCEVCEKDFGSQLDESSAKLGENCMFTLNVDGLKENELSFEISALEKRKDGGKKVLGKWVEPANELLDNLAKNFDNVNGRRGSEISIGTALSDDSVSKKNYPVSETIRSLYPLRDEQEVVRGCVIVTVRISCLGTRINQKVKLGAKDDQPGVTCFKMTDQEGEEQFMKCVAFDEVAHPHPVMCEECEKPPSMISLPPTPPGSVCSKEEVCAPYDEYTAEMNGNAISIRVEKDSEIKVMLDDEQDNSCTKGCWTSLKLPEGIYALEERFVQREENACRLPVIRGNLKYPARQWSADFMMCKQKRPICAEDYRKRPDPTRSICMQTRDPEEPHSVHPCGIEICKKGWQDPNVDVFVLKLGKNKTTRSQNGGNQIELELRTPKGPMREKRPKETRGVQVIESEFEDFKPPQAEDSKKPKKKASGKKGAKKK |
| Aa-190462 | MSSTSTPNAIDDSEPLAGIINTIDNLENTLSNLELDVRNELNTQRLLYRCQLNLAGRCPSVDRVRDTDSQSSQGTGSIVSKFSGCNCRCNQALEIYLGQLRRAQAEQEELLRVMKMKDEQAKLYRCKLMESNATVERQKQEIKALKDNEELITQKINTALEEENQTLMTEIERLKSLPDELRARERALKVANKELQETKLTLKSLLLDIESGLETCEDISGELQQERKRAFNTLNEIDEEKRKVLNWIAKYSELKQQYDSAVQQKESVAQLSTALREKTTKLEALTKDYDALRKESVDYISNVETVNEKQRTELQERVVELECQN |
| Aa-193018 | HTQEEFKILIKEQGNQLDEYRLKYLSAQQKVEEQKLEMGRMDVTNRRIEEQINIEVQRIKAKFQDKLRQLTPFPRLLEAEEEKVSKLKDSNEKLLEELKKSAKEIKSLEYRLHNAHASQNTELEKAHNLLQVELEQLQASLQSEREKNAKLHTQLEEAQKEIDDTRTETAKMIARTNDRAQEERKTALTRIEGL |
| Aa-195145 | MVACFSEDKNVKVPNFSDFRKSFLYTPCNCPLVDCEFSGQQRKVSTTRKYRTSRRIRKLAKPKCRGAKFYQRPVSQYGRTIQMIRAYQEPHASTRIQQLALPKVRKLIAARDAYRRFINRCWYDRFGKRIKRSMFTVYSRLANVHLPSTESKLIKMTPEQWKQHQQWLSKNAQPKPLKKPETKSRKRMPLIQLLDRVMDLSTPRWKCNKYDGPAKFRAVLPGALKATASDRVKALCQPKERHRRAKGPYKEDLSIPKGALEAVATQRVLDLAQPKIYRNVKNEYRENPFMVDPRALKAKAGDRILELAKPKKVKK |
| Aa-19539 | NQRSSPSTSSPSRSNSKTRSSSSKMSDRKAVIKNADMGEEMQQDAVDCATQALEKYNIEKDIAAYIKKEFDKRYNPTWHCIVGRNFGSYVTHETRHFIYFYLGQVAILLFKSG |
| Aa-198828 | ATPKPGTSKSNISRANASQITEPSDHSLDTEFHYYCALLHELIPNLRNPDEQEHANRWLTKLVEPSLNVRNLRDKRNRFLMMLCICLLSGHIQAPFNAAPVTKLPEVSSIKRPTFAPPAWHVSAAEWKDHLLLLSELYKKLKIPAIRKCQSHVKQCTGGKDARSTFLDKQFEFFLYLTKYYMHSLTSYPELRIACKRIEVLSQIDRNCCARAKGIRNDHMLVLMSYLLQHQLMGPFRRLPTHPLEPLMETARKVAKNKPMNRSDGLGINSGDDFLSQFPIPEEGAYAFISLTSDLFEKA |
| Aa-20177 | MSLLVARRVGNLISVRYARLLSTHTTLLTKENFDYDLVVIGGGSGGLACAKEAVQFGAKVAVLDFVKPSPRGTKWGLGGTCVNVGCIPKKLMHQASLLGEAIHDAQPYGWKFAEPESVKHDWATLTESVQNHIKSVNWVTRVDLRDKKVEYVNGLGYFKDAHNVVAVMKNQTEKVLSAKNVVIAVGGRPRYPNIPGAMEYGITSDDIFSLPNEPGKTLVVGAGYIGLECAGFLKGFGYDATVMVRSILLRGFDQQMATMVGDAMVEKGVKFLHKTQPQSVEKQADGRLLVKYRSDDGTEGSDVYDTVLFAIGRTACTGDLKLDQAGVVTAEGGKSDKLDVDSFETTNVPNIFAVGDVLYKRPELTPVAIHAGRLLARRLFNNQDDIMDYTDVATTIFSPLEYGCVGMSEENAEAKFGKDNIEVYHAYYKPTEFFVPQKSVRYCYLKAVALLEGDQKVLGLHFLGPVAGEIIQGFAAALKSGLTMKILKNTVGIHPTVAEEFTRLLITKSSGLDPTPATCCS |
| Aa-206597 | WSLIRNRLRVAPCSSIRRQHTIPKELEQVAKEKDPKFSKMIQYFFHKACVKLEPRLLGYLKKYPQLSEENRKKRVETIIHLVEGAANTIEVRFPVMRDNGQYEILTGYRSHHCVHRLPVKGGIRYSMDVTRDEVKALSSLMTFKCSCVHVPFGGAKGGIKLNPKSYSDKELQSITRRYTAELAKKNFIGPGIDVPAPDMGTSDREMSWMADQYSKTFGHKDINALATVTGKPLHQGGIRGRTEATGRGVFIATNCFVREKDWMNAIGLEPGMEGKTVVIQGYGNVGMYAECV |
| Aa-206656 | MAAGPALETKLDEDFLFILSFARSSMYLFQSRPVEQGLIESWFEKLCLEIYRGIDAKRQRNLYLVKLVTCVQSGILTDPFLAKPPAGALDPLPQTIAPSNLDEPPWLKDFEAAEAAAMPIAGGAKDFCSYLCTKQLDDGKGLCAYLAVSVADEGEKPRWFEMGSGRPLLLSELDEEIETAFQDFMGRVVEEGVLEESGGLGEYSGKLLDAIRRELDGQAPVGEDVYLDNLLAELEAHMANKSMGRELETYNQAQRRAFLLGYLKKKLELQLDERGYIY |
| Aa-206732 | MDSTVQPRMSLRLRYRPHLITDQQQLVSLLPTPKTPNITTVNESRRPLPANASITASALATVDRTGWNSRVDHALNEIIRGHHGYKPDQSVYGDINRMFDATLCTLILIASGDLNISSEHLLWSEAFLHGIRDRYGKHPPNVRKLLGFIKHIQGMVAAGKNGSMAERCGLFARTYQDALPDNVTLTRIKSPTGNATV |
| Aa-21748 | MAXSRIYSSKLASANKNLLPVITXXXXXXXXXXXKAVLSEKIPKEQERVKNFRKQHGATGWRSHRRYDVRGMRGIKGLVCETSVLDPDEGIRFRGLSIPECQKVLPKAPGGAEPLPEGLFWLLITGDVPTKAQVDALSREWANRAALPSHVVTMLNNMPTTLHPMSQLSCAVTALNHESKYAKAYSEGVHKSKYWEYVYEDSMDLIAKLPVVAATIYRNTYRDGKGIGAIDPKKDWSANFTKMLGYEDEQFTELMRLYLTIHSDHEGGNVSAHTVHLVGSALSDPYLSFAAGMNGLAGPLHGLANQEVLVWLQKLRKELGDNASEDKVKDFIWKTLKSGQVVPGYGHAVLRKTDPRYTCQREFALKHLPNDPLFQLVSNIYKVVPPILTELGKVKNPWPNVDAHSGVLLQYYGLKEMNYYTVLFGVSRALGVLASLVWDRALGLPIERPKSMSTDGLMKSVGAK |
| Aa-21906 | SACYHIITQLMDTLSKDVGFRTESDTFGELKVPNDKYYGAQTVRSTMNFPIGGPTERMPQPVITAMGILKKAAALVNKEYGLDPKIADAISLAADDVISGKLYDDHFPLVIWQTGSGTQSNMNVNEVISNRAIELLGGTLGSKAPVHPNDHVNKSQSSNDTFPTAIHISVARELTGNLKPAIQTLHDALKAKSNEFKDIIKIGRTHTQDAVPLTLGQEFSGYVQQMEFALQRIESVLPRVYMLALGGTAVGTGLNTRIGFAEKCAAKISELTGLPFITAPNKFEALAARDAMVEVSGCLNTIAVSCMKIANDIRFLGSGPRCGLGELSLPENEPGSSIMPGKVNPTQCEAMTMVCAQVMGNNVAVTVGGSNGHFELNVFKPLVVSNVLRSIRLLSDSARTFSKNCVVGIEANRANIDKIMNESLMLVTALNPHIGYDKAAKIAKTAHKEGTTLKQAALKLGYLTEEQFNEWVRPENMLGPK |
| Aa-22565 | MLRVLLKGLPKPGVGSQIARYSSIPAPKTSPEILYTGIFINNEWHKSIGGKVFPTLNPANEQVIAEIQQGEKADIDVAVGAAREAFKLGSPWRKMDASKRGQLLYRLADLMERDRVYLASLETLDNGKPYFMSYNVDVPMAINNLRYYAGWADKNHGKVIPMDGEFFRVHPSRTRGSLWTNHPVELPDFDGSLEVWTSPGNWKHNRPKTRRTDQLNGAVHGSVGQRGRIPSRCC |
| Aa-22566 | LMERDRVYLASLETLDNGKPYFMSYNVDVPMAINNLRYYAGWADKNHGKVIPMDGEFFVYTRHEPVGVCGQIIPWNFPILMAAWKFGPALATGNTIVLKPAEQTSLTALYMAQLVKEAGFPPGVVNVVPGFGDAGAALVQHDDVDKVAFTGSTEVGKKIQQGAGLSNLKRTTLELGGKSPNIILSDADMKHAVETSHFGLFFNMGQCCCAGSRTFIEDKIYDEFVERSAERAKKRTVGNPFDLTTEHGPQVDKAQYDKILGLIDTGKKQGAKLVAGGKKYEGLPGYFIEPTVFADVKDDMTIXXEEIFGPVQQLIRFKSLDEVIERANKSEYGLAAAVFSKDIDKVNYLVQGLRAGTVWVNTYNVLSAQAPFGGYKMSGHGRENGEYGLQAYTEVKSVITRIPVKNS |
| Aa-22567 | GRPKTNDTVGGKSRSPTLNPANEQVIAEIQQGEKADIDVAVGAAREAFKLGSPWRKMDASKRGQLLYRLADLMERDRVYLASLETLDNGKPYFMSYNVDVPMAINNLRYYAGWADKNHGKVIPMDGEFFVYTRHEPVGVCGQIIPWNFPILMAAWKFGPALATGNTIVLKPAEQTSLTALYMAQLVKEAGFPPGVVNVVPGFGDAGAALVQHDDVDKVAFTGSTEVGKKIQQGAGLSNLKRTTLELGGKSPNIILSDADMKHAVETSQCV |
| Aa-23276 | MAATARLFSRVKDGFTTSVRFASYESTRKNLKLNSNSKVICQGFTGKQGTFHSQQALEYGTKLVGGVSPGKGGRQHLNLPVFNSVKEAKAATGADATVIYVPPPGAANAIMEAIEAEIPLVVCITEGVPQHDMVKVKHALLAQDKSRLVGPNCPGIIAPEQCKIGIMPGHIHKRGKIGVVSRSGTLTYEAVHQTTEVGLGQTLCVGIGGDPFNGTDFIDCLEVFLKDPDTKGIILIGEIGGVAEEKAADYLMQYNQGIKAKPVVSFIAGLSAPPGRRMGHAGAIISGGKGGAQDKINALEKAGVIVTRSPAQMGKELFKEMKRLELA |
| Aa-23516 | MSAPEGSSNGTQPAASSANGATANRLKQTQAQVDEVVGIMRVNVEKVLERDQKLSELDQRADALQHGASQFEQQAGKLKRKQWWANMKMMIIMGIIGVVLLIIIILSIVY |
| Aa-23719 | MAPRKNKTVKEEVQVSLGPQVREGEIVFGVAHIYASFNDTFVHVTDLSGKETISRVTGGMKVKADRDEASPYAAMLAAQDVAEKCKSLGITALHIKLRATGGNRTKTPGPGAQSALRALARSSMKIGRIEDVTPIPSDSTRRKGGRRGRRL |
| Aa-24110 | MAFGDYPAEYNPKVHGPYDPARFYGKADTPLGQVKLSELGAWFGRRDKNPRAAAGAVSRAFWRWQHKYWQPKRMGIAPFFQVIVGGMVFFYTINYGKLKHHRNYKYH |
| Aa-25258 | MHPLLVQQFANKFSLLKQLGRSTATVQRFSFHTSKMVQIKEGDKIPSIDLFEDSPANKVNMADLCAGKKVVLFAVPGAFTPGCSKTHLPGYVDRADAIKSSGVQEIVCVSVNDPFVMSAWGKQHNTGGKVRMLADPAAVFTKQLELGADLPPLGGLRSKRYSMVLEDGVIKSLNVEPDGTGLSCSLADKIKV |
| Aa-25408 | MASLLKLSSTLRVCGNGTKLIQRLKSSNAADFRAALVNVPPTEVTQLDSGLRVASEDSGSQTATVGLWIDAGSRYEDARNNGVAHFLEHMAFKGTAKRSQTDLELEVENMGAHLNAYTSREQTVFYAKCLSRDVPKAVEILSDIIQNSKLGEAEIERERGVILREMQEVESNLQEVVFDHLHATAYQGTPLGNTILGPTKNIQSIGKSDLQAYIDSHYKAPRIVLAAAGGVKHNDLVKLAQSSLGKVGSTFDGKAPQLSACRFTGSEVRVRDDSLPLAHVAIAVEGCGWTDQDNVPLMVANTLIGAWDRSQGGGTNNASKLAAAAAEDNLCHSFQSFNTCYKDTGLWGIYFVCDPLKCEDMVFNLQNEWMRLCTMVTDSEVDRAKNLLKTNMLLQLDGTTPICEDIGRQMLCYNRRIPLHELEKRIDNVNAQNVRDVAMKYIFDRCPAIAAVGPIENLPDYMRIRASMYWLRV |
| Aa-25662 | MPSDKTIGGGDDSFNTFFSETGAGKHVPRAVFVDLEPTVVDEVRTGTYRQLFHPEQLITGKEDAANNYARGHYTIGKEIVDVVLDRIRKLADQCTGLQGFLIFHSFGGGTGSGFTSLLMERLSVDYGKKSKLEFAIYPAPQVSTAVVEPYNSILTTHTTLEHSDCAFMVDNEAIYDICRRNLDIERPTYTNLNRLIGQIVSSITASLRFDGALNVDLTEFQTNLVPYPRIHFPLVTYAPVISAEKAYHEQLSVAEITNACFEPANQMVKCDPRHGKYMACCMLYRGDVVPKDVNAAIATIKTKRTIQFVDWCPTGFKVGINYQPPTVVPGGDLAKVQRAVCMLSNTTAIAEAWARLDHKFDLMYAKRAFVHWYVGEGMEEGEFSEAREDLAALEKDYEEVGMDSGEGEGEGAEEY |
| Aa-25682 | MSDVQDLMSSLPDDKIDMIAATSVLQQQAGDIRQNKPNWSSYKQSQMISQEDYACVSSLDKDKKSQAQYLQENPGQCAKTFLNLLSHVSKDQTIQYILVMIDDLLQEDRTRVQLFHDFANKRKESVWAPFLNLLNRQDGFIVNMASRVVGKLACWGQELMPKSDLHFYLQWLKDQLTVANNEYIQSVARCLQMMLRVDEYRFAFVTVDGISTLISILSSRVNFQVQYQLVFCLWVLTFNPLLAEKMNKFNVIPILADILSDSAKEKVTRIILAVFRNMIEKPEDAQVAKEHCIAMVQCKVMKQLQILEQRRFDDEDISADLEFLIEKLQNSVHDLSSFDEYATEIKSARLEWSPVHKSAKFWRENAQRLNEKNYELLRILVHLLETSKDPLVLSVASYDIGEYVRHYPRGKHVIEQLGGKQLVMQLLGHDDPNVRYEALLAVQKLMVHNWEYLGKQLEKESEKTPQSGAAISGKA |
| Aa-25821 | RHSHLLLWQASIQVPAQSSQSFTFPRVRLYSVSLVKMAVTGLTCLSTLLLLAAILLLSVNGNTHLVHRRFEYKYSFKPPYLAQKDGTVPFWEYGGNAIASSENVRIAPSLRSQKGAIWTKQRTNFDWWEVDIVFRVSGRGRIGADGLAFWYTTEKGDYTGEVFGSSDRWVGLGIFFDSFDNDNKHNNPYISAVLNDGTKKFDHTNDGATQLLSGCLRDFRNKPFPTRAKIEYYNNILTVLFHNGMTNNDQDYEMCFRAENVVLPKTGYFGISAATGGLADDHDVFHFLTTSLHVPGQIKEEVPHDQDQAKLTQEYQDYQKKLEQQKEDYQKEHPELQKDDLENWFEDYNARELRQIWEAQTATHDQLRTLNTKLDEVIGRQERTLGLLSVQGGGGVPPPPQQGGAVPQIGTGSVIQRHEVDALLRNQNVMVQTVTEIRSIIGEVHARADSILNNQARAPTAQIQGGGYDIQSLMHEMRDGMNQVKQGMAGVSQRLQQPNAQANVQCPTQNCVGMTAFLVTIVVHLLVIVGYNMYRDSRDAQAKKFY |
| Aa-26406 | MLSRAALLAAAKKPVGLVMARGSASATDSNRPVRAEHPGKVRMGFLPEEWFTFFYNKTGVTGPYVFGAGLLTYLCSKEIYVMEHEYYNGLSLAIMVIYAVKKFGPAVAAYCDKEIDRIEGEWKADRENNIQQLAQAMEDEKKEQWRAEGQTLLMQAKKENVALQLEAAYRERAMTVYREVKKRLDYQVERQNVDRRISQKHMVDWIVKNVVKSITPEQEKETLSRCIADLGAIAARAK |
| Aa-26445 | MQCNIRHLVNLAAKGNAAAGLRNHGAILGALYGRFYSTTHDADLVVIGSGPGGYVASIKAAQLGMKTVCIEKNDTLGGTCLNVGCIPSKALLNNSHYYHMAHSGDLASRGVLVDNVRLDLSVLMDQKSKAVKSLTGGIAQLFKKNKVTHVNGFGTITGPNTVVAKMADGSEEVVNAKNIMIATGSEVTPFPGIEIDEETIVSSTGALKLKEVPKRMGLIGAGVIGLELGSVWGRLGAEVTAIEFLTSIGGAGIDQEVSKSFQKILTKQGFKFLLGTKVVAASKSGNGVTVSVENVKDGSKQELEFDVLLVSVGRRPYTEGLGLENVGIVKDDRGRVPVNSVFQTIVPSIYAIGDCIHGPMLAHKAEDEGIVCVEGMQGGHVHIDYNCVPSVVYTHPEVAWVGKNEEELKNEGIAYNVGKFPFMANSRAKTNNDTDGFVKVLADKQTDRVLGVHIIGPVAGELINESVLAMEYGASAEDVARVCHAHPTCAEALREAHTAASFGKPINF |
| Aa-26579 | MSSGVGSGQSCGEPASSSTQGSYVSAGGSKSESVTVRTMDGSHQSEAQSVTLTVDGFGTVKDPLTDEVQPVKRFTWTNESGMSVQVISYGAIITSIKVPGKNGAVDDVVLGFDNILGYRGANNPYFGATVGRVANRIGGGRFTIDGVVYEVTKNWEGRHQLHGGKIGFDKFNWTSHVEGTVVTLSHTNKDGHEGYPGTVLASVTYELKNDNRLVVKFRAVSDKPTPINLTNHSYFNLAGHNTGHEEVYRHIISLNADRITETDEDSIPTGKFLCVGGTPYDLRIPRELGPAMSRAPGEGYDNNFCITKGTEQGMTFIARVVHPHSGRTLEVYTDQPGVQLYTSNFMPDPNRNIRPRPINAGDYYELTHLEPVVPAMATDLPIRGKGGAKYFKHGAFCLETQNFPDAVNHANFPNSVLVPGETYEHEVVYKFGLFEEN |
| Aa-28076 | MLRSAAGQLFGVLRGTVGLTTSRLGAAGAVRHSHSNESAEEFDARYEAYFSRSDIDHWEARKGMNDLLGMDLVPEPKVIIAALKACRRLNDYALAIRFLEGCKDKCGNQVNQIYPYLLQEIRPTLTELGIDTPEELGYDQPELALKSVFDMH |
| Aa-28226 | MADTSSHLSWLIIRDHNAFLLKRRNIKKPFSTEANNLTNLSSFRYSGLVHKKSLGVVPADKGIQVVYKRPKYQTKPAKATVKVTLKHRPRRTLKKLKNIVNENRYRRDLRQAALRRASAILRSQRPASAKKGKAAAGAGAPAAAAAAAPKKGRISGRSPKGNREP |
| Aa-28638 | LPLYRCKRDRPCSQRVLFRRLCLQVVAAVVVVPFAAQATEIVRKVMAKKRPKPPKTNAAAKAPPPPPPPPADIDGEKELFANMDDPAYTDIRAAKPAKPPAAAAAAGGGGPLPGVYPTPAPAAGKKPANAHDGVGPVRYETAPGDPPPKMDPPPMAPGADKAKVVEPVYWTD |
| Aa-28702 | MFAVCKSGGLLVSSMQRYGVRCVSSVVPDVKLPEYPLVNEPVLTYKKGSKERKELEAALKKTASAVEDVPIIIGSEEFRTNNVRHQVMPHNHSQKIAQFYWADKKLVEKAIKTATETQVKWDRTPISERVKIWQKAADLMAGPYRAELNAATMLGQAKTVIQAEIDSAAELIDFIRMNTVYLKEASKYQPISENSKVTKNSLRFRGIDGFIAAVSPFNFTAIGGNLAYTPALMGNGVLWKPSDTALLSNYVIFKIMREAGVPPGVVNFIPTDGPVFGDTITASPHLAGINFTGSVPTFNRLWRQVGENINIYNNFPRLIGECGGKNYHFVHPSADVQSVVNGTIRSSFEFCGQKCSACSRMYVPESLWPQVKEGLIKTRDTLKIGDVTDFSTFTSAVIDDKAFSRIKSYIDHAKSSKNLQILAGGKCDDSKGYFIEPTIVQSTDPKDKIMTEEIFGPVLSIYVYKDKDLDQTMKLVGNSTRFALTGAVFSKDEGFLKRALEEFKLTAGNFYLNDKSTGSVVGQQPFGGGRMSGTNDKAGGPHYVLRWSTPQSIKETFVPLNEVDYQYMRE |
| Aa-29288 | MFHLKTIAKSAALKQEVSTLVKAATAAGAQQVSQQQRPYSSQHQIPERLKDVGTAASPRFFDMVEYFFHRACQICEEKMVEDMKGRISLEEKRKRVKGILMLMQPCDHIIEIAFPLRRDSGDYEMITGYRAQHCTHRTPTKGGIRFSLDVSRDEVKALSALMTFKCACVDVPFGGAKAGVKIDPKQYSEHELEKITRRFALELSKKGFIGPGIDVPAPDMGTGEREMSWIADTYAKTIGHLDINAHACVTGKPINQGGIHGRVSATGRGVFHGLDNFIKEANYMAMIGTTPGWGGKTFIVQGFGNVGLHSCRYLCRAGATCIGIIEHDGSIFNPQGIDPKALEDYRNEHGTIVGFPGAMPYEGENLMYEPCDIFIPAAIEQVITSENANKINAKIIAEAANGPTTPAADKILIDRNILVIPDLYINAGGVTVSFFEWLKNLNHVSYGRLTFKYERESNYHLLESVQASLERRFGNVGGKIPVTPSEAFQKRISGASEKDIVHSGLDYTMERSARAIMKTAMKYNLGLDLRSAAYVNSIEKIFQTYRDAGLAF |
| Aa-30265 | FARSLKTVATQGVKNFSTTSQNNVKVAVCGASGGIGQPLSLLLKQSPLVTELSLYDIVHTPGVAADLSHIETHSKVTGYNGAENLEKALANADIVIIPAGVPRKPGMTRDDLFNTNASIVRDLAAGCAKACPKALIGIISNPVNSTVPIACETLAKAGVLDVKRVFGVSTLDIVRANTFIGEAAGVDPQKVNVPVIGGHSGVTIIPVLSQATPSVSFPQDKIAALTERIQEAGTEVVKAKAGAGSATLSMAYAGARFALALARAMKGEQNVVECAYVRSDVTEAKYFSTPLVLGKNGLEKNLGLPKLNAFEQELLKKALPELKKNIQKGEDFVNKK |
| Aa-30534 | MVHYARLMNVQAKRAALYAVESQQRYFHASCASAAKVALSRFDQDVYLPYEKLQKNLEVVKKRLNRPLTLSEKILYGHLDDPANQDIQRGTSYLRLRPDRVAMQDATAQMAMLQFISSGLPRVAVPSTIHCDHLIEAQVGGEKDLARAKDLNAEVYKFLSTAGAKYGVGFWKPGSGIIHQIILENYAFPGLLMIGTDSHTPNGGGLGGLCIGVGGADAVDVMANIPWELKCPNVIGVHLTGKISGWTSPKDVILKVADILTVKGGTGAIVEYYGKGVDSISCTGMATICNMGAEIGATTSTFPFNQRMADYLASTGRAAIAGEAKKFQQSVLTADSGAKYDQVIEINLDTLEPHVNGPFTPDLAHPISKLGANSKKNGYPMDIRVGLIGSCTNSSYEDMGRCASIAKNAMKHGLKSKIPFNVTPGSEQIRATIERDGIAKTFKEFGGTVLANACGPCIGQWDRKDVKKGEKNTIVTSYNRNFTGRNDANPATHCFVTSPELVTALSIAGRLDFNPLTDELTGKDGKKFKLEAPFGDELPQKGFDPGMDTYEAPPSDGSKVKVDVDPKSQRLQLLEPFDVWDGKDLTEMTVLIKVKGKCTTDHISAAGPWLKYRGHLDNISNNMFIGATNIENNEMNKIKNQVTGEWAGVPDVARFYKAKGIRWVAVGDENYGEGSSREHAALEPRHLGGRAIITKSFARIHETNLKKQGLLPLTFANPADYDKVQPHSKISLLGLDKMAPGKQVDCEIKTDGKVDKIKLNHSFNEQQIAWFKAGQCAEPHEANCSGQD |
| Aa-32597 | MALAFITRTGAFVGLARSKPSFSGGVSGGSLLVQQVCNYAKKAMGLPRCFFDLTADNAPLGRVVIELRTDVTPKTCENFRALCTGEKGFGYKGSTFHRVIPNFMCQGGDFQNHNGTGGKSIYGNKFEDENFILRHTGPGIMSMANAGPNTNGSQFFITTVKTSWLDDRHVVFGSVVEGMDVIRKVESYGNQSGKTSKQIVVANCGQL |
| Aa-32890 | MKLLVVLSVLLVGALAQDGPKVTDKVFFDITIGGKPEGRIVIGLFGGTVPKTAKNFKTLAENAEGEGYKGSKFHRVIQDFMIQGGDFTRGDGTGGRSIYGERFADENFKLKHYGAGWLSMANAGKDTNGSQFFITVKQTSWLDGRHVVFGKVLEGMDIVRKIEKTKTDGRDRPVNDVVIADSGSLPVDAPFSVEKADA |
| Aa-33345 | MASILRKSGLVLADLAAKSSPRVLGSASQKRHLNVQEHVSYTFLNEAGIPTPKFGVANSAQDAEKIAKGLNTKNLVLKAQVLAGGRGKGTFKNGLKGGVRVVFSPQEAREISGKMIKQLLVTKQTGAAGRICNSVMVAERKFPRREFYFAVMMERAFNGPVLIASSQGGVNIEEVAAENPDAIVYEPIDIKNGLQKAQAVAIAKKVGLEDHAEETAKMLLNMYDLFVKKDALLIEINPYAEDAGETYFALDAKMRFDDNAEFRQKDLFAKRDLSQEDSKEVEASKFDLNYIALDGSIGCLVNGAGLAMATMDIIKLHGGDPANFLDVGGGASVKAVKEAFRIITSDPKVHAILVNIFGGIMRCDVIAEGIIQATKELNIKMPIIVRLQGTNVNEAKELIKKSKLRILPKDDLDEAAMLSVHLAQIVHLAREAHLDVSFELPDSYIQ |
| Aa-34188 | GCARHKNSGVEFNTSGSSNQDNGKVFGSLETKYKVKEYGLNFSEKWNTDNTLTSEVSVENQLVKGLKLSFDGSFAPQTGSKTGRFKTAYSHDKVRVDADVNVDLAGPLVNASGVFNYQGWLAGYQVAFDSQKSKVTANNFALGYSTGDFVLHTNVNDGREFGGLIYQRCNDRLETAVQLSWASGSNATKFGLGAKYDLDKDACVRAKVNNQSQIGLGYQQKLRDGVTLTLSTMIDGKSFNTGGHKIGVALELEA |
| Aa-34693 | MASLAAKGSSLVSTLLTNARPKFNVFMKYAKVELTPPTPADIPAIRDGIARIVSGARTGAWKNLTVKEAWLNTLVTAEVCFWFYVGECIGKRHLVGYEV |
| Aa-34714 | ALGDADHAHTIVSIDRKLQRVIMSGAIKKATGLTGLHVAKNPHHTLTALYNKILRAVAKMPQDAAYRRYTEQIVSERAKVVATTPSVREVEDKINCGQVEELILQAENELTLARKMLGWKPWEPLVKQAPATQWSWPPAKIHELK |
| Aa-3560 | RRTSSMXKYSQLTSSNVDSKLREDLERLKKIRANRGLRHYWACVCAVSTPRPPVAVDVPYGVSKKK |
| Aa-37140 | MSAKTAAMIAHTRRVCSLYKKSLRNLESWYDRRHIFRYQAVIMRERFDKHKNERDPAKIAQLLADGERELFETQHFQPKKFPMSPGGVAFEREVIPPDWVLDYWHPLEKAQFPEYFARREKRKEEYVAWWEKQYGKTSASEGSHHH |
| Aa-3755 | MAFLSRSVLLLLVICQIVFRKASAQANNAPLPPPGPGDLGVNQGMKLTDPLTFNQPPMPSANSNTLNLPASAAAASSNQLAPSLPFGVPRSSISSFGAPPSANSGMMNTPASPMDLQATATTTNDARSEIRYCYANGPLLPSSLLPKPRVETHIDVGSVSCEEIVGYLMGLNRWYDRKCGTMGPECPHKFRYAVGWFSAAVKQIKGKCNLTL |
| Aa-38566 | MMSSLALRFAPRFGKSTAAAVASSSSQQKFNTYYTYSNQISQPLERKPTYTTAEEAVKCIKSGDVVFVQGAAATPIHVLDAMTKHGKENNLRDITVVHMHTEGPASYCAPDCKDIFRSKSLFMGGNVRAAVADGRGDAVPIFLHEIPILFRRKLIQPDVAIVSVSPPDNHGYGTLGTSVDCVRAALENSKVIIAQVNKQMPRTFGDSIIHESHFDAAVNVDIPLPEHGGKGMSDTETMIGKLIAENLVEDGATLQMGIGNIPDAVLNALHNHKDLGIHSEMFAGGVVDLVKKGCVTNDKKTYHRGRIVGSFLIGTKKLYDFVDNNPFIEMLEINYVNNVGIIARNPKMTAINSAIEVDLTGQVCADSIGTRMYSGFGGQVDFIRGAAEGFDGKGKPIIALPSITNKGQSKIVPTLKPGAGVVTSRAHVHYVVTENGIANLFGKSLRQRAYELIQIAHPDHREALEKAAFERLKTMPSPN |
| Aa-41181 | MFRLPTVLRCTAARQVAAGYRGYAKDVRFGPEVRALMLQGVDVLADAVAVTMGPKGRNVILEQSWGSPKITKDGVTVAKGIELKCKFQNIGAKLVQDVANNTNEEAGDGTTTATVLARAIAKEGFEKISKGANPVEIRRGVMLAVDAVKDHLKSMSRAVTSPEEIAQVATISANGDRAIGDLISEAMKRVGKDGVITVKDGKTLHDELEIIEGMKFERGYISPYFINSSKGAKVEFQDALVLFSEKKISTVQSIIPALELANSARKPLVIIAEDVDGEALSTLVVNRLKIGLQVAAVKAPGFGDNRKSTLSDMAISTGGIVFGDDANLVKLEDVQMSDLGQVGEITITKDDCMMLKGKGDAKHVEGRVDQIRDQIAETTSEYEKEKLQERLARLSSGVAVLKIGGSSEVEVNEKKDRVNDALCATRAAVEEGIVPGGGTALLRCIKTLENLKGSNDDQKAGIDIVRRALHQPCTQIAKNAGVDGSVVVAKILDQQGDFGYDALNGEYVNMIEKGIIDPTKVVRTALTDASGVASLLSTAECVVTEEPKPEGAGGGMPGMGGMGGMGGMGGMGGMM |
| Aa-41828 | MSVIGIDFGNESCYVAVAKAGGIETIANDYSLRATPSCVAFAGRNRVLGVAAKNQQVTNMNNTIGGFKRLLGRKYNDPHVQNELRKIPYKVEPRPDGGIGIRVNYQDEECVFSPEQITAMLFTKLKDDSFKELKAQINDCVITVPSYFTNAERQALLDAAGICGLNVLRLMNETTATALSYGFYKQDLPAPEEKARNVIFVDCGHSALQVSACAFHKGKLKMLASCADQVGGRDFDYALAEHFSNEFQTKYKIDPRTNKRAYLRLLTEAEKLKKQMSANSTKLPLNIECFMNEIDVHSTIQRGDMETMCAGSLQRIEATMKKLLKDSNLALEDIHSVEIVGGSSRIPAIKQLIEQIFGKTASTTLNQDEAVSRGAALQCAILSPAVRVREFTCSDVQAYPVLISWEDGTQRNEMKVFEQYHTAPFSRLLTVHRREPMTINVHYEPNSVPYPDTFIGSFHIKDIKPNANGDPQEVKIKVRINQNGIVLVSSATMVEKRESEEPVTPPATANGEQPAQTNSPQGEESPKTGEPMDIQEDKKKKVTTKSIDLTIDGKTHGFVSNDLSKYHELEMKMIANDRQEKERVDARNALEEFVYEVRGKIQEDGELSAYVEQDEASKICLQLEDIENWLYEDGENCERTVYKDKLLALHKQTEPIRVRCEEYNGHEQAFTELGHTIQLTYKAVEQIRAKDPKYDHLTETEILNITEAAQKAQKWYEEARSKLVNVRKTQDPPVKVADIRHENQTLATCTNSVLNRPKPKPPTPPADNNKDQQQNGGDQAKDQQQADQSTEAPKDFTEDKMDVE |
| Aa-4267 | MKSFHITLVVALALVLALLMERTVAYSRDLINTDHPSRDIAGYLPPVSKDRVDPELFKRNRERTRSAVVKVRLPVSNRKKRVRPSRNNRATFGQRP |
| Aa-43642 | MIGNLAKLSYVRGVSQAVASGCKAAAVPVKLETKPAVAGQTPIVASSPNCALPRSVVRVVSGVAATGQIRLAHTDIQTPDFSAYRRDQVKRPNAKNDSADERAAFTYLMVGGAAVTTAYVAKSLVSTFVSSMSASADVLAMAKIEIKLADIPEGKSVTFKWRGKPLFIRHRTAAEIAAEEAVNTGTLRDPQHDSERVKNPEWLVVIGVCTHLGCVPIANAGDFGGYYCPCHGSHYDASGRIRKGPAPLNLEVPHYEFPEDGLVVVG |
| Aa-45445 | MAARLIKRIASTPFGARGYASGVKKVTLIPGDGIGPEISAAVQKIFTAANVPIEWEAVDVTPVRNPDGKFGIPQSAIDSVNRNKVGLKGPLMTPVGKGHRSLNLALRKEFNLYANVRPCRSLEGYKTLYDNVDVVTIRENTEGEYSGIEHEIVDGVVQSIKLITEEASNRVAEYAFKYAKDNNRKKVTVVHKANIMRMSDGLFLRCCREMAKKYPEIKFEEKYLDTVCLNMVQDPSKFDVLVMPNLYGDIMSDMCAGLVGGLGLTPSGNMGLNGALFESVHGTAPDIAGKDLANPTALLLSAVMMLRHMELTTHADKIQNACFETIREAKFLTGDLGGKAKCSEYTNAICEKIH |
| Aa-45778 | SEVTSKIPKPVLRGLHNASIKRNLAVAGVLCVVSVAAFNFLYVQPKQQAYADFYKNYDANKHFERMKNAGLLQSVKE |
| Aa-4593 | MSTVDKEELVQKAKLAEQSERYDDMAQAMKSVTETGVELSNEERNLLSVAYKNVVGARRSSWRVISSIEQKTESSARKQQLAREYRERVEKELREICYEVLGLLDKFLIPKASNPESKVFYLKMKGDYYRYLAEVATGETRNTVVDDSQAAYQDAFEISKGKMQPTHPIRLGLALNFSVFYYEILNSPDKACQLAKQAFDDAIAELDTLNEDSYKDSTLIMQLLRDNLTLWTSDTQGDGDEPQEGGDN |
| Aa-4716 | MQIFVKTLTGKTITLEVEPSDTIENVKAKIQDKEGIPPDQQRLIFAGKQLEDGRTLSDYNIQKESTLHLVLRLRGGIIEPSLRILAQKYNCDKMICRKCYARLHPRATNCRKKKCGHTNNLRPKKKLK |
| Aa-4735 | MFRAELNEFLTRELAEDGYSGVEVRVTPTRTEIIIMATRTQNVLGEKGRRIRELTAVVQKRFGFAPGTVELYAEKVATRGLCAIAQAESLRYKLIGGLAVRRACYGVLRFIMESGAKGCEVVVSGKLRGQRAKSMKFVDGLMIHSGDPCNEYVDTATRHVLLRQGVLGIKVKIMLPWDPNGKIGPKKPLPDNVSVVEPKDEIMYSTPRSEPKNKPTMELAEVVEPVAS |
| Aa-4904 | MSSETGRTDSYRVATVAPLKDDNRTADGQFKTRRKMPPKKKGDNLDDLKQELDIDYHKITPEELYQRLQTHPENGLSHAKAKENLERDGPNALTPPKQTPEWVKFCKNLFGGFALLLWIGAILCFIAYSILASTVEEPADDNLYLGIVLTAVVIVTGIFSYYQESKSSKIMESFKNMVPQFATVLREGEKLTLRAEDLVIGDVVEVKFGDRLPADIRIIEARNFKVDNSSLTGESEPQSRGPDFTHENPLETKNLAFFSTNAVEGTAKGVVISCGDHTVMGRIAGLASGLDTGETPIAKEIHHFIHLITGVAVFLGVTFFVIAFILGYHWLDAVIFLIGIIVANVPEGLLATVTVCLTLTAKRMASKNCLVKNLEAVETLGSTSTICSDKTGTLTQNRMTVAHMWFDNQIIEADTTEDQSGVQYDRTSPGFKALSRIATLCNRAEFKGGQEGVPILKKEVSGDASEAALLKCMELALGDVLSIRKRNKKVCEIPFNSTNKYQVSIHETEDASDPRYLLVMKGAPERILERCSTIFINGKEKLMDEEMKEAFNNAYLELGGLGERVLGFCDFMLPSDKFPAGFKFNSDEVNFPCENLRFVGLMSMIDPPRAAVPDAVAKCRSAGIKVIMVTGDHPITAKAIAKSVGIISEGNETVEDIAQRLNIPVSEVNPREAKAAVVHGSELRDLSTDQIDEILRYHTEIVFARTSPQQKLIIVEGCQRMGAIVAVTGDGVNDSPALKKADIGVAMGIAGSDVSKQAADMILLDDNFASIVTGVEEGRLIFDNLKKSIAYTLTSNIPEISPFLAFILCDIPLPLGTVTILCIVFGN |
| Aa-5040 | MNWRILLEREKKLRGDVEKAKRKVEGDLKLTQEAVADLERNKKELEQTVMRKDKEISALSAKLEDEQSLVGKTQKQIKELQGRIEELEEEVEAERQARAKAEKQRADLARELEELGERLEEAGGATSAQIELNKKREAELAKLRRDLEESNIQHEGTLANLRKKHNDAVAEMAEQVDQLNKLKTKAEKERSQYYAEMNDARLSLDHMANEKAAQEKVAKQLQHTLNEVQGKLDETNRTLNDFDSAKKKLSIENSDLLRQLEDAESQVSQLSKIKISLTQQLEDTKRLADEESRERATLLGKFRNLEHDLDSLREQVEEEAEGKADIQRQLSKANAEAQLWRTKYESEGVARAEELEEAKRKLQARLAEAEETIESLNQKCVALEKTKQRLSTEVEDLQLEVDRATSIANAAEKKQKAFDKIIGEWKLKVDDLAAELDASQKECRNYSTELFRLKGAYEEGQEQLEAVRRENKNLADEVKDLLDQIGEGGRNIHEIEKSRKRLEAEKDELQAALEEAEAALEQEENKVLRAQLELSQVRQEIDRRIQEKEEEFENTRKNHQRALDSMQASLEAEAKGKAEALRMKKKLEADINELEIALDHANKANAEAQKNIKRYQQQLKDVQSALEEEQRARDDAREQLGISERRA |
| Aa-50737 | MPAKAVCVLSGDVKGTIFFQQNGDSDAVKVTGEVTGLKPGNHGFHIHEFGDNTNGCTSAGPHFNPHGKEHGGPTAAERHAGDLGNVVADDSGVAKVDISDSQISLSGPLSILGRTVVVHADPDDLGLGGHELSKSTGNAGARLACGVIGICKA |
| Aa-53138 | MMLATAAVRGLTRRSFSTSKVLSAQQLTVRDALNSALDEEMERDERVFLLGEEVAQYDGAYKVSRGLWKKYGDKRVIDTPITEMGFAGIAVGAAFAGLRPVCEFMTFNFSMQAIDQVINSAAKTFYMSAGTVNVPIVFRGPNGAAAGVGAQHSQCFGAWYSHCPGLKVVSPYDSEDAKGLLKAAIRDPDPVVCLENEMLYGVGFPVSDQVLDKEFILPIGKAKIMRPGKHVTLVAHSKAVENALQAANELAGKGVECEVINLRSLRPLDSETIFKSVQKTHHLVTVEQGWPQSGIGSEICARIMEHETFFHLDAPVWRVTGVDVPMPYAKSLEAAALPQTHDVVTAVNKVLGIK |
| Aa-54066 | MSLVWSLIASFLYVEIFIVLMLVLPVASPQRWQRFFKSRFLAMLSRQAQTYFYLLLFVLVLFLLEAIREMRKYSHVDPAAEQHLNVGMQHSMRLFRAQRNFYISGFAIFLSLVIRRLISLITSQAQLLAQSEASMKQAQSATAAARSLLSQQKKEDEAGDKPKPSAPSADEVSADELKKRVAELESELARERKDKEAMKSQSESLNREYDRLTEEYSKLQRKIAISSNDKSD |
| Aa-54604 | MAQSKLNDLAGKFGKGGPPGLATGLKLLAAVGAAAYGINNSMFTVEGGHRAIMFNRIGGVGDDIFSEGLHFRVPWFQYPIVYDIRSRPRKISSPTGSKDLQMVNISLRVLSRPDALRLPTMYRQLGLDYDEKVLPSICNEVLKSVVAKFNASQLITQRQQVSLLIRRELVERAKDFNIILDDVSLTELSFGKEYTAAVESKQVAQQEAQRAAFLVERAKQERQQKIVQAEGEAEAAKMLGLAVSQNPGYLKLRKIRAAQNVARTIANSQNRVYLSANSLMLNISDAEFDDMSKKVSNK |
| Aa-5623 | MLKAAKFVTRALTENRALLQNGGILRNSMQARLKSDTVKGAVIGIDLGTTNSCVAVMEGKQAKVIENAEGARTTPSHVAFTKDGERLVGMPAKRQAVTNSANTFYATKRLIGRRFDDPEIKKDMKNLSYKVVRASNGDAWVQGGDGKVYSPSQIGAFVLMKMKETAEAYLNTNVKNAVVTVPAYFNDSQRQATKDAGQIAGLNVLRVINEPTAAALAYGMDKSEDKIIAVYDLGGGTFDISILEIQKGVFEVKSTNGDTLLGGEDFDHHIVDYLVAEFKKEQGIDITKDAMAMQRLKEAAEKAKCELSSSVQTDINLPYITMDASGPKHLNLKFTRAKLEQLVGDLIKRTIGPCQKALSDAEVSKSDIGEVLLVGGMSRMPKVQQTVQDIFGRTPSRAVNPDEAVAVGAAVQGGVLAGDVTDVLLLDVTPLSLGIETLGGVFTRLITRNTTIPTKKSQVFSTAADGQTQVEIKVHQGEREMAADNKMLGSFTLVGIPPAPRGVPQIEVVFDIDANGIVHVSARDKGTGKEQQIVIQSSGGLSKDEIENMVKNAEQYAATDKLKKERIEAINQAEGIVHDTESKMEEFKDQLPKEECDKLREEIVKVREILANKDEADPEEIRKTVSTLQQSSLKLFEMAYKKMASERESSGSSSSSSSSSDDSAEKKENKN |
| Aa-5792 | MSGKKADPYGFAKDFLAGGISAAVSKTAVAPIERVKLLLQVQAASKQIAADKQYKGIVDCFVRIPKEQGFGAFWRGNLANVIRYFPTQALNFAFKDVYKQVFLGGVDKNTQFWRYFLGNLGSGGAAGATSLCFVYPLDFARTRLGADVGRAGAEREYNGLIDCLKKTVKSDGLIGLYRGFNVSVQGIIIYRAAYFGCFDTAKGMLPDPKNTSIFVSWAIAQVVTTASGVISYPFDTVRRRMMMQSGRAKADIMYKNTLDCWVKIAKTEGSSAFFKGAFSNVLRGTGGALVLVFYDEVKALMG |
| Aa-59104 | MFKIISEAARGISVKNLEAAGKLAKARPFSSSSSNKDRGLTDFSQKKDCQQKACSDRSTKMPPKEPKSSCDKPVSPKTWRTCPEPPTPKEFSCADTVQEMVPRRKKRQVASRPACSKPAPSLAAPDCVKVKKELCPRASLPGCGKAKIPPRCDPKKVVRDCIRLRPPVACFSECYRNPFPPQPRSECTCLTTPRIC |
| Aa-5916 | MASAVSKTPMLRAAAARGFAAQAQAAAACRGSADVQCTNLPNKMTVASAESGAAVARVSIVYRAGSRNETADNLGASHVLRAAAGLSTKTATTFGITRNLQQVGSSLTATSDRETITYTVAVTKDELETGLKFLEAAATGQVFKPWELADLTTRVKADIARVPTEVEAVESLHKAAFHSGLGNSVFCPGYNAGKHSSETMQHYVAANCTAARAAVAGVGVDHQLLVGFAQSLNLESGSSSDSKVDSFNSSEIRHERGGNRAAVAVATHAPGWNSLNECLAGYVLQYAAGTGPVTKRGANNGALTKQIGESVASSALYSSYSDNGLFGFVVAGNAKEVGKAVEAGVKGLRSLNVSDADVARGKAGVYSWIAEYLESHGTLAFDLGEQAALLGKIYKKAEILAAIESVSTSDVQAAARKLASGKLAVGAVGNLSSVPYLCSLN |
| Aa-59830 | MGGHGHGPPYKVPDASIYKVADAPQLVEVERALARRGLKDPWLRNEVWRYNVKQFGTHRSRLLSFLFKGFPLGFAAFVATIGVEFALGVDYHGHGHGDGHGDDKHGHH |
| Aa-60163 | MATQFLNRIGQLGLGVAIVGGVVNSALYNVDGGHRAVIFDRFTGVKQQVSGEGTHFFVPWVQRPIIFDIRSQPRNVPVVTGSKDLQNVNITLRILFRPIPDQLPKIYTILGQDYDERVLPSITTEVLKAVVAQFDAGELITQREMVSQKVSDDLTERAAQFGVILDDISITHLTFGKEFTQAVEMKQVAQQEAEKARFMVEKAEQMKMAAIISAEXXAEAAALLAKSFGDSGDGLVELRRIEAAEDIAYQMSRSRGVAYLPAGQTTLLQLPQ |
| Aa-6266 | MVSTLNFVCLLLGVIIIISPTVKACCPTCSGCSSCGSKARTKEFLATATVYPPGEDCFPDEGAPQIAAPQSEASLNAPVEYPELEMRSPLAGALAATNDGGLERKASTNARGRTRWKQDAISTAAAAAQGKMDAKEAVMRNKRTSMLARGQRALQDKAMQMSSYMV |
| Aa-6370 | HTRNPTMNQLISLTIGALLLVALALRPAAAEIDPDDSCIPGTVGSIKSNLMRTLIICYVYGCPEELTSQIAANFNQFHRYDLRDEDDDDDSGNSTSSSSGGGGGGGNSTTTDDEKEVPTRILTKLAKLTSALEKDRTAALTACTDFVSLLNKLIVDILDINDKCFLEPQAKYPPDSADEGSIDCDEMTVYMKQLKKYYGGQCDSKGKNCPEKLKSAVSWFSRAYKQLGKSCYYDAYEYLDK |
| Aa-6647 | YPTCSIPEDAKKRARDILDGCKGHSVGSYTDSNGIEIIRKHVAQYIQDRDGGIPSDPNNIILSAGASGGIKVLMSLLRCPIDGKTPGVMIPIPQYPLYSATIAEFEMEQVGYYLDEANKWGLDIAELERSLKEAKKTCAPRILVVINPGNPTGQVLSRENIENIIKFAHKEKLVLFADEVYQDNVYEKGSKFHSFKKVLMEMGAPYNQMELCSFMSCSKGYMGECGIRGGYAEIVNMCPDVKAMLLKCISAQLCPTTIGQACMDVVVNPPKPGEPSYDQFMKEKNAVLASLKVRAEMVADTFNSIEGFSCNPVQGAMYAFPQIRLPEKAIEAAKKAGQAPDVFYAFQLLEQTGICIVPGSGFGQRPGTYHFRTTILPQPAKLKEMLDMFRAFHEKFLKQYK |
| Aa-6752 | FTAIWFRFFAERNFPIDRGDFVECSVRRPSSGDFSFCIFDSVTIFCEDPKQIXXXANVNLASVVLRNALRQKMGVRFSHDMIAQKIGKREVVGHGWNGLPVYADRVDYPMPAIRFKEVTPDVMALREKEKGDWKKLSMQEKKALYRASFCQTFSEIKYPTGEWKLSVGFGLIVLSMSLATMMLMKAFVYDDIPVTFDDEHQKAQLKRMLDLGVGNITGLSSKWDYENNKWK |
| Aa-6833 | MASLCGRLVLNAARRNVTYTPVRFCKMMNDPIEHATGLEKRELLARQAGDNDPFDMRVFKRGPGTKESPNMIPSAFESRLVGCVCEEDQTYIQWMWLHQGHPKRCECGHWFKLVEKAPV |
| Aa-7399 | MVNFTVDEIRSMMDRKRNIRNMSVIAHVDHGKSTLTDSLVSKAGIIAGAKAGETRFTDTRKDEQERCITIKSTAISMYFELDDQDLVFITNPDQRDKDCKGFLINLIDSPGHVDFSSEVTAALRVTDGALVVVDCVSGVCVQTETVLRQAIAERIKPVLFMNKMDRALLELQLDAEDLYQTFQRIVENVNVIIATYNDDGGPMGEVRVDPSKGSVGFGSGLHGWAFTLKQFAEMYAAMFKIDVVKLMNRLWGENFFNPKTKKWAKVKDDDNKRSFVMYVLDPIYKVFDAIMNYKTDEIPKLLEKLKVTLKHEDKDKDGKNLLKVVMRTWLPAGEALLQMIAIHLPSPVVAQKYRMEMLYEGPHDDEAAVAVKNCDPEGPLMMYVSKMVPTSDKGRFYAFGRVFAGKVATGQKCRIMGPNYTPGKKEDLYEKAIQRTILMMGRYVEAIEDVPCGNICGLVGVDQFLVKTGTISTFKDAHNMKVMKFSVSPVVRVAVEPKNPADLPKLVEGLKRLAKSDPMVQCIIEESGEHIIAGAGELHLEICLKDLEEDHACIPLKKSDPVVSYRETVSDESDQMCLSKSPNKHNRLFMKAVPMPDGLAEDIDNGDVNSRDDFKVRARYLAEKYDYDVTEARKIWCFGPDGTGPNIVVDCTKGVQYLNEIKDSVVAGFQWASKEGVLAEENMRAVRFNIYDVTLHADAIHRGGGQIIPTARRVLYASYITAAPRIMEPVYLCEIQCPEVAVGGIYGVLNRRRGHVFEEAQVAGTPMFVVKAYLPVNESFGFTADLRSNTGGQAFPQCVFDHWQILPGDPAXXXXXXPYAVVQDIRKRKGLKEGLPDLSTK |
| Aa-74093 | MNPEFQEIVSPRQLQRSPLRDASAHYGPQNYSQTSRIRPPGTIGSTSRIPPPMGQRQVAQTPSVRPPSMIRPPGGGQFQTPQTVQRPSQIRLPSGLKAPGFFRPSVQSTPLGPSTSSQKVIPLPGSANALAQAPKPSRIPAPPIGSSGIPTPSVSRVPSTSLLPKPSFTRSQSQSRIIPPSRTVLPRTSTPQRTSIPAQGRRPGSAPSTAGPSRVRPTTAPTTPRVVREAVPSTPVRRPKVQLPRTAAKKRIATEDANAEVYSIAGREVEFVDYVPSPEDEPAVRPSRRGPAARPGAVRIAKGIVTKPSGRGQIAAEPKTPVAAGRARINLPRTAAKQRITSEDTEGEVFNIGGRVVEFVDYVPTPEEQPPPEPVRRPVVRRGGARIKMPEKPGDKPDGAPPVVEAKQAVQKSRKIHEFPDEPSVAAAKEFASQIDADIQRFRQMGADLTDLKQRKSAVLSKIKSMYDQDKAEDLDVPAIPDVEGFRAKYGVTQSPKMRQLLEVTTDWNRTILDDQEFAESLAQVRPFEPQQKPKLTTVLQSLVESPDRPDQVVQRRSEMELARQERIDNYRQRIEAAKEYRRILEERRKNLPSRRPYSKQEREDIRRRYQEAQEWTYTAPTEEELDNAMRLAEMMELEGAGVSVVADPKETVETLFPEAAPSVEISGVVGDVPIPRDIPAEEINNVNDLDRSTAEVALKASLVPVPTEGVTKKVLEAGLQEMTTAKIEPTPQKLDPMYEIDLKLGQPRRKNYIQLQGSSYPSWFPGFYSIFPGAPGLIKAMKSPSATLLTAFRQMHSSAYI |
| Aa-7502 | MGKEKTHINIVVIGHVDSGKSTTTGHLIYKCGGIDKRTIEKFEKEAQEMGKGSFKYAWVLDKLKAERERGITIDIALWKFETSKYYVTIIDAPGHRDFIKNMITGTSQADCAVLIVAAGTGEFEAGISKNGQTREHALLAFTLGVKQLIVGVNKMDSTEPPYSESRFEEIKKEVSSYIKKIGYNPAAVAFVPISGWHGDNMLEVSTKMPWFKGWNVERKEGKADGKCLIEALDAILPPSRPTDKALRLPLQDVYKIGGIGTVPVGRVETGVLKPGTVVVFAPVNLTTEVKSVEMHHEALQEAVPGDNVGFNVKNVSVKELRRGYVAGDTKNNPPKGAADFTAQVIVLNHPGQISNGYTPVLDCHTAHIACKFAEIKEKVDRRSGKSTEENPKSIKSGDAAIVNLVPSKPLCVESFQEFPPLGRFAVRDMRQTVAVGVIKSVNFKDASGGKGYQGAEKAQKGKK |
| Aa-7922 | ERISMFKAVSDFEELTEKLSQGSKVAIIGGGFLGSELSCALAKCSEIRNKNFEVYQLFHEEGNMGKILPEYLSQWTTERVREEGVKVWPKIQVKAAEVQDKKLKLTLTDDSVLVVDHAIVAVGSEPNTDLAKTSNLETDPAMGGFVVDAELRARSHLYVAGDAACFFDPKLGRRRVEHHDHAVVSGRLAGENMVGLNKPYTHQSMFWSDLGPKIGYEAIGIIDSALPTVAVFAKANPPQTIPDTSEKLQAANAATVTTTASNGGESSILGNPNVVKAEQKPPAPTEEDKDDFNKGVIFYLKDEKVVGVLLWNVFNRIGTARRIVAQHTRYDDLNEVAKLFNLHERPEEPEAEEEEANK |
| Aa-7971 | MCDEEVAALVVDNGSGMCKAGFAGDDAPRAVFPSIVGRPRHQGVMVGMGQKDSYVGDEAQSKRGILTLKYPIEHGIVTNWDDMEKIWHHTFYNELRVAPEEHPVLLTEAPLNPKANREKMTQIMFETFNTPAMYVAIQAVLSLYASGRTTGIVLDSGDGVSHTVPIYEGYALPHAILRLDLAGRDLTDYLMKILTERGYSFTTTAEREIVRDIKEKLCYVALDFEQEMATAASSSSLEKSYELPDGQVITIGNERFRCPEALFQPSFLGMEACGIHETTYNSIMKCDVDIRKDLYANTVLSGGTTMYPGIADRMQKEITALAPSTMKIKIIAPPERKYSVWIGGSILASLSTFQQMWISKQEYDESGPSIVHRKCF |
| Aa-8460 | PTDDFHNLHPLLVRNSRWLSEDHMFIYQHQCQPEPAPPTLTDWGAFWLVGISLLLCCCFQSFQGSATQDQGRKMNPESISHLEHICSLDIDSETAFVRLSGIICTIGPASVAPEMLEKMMATGMNIARLNFSHGSHEYHANTIKNIREAVDNYSKKQGKPFPLAIALDTKGPEIRTGLIEGSGTGEVELKKGEQIQLTTDKDHLEKGSKEKIYVDYVNIVKVVKKGDHVFVDDGLISLVVDSISGDTLTCTVENGGMLGSRKGVNLPGVPVDLPAVSEKDKSDLQFGVEQGVDVIFASFIRNAAALKEIRSILGDKGKNIKIISKIENQQGMQNLDAIIAASDGIMVARGDLGIEIPAEKVFLAQKSMIARCNRAGKPVICATQMLESMIKKPRPTRAEISDVANAIIDGADCVMLSGETAKGEYPLECVLTMAKTCKEAEAALWHRNLFNDLVNTTPTPLDTASSIAIAASEAASKSRAAAVIVITTSGRSAHLISKYRPRCPIIAVTRFAQTARQCHLYRGILPVVYEQQALEDWLKDVDARVQYGMDFGKERGFLKPGNPVVVVTGWKQGSGFTNTIRIVNVE |
| Aa-8918 | GLQNSTSPLMEQLNFFHDHTLLILIMITIMIAYIMFMLFFNKFTNRYLLHGQTIEIIWTILPTIILMFIAFPSLRLLYLMDEINSPLITLKVIGHQWYWSYEYSNFLNLEFDSYMIPTNELDINGFRLLDVDNRVILPMNNQIRILVTATDVIHSWTVPSMGMKIDATPGRLNQTNFLMNQPGLFYGQCSEICGANHSFMPIVIESIPMNYFIKWISSQMN |
| Aa-9290 | PGGGEDAAFGLKPSGGDVKFEKKVGDAVAADEVVMEIETDKTTVGVPAPAHGIIEEIYVADGDTVKAGQQLFKLKITGEAPAAGAPKAEAPAPAAAAPPPPPPPPPVAAAAAAAPPPPPPAAAAAPPPPPPRPTGPITKMPVAAMRHAQAIDAATVKLPPADYTKEITGTRTEQRVKMTRMRLKIASRLKEAQNTNAMLTTFNEIDMSFIMDFRKQHLEAFQKKYGMKLGFMSAFCKAAAYALQDQPVVNAVIGENEIIYRDYVDISVAVASPKGLVVPVLRNVEGMNFADIELAIAGLADKAKKGTLAVEDMDGGTFTISNGGVFGSLLGTPIINPPQSAILGMHGIFERPIAVKGQVVVRPMMYVALTYDHRLIDGREAVTFLRKVKAAVEDPRIILAGL |
| Aa-9760 | MTATCSSMAIGTYWPCFSNSVSRTPRFNSCWVAASSSEPNWAKAATSRYWANSSFMEPATCFMALVPIGRGQRELIIGDSQTGKTALAIDTIINQQRFNNGTDESKKLYCIYVAIGQKRSTVAQIVKRLTDAGAMNYTIIVSATASDAAPLQYLAPYSGCAMGEYFRDNGKHALIIYDDLSNQAVAYRQMSLLLRRPPGREAYPGDVFYLHSRLLERAAKMNPTLGGGSLTALPVIETQAGDVSAYIPTNVISITDGQIFLETELFYKGIRPAINVGLSVSRVGSAAQTKAMKQVAGSMKLELAQYREVAAFAQFGSDLDAATQQLLNRGVRLTELLKQGQYVPMAIEEQVAVIYCGVRGYLDKMDPSKITAFEREFLAHVKTNEKALLSQIATDGKISDETEAKLKNVVTSFMSTFSG |
