# Supplementary material for: Identification and Characterization of Seminal Fluid Proteins in the Asian Tiger Mosquito, Aedes albopictus
Source: PLoS Negl Trop Dis. 2014 Jun 19;8(6):e2946. doi: 10.1371/journal.pntd.0002946 (PMC4063707; doi:10.1371/journal.pntd.0002946)
Supplement: Table S5 — Amino acid sequences of Ae. albopictus proteins identified in the labeled virgin females. (DOCX) [file pntd.0002946.s005.docx]

| **Protein identified in labeled females** | **Amino acid sequence** |
| --- | --- |
| Aa-3848 | STGGVKKPHRYRPGTVALREIRRYQKSTELLIRKLPXXRLVREIAQDFKTDXRFQSAAIGALQEASEAYLVGLFEDTNLCAIHAKRVTIMPKDIQLATPYPRRTCLSSRHLLLLILL |
| Aa-15006 | MPEAAADVETFAFQAEIAQLMSLIINTFYSNKEIFLRELISNSSDALDKIRYESLTDPSKLDSGKELYIKLIPNKEAGTLTIIDTGIGMTKADLVNNLGTIAKSGTKAFMEALQAGADISMIGQFGVGFYSSYLVADKVIVTSKSNDDEQYVWESSAGGSFTVRQDTGEPLGRGTKIEDVEDDEEKKDKKKKTVKVKYTEDEELNKTKPIWTRNADDISQEEYGEFYKSLTNDWEDHLAVKHFSVKVNWTSVPCSSCPVVCRSISLRTRRRRTTSSCTSVASSSWTTARN |
| Aa-35743 | MTGRGKGGKGLGKGGAKRHRKVLRDNIQGITKPAIRRLARRGGVKRISGLIYEETRGVLKVFLENVIRDAVTYTEHAKRKTVTAMDVVYALKRQGRTLYGFGG |
| Aa-38093 | MAGGKAGKDSGKAKAKAVSRSARAGLQFPVGRIHRHLKNRTTSHGRVGATAAVYSAAILEYLTAEVLELAGNASKDLKVKRITPRHLQLAIRGDEELDSLIKATIAGGGVIPHIHKSLIGKKGGPE |
| Aa-63600 | RIRQQELNPVFHWQDKTEDDKVLSKIKIKQALKSGVLNLSGQGLATVPEKVWNLSDSEDCDKEVRYDLDRSNEEESWWNQKTLTNLDLSSNALTSISENVKNLGDLTVLNLQDNALISLPDGIGCLSKLTKLNISRNKLIELPESFFELKELKVLNLSHNDFAEIHSNVSDLIMLEVLDISFNSLNSLPGGIGFLVRLQQLTLNNNRLTELPNDIVNLRNLHKMDLAKNDLKQLPPVMGELRKLECLYVQHNDVGELPDFTGCDALKEIHISNNFIRSIPADFCENLPQLKVLDLRDNKIEKLPDEISMLASLTRLDLSNNSISSLPSCLSTLAHLVSLQVEGNPIRSIRRDIIQCGTQRILKTLRERDGPGRXXXXXPFEESTFPDVYQMKKGRSMIVSNKNLIDIPEQVFLDASEASVYNVDISKNKLGEVPSGITNLADQLTELNISFNLLKTIPMFFSRFERISYLNVSNNLLSDLPEVVGLLVTLRELNVANNQLKRIPPCVYELKGLEILLARDNKIEEIDATR |
| Aa-136683 | MAPKTSGKAAKKSGKAQKNIVKGDKKKKKQRRKESYAIYIYKVLKQVHPDTGVSSKAMSIMNSFVNDIFERIAAEASRLAHYNKRSTITSREIQTAVRLLLPGELAKHAVSEGTKAVTKYTSSK |
