# Supplementary material for: Identification and Characterization of Seminal Fluid Proteins in the Asian Tiger Mosquito, Aedes albopictus
Source: PLoS Negl Trop Dis. 2014 Jun 19;8(6):e2946. doi: 10.1371/journal.pntd.0002946 (PMC4063707; doi:10.1371/journal.pntd.0002946)
Supplement: Text S1 — Description of methods used for transcriptome sequencing, assembly, and generation of Ae. albopictus reproductive tract predicted protein database. (DOCX) [file pntd.0002946.s009.docx]

**Transcriptome sequencing: isolation of mRNA and development of cDNA libraries***Ae. albopictus* mosquitoes (New Jersey strain; see [1]) were reared at a standard density of 200 larvae per liter, and fed with a standard yeast regimen (a 1:1 mixture of Brewer’s yeast and lactalbumin). Mosquitoes were maintained in an incubator at 27 °C on a 12:12 light:dark cycle. To obtain RNA, the reproductive tract (not including the ovaries) was dissected out from 805 virgin 4-7 day old females, and the accessory glands and seminal vesicles were dissected from 720 sexually mature virgin 1-4 day old males. All dissections were performed on ice in 0.7% sodium chloride made with DEPC-treated water. Tissues were placed into Trizol (Invitrogen, Carlsbad, CA), ground with a pestle, and stored at -80 degrees.

RNA extraction and cDNA library synthesis were conducted at the W.M. Keck Center for Comparative and Functional Genomics (Roy J. Carver Biotechnology Center, University of Illinois at Urbana-Champaign). mRNA was isolated from 10 µg of total RNA with the Oligotex kit (Qiagen, Valencia, CA). The mRNA-enriched fraction was then converted to 454 barcoded cDNA libraries. These libraries were generated, normalized, quantified, and average fragment sizes determined as described previously [2]. The libraries were diluted to 1x10^8^molecules/µl and pooled in equimolar concentration.

cDNA library sequencing was conducted at the Cornell University Genomics Facility. The cDNA libraries were sequenced using standard Roche/454 shotgun library preparation kits, Titanium sequencing reagents, and data analysis software (454 Life Sciences, Branford, CT).

**Transcriptome assembly and generation of predicted protein database**Assembly of the reads was performed as described previously [3] by using 12 iterations of blastn [4] and CAP3 [5] rounds in a parallel computer array, to produce a final file of contigs. A subset of these raw data is available at the Sequence Read Archives of the National Center for Biotechnology Information under BioProject ID PRJNA223166 and accession SAMN02378346. The blastn program was run at decreasing word sizes (from 300 to 60) and its output was used to feed the CAP3 assembler in a non-redundant manner (no sequence was used more than once per cycle). Coding sequences (CDS) were extracted from the contigs based on their matches to a subset of proteins from the non-redundant (NR) protein database of the National Center for Biotechnology information (NCBI), and from the Swissprot database. All of the coding sequences coding for a secreted protein (indicated by a signal) were extracted. Corrections were made for frame shift when these appeared, a common occurrence with pyrosequencing. Additionally, the larger open reading frame (ORF) of each contig was extracted, and peptides longer than 30 amino acids (aa) starting with a methionine were sent to the signalP program version 3.0 (Nielsen et al. 1999) running locally. If one or more signal peptide were found, the most aminoterminal would be used to select for a putative secreted protein. These two sets of coding sequences were compared to remove redundancy. Transmembrane domains of proteins were identified with the tool TMHMM [6], mucin-type O-galactosylation was identified with the program NetOglyc [7], peptide furin cleavage sites with the ProP server [8], all running locally on the NIH Biowulf cluster. The proteins predicted from the contigs were used subsequently for the mass-spectrometry based identification of proteins, as described further in the main text. The sequences that were believed to be at or near full length (6,887 total sequences) are available through the Transcriptome Shotgun Assembly project, which has been deposited at DDBJ/EMBL/GenBank under the accession GAPW00000000. The version described in this paper is the first version, GAPW01000000.

**References:**

1. Helinski MEH, Deewatthanawong P, Sirot LK, Wolfner MF, Harrington LC (2012) Duration and dose-dependency of female sexual receptivity responses to seminal fluid proteins in *Aedes albopictus* and *Ae. aegypti* mosquitoes. J Insect Physiol 58: 1307–1313. doi:10.1016/j.jinsphys.2012.07.003.

2. Lambert JD, Chan XY, Spiecker B, Sweet HC (2010) Characterizing the embryonic transcriptome of the snail *Ilyanassa*. Integr Comp Biol 50: 768–777. doi:10.1093/icb/icq121.

3. Karim S, Singh P, Ribeiro JMC (2011) A deep insight into the sialotranscriptome of the Gulf Coast tick, *Amblyomma maculatum*. PLoS ONE 6: e28525. doi:10.1371/journal.pone.0028525.

4. Altschul SF, Madden TL, Schäffer AA, Zhang J, Zhang Z, et al. (1997) Gapped BLAST and PSI-BLAST: a new generation of protein database search programs. Nucleic Acids Res 25: 3389–3402.

5. Huang X, Madan A (1999) CAP3: A DNA sequence assembly program. Genome Res 9: 868–877.

6. Sonnhammer EL, von Heijne G, Krogh A (1998) A hidden Markov model for predicting transmembrane helices in protein sequences. Proc Int Conf Intell Syst Mol Biol ISMB Int Conf Intell Syst Mol Biol 6: 175–182.

7. Julenius K, Mølgaard A, Gupta R, Brunak S (2005) Prediction, conservation analysis, and structural characterization of mammalian mucin-type O-glycosylation sites. Glycobiology 15: 153–164. doi:10.1093/glycob/cwh151.

8. Duckert P, Brunak S, Blom N (2004) Prediction of proprotein convertase cleavage sites. Protein Eng Des Sel 17: 107–112. doi:10.1093/protein/gzh013.
