# Supplementary material for: Identification and Characterization of Seminal Fluid Proteins in the Asian Tiger Mosquito, Aedes albopictus
Source: PLoS Negl Trop Dis. 2014 Jun 19;8(6):e2946. doi: 10.1371/journal.pntd.0002946 (PMC4063707; doi:10.1371/journal.pntd.0002946)
Supplement: Text S2 — Identification of additional putative Ae. albopictus seminal fluid proteins by searching mass spectra against the Ae. aegypti genome. (DOCX) [file pntd.0002946.s010.docx]

**Identification of additional putative *Ae. albopictus* seminal fluid proteins by searching mass spectra against the *Ae. aegypti* genome**
The *Ae. aegypti* genome sequence [1,2] is more complete and more thoroughly annotated than our *Ae. albopictus* transcriptome-based database. Therefore, the *Ae. aegypti* genome sequence provides a resource with which to potentially identify mass spectrometry hits that we could not identify by comparisons to our *Ae. albopictus* database. Thus, in order to identify additional putative *Ae.albopictus* seminal fluid proteins (Sfps), the *Ae. albopictus* mass spectra files from this study were searched against the *Ae. aegypti* genome.

The search was conducted by the Ohio State University Mass Spectrometry and Proteomics Facility.

All MS and MS/MS spectra were searched against the *Ae. aegypti* genome (Vectorbase AaegL1.4 and AaegL2.2; [1]) using Mascot Daemon (version 2.3.02, Matrix Science, Boston, MA) with one missed trypsin cleavage allowed. Peptide mass tolerance was set to 5 ppm and MS/MS mass tolerance was set to 0.8 Da. Fixed and variable modification settings were as described in the main text. Putative Sfps were identified using the same criteria as described in the methods section (see Methods: Protein identification by nanoLC-MS/MS analyses, verification and selection).

We identified 125 putative Sfps from our search against the *Ae. aegypti* database. Of these, 98 had high sequence similarity (E-value < 8.0*E^-8^) with *Ae. albopictus* transferred proteins that we had already identified through our original search, and are assumed to be the same as those reported in the main paper. An additional five proteins shared high sequence similarity with *Ae. albopictus* proteins that showed no evidence for transfer to females in our original proteomics data. Therefore, we assume that these five are unlikely to be transferred proteins in *Ae. albopictus.* We consider the remaining 22 proteins identified through the search against the *Ae. aegypti* database to be newly-identified putative Sfps. Of these 22 proteins, 14 showed high sequence similarity (E-value < 8.0*E^-8^) to a predicted protein in the *Ae. albopictus* reproductive tract transcriptome database reported in the main paper. It is possible that the reason we identified these proteins as high confidence hits in our *Ae. aegypti* search but not in our *Ae. albopictus* search is because the corresponding transcripts in our *Ae. albopictus* database are incomplete. The remaining eight proteins (AAEL000274, AAEL000619, AAEL004389, AAEL005964, AAEL006698, AAEL011704, AAEL012665, AAEL017994) did not show high sequence similarity to predicted proteins in the *Ae. albopictus* reproductive tract transcriptome database. To determine whether those eight proteins have a putative ortholog in *Ae. albopictus*, their sequences were compared using BLASTp to sequences in two additional databases: a reference predicted protein set for *Ae. albopictus* that was generated from combining several transcriptomes available for the species (see Methods: Determining sequence similarity with proteins from other species), and the primary assembly of the *Ae. albopictus* reproductive tract transcriptome database. Orthology was determined as described in the main paper (see Methods: Determining sequence similarity with proteins from other species). Four of the eight proteins (AAEL000274, AAEL004389, AAEL006698, AAEL011704) have a putative ortholog in the reference *Ae. albopictus* predicted protein set, and two of those four proteins (AAEL004389, AAEL006698) also have a putative ortholog in the primary assembly of the *Ae. albopictus* reproductive tract transcriptome. The remaining four proteins (AAEL000619, AAEL005964, AAEL012665, AAEL017994) did not have a putative ortholog in any of these *Ae. albopictus* databases. This likely reflects their ortholog not being included in the limited databases that were available to us to screen. However, there is a possibility that they do not have a putative ortholog in *Ae. albopictus*, which could be the case if two or more peptides from the *Ae. aegypti* protein matched the mass spectra of the *Ae. albopictus* protein, but the remaining portion of these proteins did not share sequence similarity.

The results of this analysis are presented in the Supplemental Table below. In cases where an *Ae. aegypti* protein showed high sequence similarity to more than one protein from the *Ae. albopictus* reproductive transcriptome database, we report the *Ae. albopictus* protein that has the highest number of peptide hits in the female samples in our original proteomics data, and secondarily the *Ae. albopictus* protein of greatest length.

**Supplemental Table. Additional putative *Ae. albopictus* seminal fluid proteins, identified by searching mass spectra against the *Ae. aegypti* genome.**

| ***Ae. aegypti* protein** | **Putative protein class^a^** | **Male Tissue^b^** | ***Ae. albopictus* protein having high sequence similarity to  *Ae. aegypti* protein^c^** | **Coverage (%)** | **E-value** | **Identity (%)** | **Putative ortholog to *Ae. aegypti* protein in primary assembly?^d^** | **Coverage (%)** | **E-value** | **Identity (%)** |
| --- | --- | --- | --- | --- | --- | --- | --- | --- | --- | --- |
| AAEL000274 | Copper-Zinc(Cu-Zn) Superoxide Dismutase | T | Aalb_OocyteEmbryoPharatelarvaeAssembly_023153.1 | 100 | 4.00E-102 | 97 | - | - | - | - |
| AAEL000301 | Heat shock protein | T | Aa-48769 | 81 | 0 | 99 | N/A | N/A | N/A | N/A |
| AAEL000619 | None | - | - | - | - | - | - | - | - | - |
| AAEL001951 | Actin | - | Aa-126542 | 56 | 5.00E-146 | 99 | N/A | N/A | N/A | N/A |
| AAEL002793 | None | - | Aa-19892 | 100 | 0 | 87 | N/A | N/A | N/A | N/A |
| AAEL004389 | Mannosidase alpha class 2a | - | Aalb_OocyteEmbryoPharatelarvaeAssembly_086539.1 | 99 | 0 | 95 | sirot_asb-75251 | 2 | 7.00E-11 | 100 |
| AAEL004988 | Kinase (phosphoglycerate kinase) | - | Aa-36686 | 100 | 0 | 99 | N/A | N/A | N/A | N/A |
| AAEL005964 | Actin | T | - | - | - | - | - | - | - | - |
| AAEL006091 | Rab6 | - | Aa-29791 | 87 | 1.00E-138 | 100 | N/A | N/A | N/A | N/A |
| AAEL006698 | 60S ribosomal protein L31 | T | Aalb_OocyteEmbryoPharatelarvaeAssembly_145945.1 | 100 | 2.00E-86 | 100 | sirot_asb-84606 | 21 | 2.00E-09 | 96 |
| AAEL008489 | Calcyphosine/tpp | - | Aa-46286 | 100 | 5.00E-159 | 98 | N/A | N/A | N/A | N/A |
| AAEL011291 | Protease m1 zinc metalloprotease | - | Aa-5383 | 55 | 0 | 96 | N/A | N/A | N/A | N/A |
| AAEL011704 | Heat shock protein | T | Aalb_OocyteEmbryoPharatelarvaeAssembly_121969.1 | 100 | 0 | 99 | - | - | - | - |
| AAEL012010 | None | T | Aa-48005 | 100 | 5.00E-107 | 96 | N/A | N/A | N/A | N/A |
| AAEL012665 | Glucose-6-phosphate isomerase | - | - | - | - | - | - | - | - | - |
| AAEL014842 | Multiple inositol polyphosphate phosphatase | - | Aa-29084 | 69 | 0 | 89 | N/A | N/A | N/A | N/A |
| AAEL015110 | Dipeptidyl-peptidase | - | Aa-47532 | 43 | 0 | 88 | N/A | N/A | N/A | N/A |
| AAEL016968 | None | - | Aa-28005 | 60 | 0 | 99 | N/A | N/A | N/A | N/A |
| AAEL017499 | None | - | Aa-29771 | 84 | 0 | 94 | N/A | N/A | N/A | N/A |
| AAEL017973 | Heat shock protein HSP70 | T | Aa-14313 | 100 | 0 | 95 | N/A | N/A | N/A | N/A |
| AAEL017994 | None | - | - | - | - | - | - | - | - | - |
| AAEL018249 | None | - | Aa-3733 | 58 | 0 | 92 | N/A | N/A | N/A | N/A |

^a^ Putative protein class information from Vectorbase.org.

^b^ Male tissue in which proteins from *Ae. aegypti* genome were identified with high confidence (see Text S2 and Methods). SV = seminal vesicles; T = testes; - = neither seminal vesicles nor testes (putative protein from accessory glands).

^c^ *Ae. albopictus* sequence from the reproductive tract transcriptome database (Aa- numbers; sequences available in protein sequence list below), or from the Oocyte Embryo Pharate larvae assembly within the *Ae. albopictus* reference predicted protein database (Aalb_ numbers; from [3]). - = no putative ortholog was identified.

^d^ This information was only assessed for *Ae. aegypti* proteins that do not have high sequence similarity with proteins in the *Ae. albopictus* reproductive tract transcriptome database. - = no putative ortholog in primary assembly. Full sequences of the two putative orthologs in the primary assembly are available in the protein sequence list below.


**Amino acid sequences for *Ae. albopictus* proteins listed in Supplemental Table:**

| ***Ae. albopictus* protein** | **Amino acid sequence** |
| --- | --- |
| Aa-3733 | NCGPRWRMERIGCFLDDNSSFVLDPNREFVTVSTPGVILNGTYFLEFEYYGELREDNAGFYRSSYVDDEGKTRWLATTQF  SSTDARHAFPCYDEPGIRAPIGLTVIHGSNYSVLSNNLPQDVGQGPIAGYTRTVFDDTPVMQPYLLGVIVSDFVAVSAVQ  YPRQGVFARYNAIRNGEGDFILEAGYKILKVLESYLETDFALPKIYQVAVPDFAAGAMENYGLVTYKEEKFFYDSRSSPM  RQKHEIATVVGHEYGHQFFGNMVSPAWWSYLWMKEGFARYFEYLASDMAFPELRIRETYSIQKMQNAFDLDSLGSSRPMT  FYVNTQSEIANVFDNIAYDKGGSMMRMFQHAFGPETFRRALINYLRAKAFQGAYPEDFANAIQQALNNATEVTVPSSITA  LDILKSWTEKSGYPVITVSRGTDLRVSLIQERYMLKIMDTTEASWIIPYNFATTKNPNFNTTTDTRWLLTNSTILAAEGW  SATDWIIFNKQQTGYYRVNYDERLWNLIIEQLMRDPTAIHNMNRAQLVDDVLNFARSGRLGYDTALRLIAYLVRERDYVP  WYAANSNLLVLTRLFAGSPKIEYLKNTC |
| Aa-5383 | GFDEARHPGGVRREIVVDLFDVLEEEALGQVEADVALGDTAEHDGRVLLAHSRSAVQGPQQEPFDQPDFTGAGQCSDDHD  LLRADVALEVALPQEVPLRFLAVAHGVAVHHGAEVPRDRVGQLRILDGHPLREQGYRSFNTVSNVELTRPGGDLNQLFGS  VQSLTDGSIFAEDLQLLVDGFNDELDVASEHSVDRGALQEAVDRVEVREGRLPGDVFHLLAKVVDHGEGDAVLLFSGLYK  VRYDKTNYKLLIKQLNSEQYSTISLANRAQLIDDAMDLAWTGEQQYGIAFAMINYLRQEVEYIPWKSALSNLNAINRLLK  RTPIYGVFRSYIQFIVEPIYEKLQIFSEDRAVSQRLDATKQLVQIAAWACKFDVGDCVERSVALFAKWMAVQDPELSNPV  PRDLRSVVYCNAMRNGKESEWNFLWQRYLKSNVGSEKVMIIGALSCTREVWLVERFLLWSLNSTSGVRKQDTTIVFGGVA  KSDVGFHLAKSFFLENVEEIYNYLSPDTSRVSRFIKPLAEQMSSMKELQELKDLIESKRTVFEKATQGVKQALETVEINL  QWKSYGYTQMTRFLPLLSYRSGNLDVMELLD |
| Aa-14313 | MSAIGIDLGTTYSCVGVFQHGKVEIIANDQGNRTTPSYVAFSDTERLIGDAAKNQVALNPQNTVFDAKRLIGRRFDDPKI  QADIKHWPFKVFNDGGKPKIEVEFKGERKRFAPEEISSMVLVKMRETAEAYLGKSVKNAVITVPAYFNDSQRQATKDAGA  IAGLNVMRIINEPTAAALAYGLDKNLKGERNVLIFDLGGGTFDVSILTIDEGSLFEVRATAGDTHLGGEDFDNRLVSHFV  EEFKRKFKKDISTNPRALRRLRTACERAKRTLSSSTEATIEIDALIEGIDYYTKISRARFEELCSDLFRSTLQPVEKALS  DAKMDKSSIHDIVLVGGSTRIPKVQNLLQNFFCGKSLNLSINPDEAVAYGAAVQAAILSGDKDEKIQDVLLVDVAPLSLG  IETAGGVMTKLIKRNSRIPCKQTQIFSTYADNQPGVSIQVFEGERAMTRDNNRLGQFDLSGIPPAPRGVPQIEVTFDMDA  NGILNVSAKEKSTGKEKNITIKNDKGRLSQADIDRMVNEAEKFREEDEKQRERISARNQLEGYCFQLKQSVESAGGKLSE  SDKNTMKEKCDETLRWLDGNTMAEKDEFEHKMQELSRVCSPIMTRLHQSGAEPGARDSSCGGQQAGGCGGQSQTGPTIEE  VD |
| Aa-19892 | SGSICTGCRDNWTMNKKAKCIANGSNNCDESEYYENGHCHSCHSTCETCTGPTEHECLTCASPLLLQNQRCVNECDEGYY  MEVGVCAKCLHTCTQCVSRMNCTSCQKGLQLQSGECRTTCADGYYSDRGTCAKCYLSCNTCSGPRRDQCVTCPEGWQLAG  GECHPECPEGFFKTKFGCQKCHHYCKTCNAAGPLACTSCPSHFMLDGGLCIECLGSQFYDPPTQTCKTCHDSCRSCSGPG  QYSCVTCAFPLHLDRLNNQCVPCXXXXXXXXXXXXXXXXXXXGGCINSSPAGKRRIGAEQQLLQFGEEAANSAGDSNAAE  ASDSSLRQFPRGGDTPSAVTVVAVSGCLLIVTLFAIIFTVLQKRNRTVYTGVRYNKLSSGGGSGAGGTRTTRISIPADGD  DLLEPIVNNSEDDEDDDDDEDEEDENNTGGRGDHVYHDDDHELHDGAAAVVATNKSSSSGHSSRQSRFVATGHHGASRRG  TRPQPDSIRFART |
| Aa-28005 | LVGVIKRLSEFAVEYRDLPTLGFTHLQPAQLTTVGKRCTLWVQDLLMDERALRNCRDNLCFRGVKGTTGTQASFLQLFAG  DGDKVRQLDQKVTNLAGFEKYYAVTGQTYTRKVDLEIVSALSSLGATVHKMCSDLRLLASRKEVEEPFEKTQIGSSAMAY  KRNPMRSERCCALARHLITLHASAANTLAVQWLERTLDDSANRRLTLSEAFLSADACLLTLLNISQGLVVYPKVIERNIA  QELPFMSTENVIMAMVQAGGDRQICHEKIRVLSHEAGAQVKQHGKDNDLVERIRADAYFAPILGDLDKILDPKTFTGRAA  DQVLEFVREEVDPVVARYGSNVETKSIGLASV |
| Aa-29084 | NYTPRLLTDQGWSDLKLLARREKDRFNEVFNWPYDKQRYLFRHTKSQRTEASFKAFVEGLFGDGAYNFINAEPEPTDDTL  LKPYDFCPAYDANKDKNKQPDSELNKFLRSRIYLQTLSDISTRLGFRYNLSNDQIEAMWDICRYEQAWHLQQYSPWCSVF  TKNQVNILEYKEDLRYYYQNSYGYEKSADLACYAVNDMVKNLGRSDGQQVIAYFTHEAEIQIFLTALGAKKDRDSLRAEN  YYAMQNRNFRSSELTPFAANLAAVRYQCADPVEPVKVIFFLNEKALMFDWCRVGLCDWSEVVRRYERYTKADCAKMYCGG  SGASSWTLHWTTMMGIVLTIAVGLLQR |
| Aa-29771 | QSCFCPIRKGFHIVREIGQVRSVGGTKPLLLRRIMVHKSRKLSAVLVIAVVVSGFVLVANGFTLKESSPAAPGAIIEEDE  SFLQNPRYESNDELQDLLARLQKDHPTLVKVHSIGSSLENRPLLVVEIRPNIDRPRPLLMPMFKYVANMHGDETVGRELL  IYLAQYLVNNYRQDPEVGALVNSTAIYLMPSMNPDGFHRSKEGSCESPPNYMGRYNAAGVDLNRDFPDRFDNDRVRHIRR  NRQQPETAAVMNWILSNPFVLSANLHGGAVVASYPYDNSIFHHECCEDSPTPDNHFFKYASLVYAQNHPVMKNGHDCNET  FQDGITNGAYWYELNGGMQDFNYVFSNCFEITLELSCCKYPKASELPKEWHKNKRSLIEYMKLTHVGVRGLVTDSNGYPI  QDADVIVDGIKQNIRTTKRGEYWRLLVPGNYKLRVEAVGFYPSQEVPITIAAEQPLRVNFSLKSYDADEGDAAGKVSSAI  AVADKTKHPRVVRQQPDEYGFLMKPKFEHHNFTAMESMIHDLAGNYPSITRLYSIGKSVQQRDLWVMEITRNPGKHIPGK  PEVKYIANMHGNEVVGREMLLLLAKYLCENYNRTERVTKLVNNTRLHILFSMNPDGYEISEMEDKDNLKGRSNANNVDLN  RNFPDQFGRNNYNMKQEPETHAVMNWSLSIPFVLSANLHGGALVANYPFDDSPKDFAYSNGYGDPRTVYNPTEEDEMFKY  LAHTYANAHTTMHLGKPCPTYIKESFKDGITNGAAWYSVTGGMQDWSYIVGGAYELTLEVGCDKFPKAEELPSFWQQNRE  ALLRYVEQAQHGIYGTVKSTIGHPIGHATIQVNNIQHVTFSTAEGDFYRILLPGLYNVTAEAAGYEPQTVQVRIPPEATS  AVVVDFLLMRDDPQHWSSAYDYRTLENVVRTRYHSDSELRVIMSEFENKNYKTASLEFGDNEVAMAFPSVKMTDNIGTPE  ETKLHILV |
| Aa-29791 | MSSSGDFGNPLRKFKLVFLGEQSVGKTSLITRFMYDSFDNTYQATIGIDFLSKTMYLEDRTVRLQLWDTAGQERFRSLIP  SYIRDSTVAVVVYDITNANSFHQTSKWIDDVRTERGSDVIIMLVGNKTDLSDKRQVSTEEGERKAKELNVMFIETSAKAG  YNVKQLFRRVAAALPGMDSTENKH |
| Aa-36686 | MALNKLSIENVDLKGKRVFMRVDFNVPIKEGKITSNQRIVAALDSIKYALEKGAKSVVLASHLGRPDGNKNAKYTLAPVA  EEMKKLLGRDVTFLNDCVGAEVEAACKDPAPGSVILLENVRFYVEEEGKGVDASGNKVKADKDKVKTFRESLAKLGDVYV  NDAFGTAHRAHSSMMGEGYAQRAAGLLLNKELRYFSQALDNPPRPFLAILGGAKVADKIQLIENLLDKVNEMIIGGGMAF  TFLKVLNNMEIGGSLFDEEGSKIVQKLVDKAKANNVQLHLPVDFVTGDKFAEDAAVGAATVESGIPAGHMGLDVGPKSRE  AFAAPIARAKIIVWNGPPGVFEFPNFANGTKAVMDGVVAATKAGTVSIIGGGDTASCCAKWGTESQVSHVSTGGGASLEL  LEGKVLPGVDALSSA |
| Aa-46286 | MTHRPVSAMSRQESEMINXXRRALSSGTLTDSIEKLRHMCLARGASGILGLGRCFRRMDDDGNKALNLEEFIKGLLDTGL  DITNEEATEMFNKFDTDSSGSINMTEFLIAIRPNMSESRKNIVSQAFAKLDKTGDGAITIEDLKNVYSVKNHPLYISGEE  TEDVILRKFLANFEENGNVDGTVTKEEFLNYYAGLSASIDSDAYFDLMVRQAYKL |
| Aa-47532 | ASISFSTDFSYFAATCSGPTPSYTQIYQTEGQQLIADWETNEQLRTKLAPYKETQVRFLKVPVHGGFEASVRLYLPPEID  FENPANNKETYPMIVQVYGGPNSARVIDTFTVGFGNYLTTTKKTIYCQIDGRGSANQGYDFLFSVNNRLGTVEVEDQIAV  TLQLQETYSFIXXDRTGIWGWSYGGYVTSMALEKDNGSVFKCGISVAPVTSWMFYDSIYTERYMGLPQVQDNEAGYEMAD  VSRYVAGMKNHMFLLIHGNADDNVHYQNSMVFVRALVDEDVDFEQMSYPDEDHGLGGVTQHLYHTMDNFWNQCFA |
| Aa-48005 | MTDANQSLVKNLGLKPLTKENILYYYFPLQSMVSYAALSVNVMNPSIAIRLLPKRDVTNFLLVHTLFGTTLYFYSRPHLA  AVPAQRRAAYSVCGGILFSFGSVLVWAVLRSAIPRNQALATALGLVSGAGLAKLAYDYLDSNDKQLVAKN |
| Aa-48769 | HTDFAEADRALEIHIGTNKQDRVLTVQDTGIGMTKEELIANLGTIARSGSKHFMEQIREKGVSADNAQNIIGQFGVGFYS  AFMVADRVDVYTRSSKVGSPGYKWSSDGSGNFEIQEAENVQIGTKIAIHLKADCREFADEDRIREVIRKYSNFVGSPIFL  NGKHANQIQPIWLMEPKDVTQDQHNEFYRFVGNTFDTPRYTLHYKTDVPLSIRALLYFPEGKPGLFEMSRDADVGVALYT  RKVLIQSKTENLLPKWLRFVKGVVDSEDIPLNLSRELLQNSALIRKLRTVLTNRVLRFLHDRAVKDPENYDKFYKDYGLF  LKEGIVTSQETQEKEEIAKLLRFETSKEEGKKVSLPEYCNSQLEGQKDIYYLAAPNRTLAESSPYYESLKKKGFEVLFCY  EAYDELVLMQLGQFLGKNLVSVEKEMRRSDKADDSSDQPIEGSLLKTQVDELLPWIKQKLTGMVTSVKTTNKLDAHPCVV  TVEEMAAARHFIKTQSHNMSEEHRYALLQPQLEINPKHPIIKKLHKLTTSDPELAELLAKQLFSNAMVGAGLVEDPRMLL  TSMNDLLEKVLDKH |
| Aa-126542 | MCDDDAGALVIDNGSGMCKAGFAGDDAPRAVFPSIVGRPRHQGVMVGMGQKDAYVGDEAQSKRGILTLKYPIEHGIITNW  DDMEKIWHHTFYNELRVAPEEHPVLLTEAPLNPKSNREKMTQIMFETFAAPAVYVAIQAVLSLYASGRTTGIVLDSGDGV  SHTVPIYEGYALPHAILRMDLAGRDLTDYLMK |
| sirot_asb-75251 | AGTAGATATCGAAGGGCATGTTATGGGTCAGGAGGGTATGTTTGCGATCAAGGGCACTGGAGGGTGTTCGCCAGTAGGGACTCCAAAGGAAGTCACCGTATCGATGGCGGGCAAACCATTGCTTCCACGCGTAGTGTATCCGTTGAATGATGGTTCCTTCGAATCCGCTTGCAGCTAGCAAGTATGGAACGGTGCTACCATGTCCGAAAGGATCTATACTCCATCCGCTCTTTGGAGTGACGTTCAGGTTGGTTTTGACCCATTGATGACCTTCGATCAGTTGATCAACCATCGCGTACAGATGAGCATTCGCCTCGTCCGTCATGACCCAACCTCCGGTAGTGATCTCCAATCGACCTGATTTAACCAACTTTTTCAATATCCTCTGCTTGGTGGGATGTGCCTGATCCCACCACAGCTGCAGGAAGCTGATCTCGCTCCAGATGAACGACATGTTGTTATACTCGGGCATCTTGGTGACGGCCAGGTTCAGGATCTGCCGCGAATCCGACTGGAAGTAGTTGACGAAGGTCTTCAACCACCCGGGGTCATTATGCGAGTGCGGCACCACCACAATCTTCAACGGGGGTCGGTTAGGATCCTTCTGCAGCTTCTCATAGCGGTTCTCGAAGTCCTTGTCCCAGTATTCTTTTGTTTTCATCCATTCGGGCTGGAAGTCGAAATTTCCATATTCATCGC |
| sirot_asb-84606 | GATTCAACGTTCTCCGTCTGCAGTTCCTTGAAAAGGGACACCGGCACGTAGGTGACCAGGGTGTACAGCTTGTTGGGCGAAT |

**References:**

1. Megy K, Emrich SJ, Lawson D, Campbell D, Dialynas E, et al. (2012) VectorBase: improvements to a bioinformatics resource for invertebrate vector genomics. Nucleic Acids Res 40: D729–D734. doi:10.1093/nar/gkr1089.

2. Nene V, Wortman JR, Lawson D, Haas B, Kodira C, et al. (2007) Genome Sequence of *Aedes aegypti*, a Major Arbovirus Vector. Science 316: 1718–1723. doi:10.1126/science.1138878.

3. Poelchau MF, Reynolds JA, Denlinger DL, Elsik CG, Armbruster PA (2013) Transcriptome sequencing as a platform to elucidate molecular components of the diapause response in the Asian tiger mosquito *Aedes albopictus*. Physiol Entomol 38: 173–181. doi:10.1111/phen.12016.
